# The role of transcript regions and amino acid choice in nucleosome positioning

**DOI:** 10.1093/nargab/lqad080

**Published:** 2023-09-11

**Authors:** Manish Yadav, Martijn Zuiddam, Helmut Schiessel

**Affiliations:** Cluster of Excellence Physics of Life, TU Dresden, 01062 Dresden, Germany; Institute Lorentz for Theoretical Physics, Leiden University, Leiden, the Netherlands; Cluster of Excellence Physics of Life, TU Dresden, 01062 Dresden, Germany; Institut für Theoretische Physik, Technische Universität Dresden, 01062 Dresden, Germany

## Abstract

Eukaryotic DNA is organized and compacted in a string of nucleosomes, DNA-wrapped protein cylinders. The positions of nucleosomes along DNA are not random but show well-known base pair sequence preferences that result from the sequence-dependent elastic and geometric properties of the DNA double helix. Here, we focus on DNA around transcription start sites, which are known to typically attract nucleosomes in multicellular life forms through their high GC content. We aim to understand how these GC signals, as observed in genome-wide averages, are produced and encoded through different genomic regions (mainly 5′ UTRs, coding exons, and introns). Our study uses a bioinformatics approach to decompose the genome-wide GC signal into between-region and within-region signals. We find large differences in GC signal contributions between vertebrates and plants and, remarkably, even between closely related species. Introns contribute most to the GC signal in vertebrates, while in plants the exons dominate. Further, we find signal strengths stronger on DNA than on mRNA, suggesting a biological function of GC signals along the DNA itself, as is the case for nucleosome positioning. Finally, we make the surprising discovery that both the choice of synonymous codons and amino acids contribute to the nucleosome positioning signal.

## INTRODUCTION

DNA in eukaryotic cells is compacted with the help of proteins into a DNA-protein complex called chromatin (1). The basic unit of chromatin is the nucleosome, consisting of 147 base pairs (bp) of DNA wrapped around an octamer of histone proteins and a stretch of linker DNA that connects to the next nucleosome. The positions of nucleosomes along DNA are not random. This is even the case *in vitro*, where nucleosomes have been reconstituted from DNA and histone proteins and where nucleosomes have been observed to show sequence preferences (2–4). These have been categorized into rotational and translational nucleosome positioning (5). Rotational nucleosome positioning refers to positional preferences within 10 bp, the DNA’s helical repeat. It reflects the fact that DNA is typically curved rather than straight, due to the sequence dependent geometry of its bp steps. Since the DNA is bound at locations where its minor groove faces inward to the histone octamer, there is a preferred position for the nucleosome every 10 bp along the DNA. The precise rules of rotational positioning (GC bp steps at locations where the major groove faces the histone octamer, and TT, AA, and TA bp steps where the minor groove faces the octamer (6,7)) are not straightforward to understand but result from the sequence dependent DNA geometry together with the requirement of sequence continuity (8).

The other type of positioning, translational positioning, is the subject of the current study. It can be seen most clearly when focusing on genome-wide averages of functional sites on genomes, e.g. transcription start sites (TSS’s) (3,9–12), transcription termination sites (9) and intron-exon boundaries (13). Also around nucleosome depleted regions (14,15) (based on data in Refs. (4,16)) translational positioning of nucleosomes occurs. Translational positioning can act in two ways: sequence elements either repel nucleosomes or they attract nucleosomes. For instance, in unicellular organisms, nucleosomes are depleted from the regions before the TSS’s (2,17–20), even *in vitro* where nucleosomes have been reconstituted on genomic DNA (2,20). This has been interpreted to keep promoter regions accessible (3). The opposite is seen in multicellular life forms where nucleosomes are typically attracted to TSS’s (21). However, whereas this is clearly seen *in vitro*, *in vivo* nucleosomes might be depleted from such positions due to the competition with other proteins or due to action of chromatin remodelers, motor proteins that can shift the position of nucleosomes. The attraction of nucleosomes to positions around TSS has been interpreted to play a role in the retention of nucleosomes in sperm cells (22). In spermatogenesis most of the nucleosomes are replaced by protamines which allows the production of large numbers of small, highly mobile cells. Sperm cells retain only a small fraction of the nucleosomes (about 4% (23)) which might allow the transmission of epigenetic marks from father to offspring.

The main rule for translational positioning is simple: nucleosomes prefer DNA with a high GC content, i.e. DNA stretches that carry a large fraction of G’s and C’s (11,12,14,18,24–26). Overall, GC-rich DNA is softer than DNA with a lower GC content (12). Since DNA must bend strongly to wrap about 150 bp’s, the DNA persistence length, almost twice around the histone octamer, the nucleosomes’ preference for the softer GC-rich DNA seems to simply reflect an energetic advantage. However, a recent detailed study (12) shows that the translational sequence preferences of nucleosomes do not reflect elastic bending energies but are instead entropic in origin. To quantitatively predict nucleosome positioning *in vitro* one needs to account for the entropy of free and wrapped DNA. Moreover, since coarse-grained models such as the rigid basepair model only linearly account for the sequence-dependent DNA elasticity, different parametrizations might be necessary to describe the elasticity of the DNA double helix in the free and in the strongly deformed bound states. Finally, evidence suggests that reconstituted chromatin may not be fully equilibrated, due to the slow rate at which nucleosomes reposition themselves. This manifests itself as a constant nucleosome line density at large length scales. According to (12), all these factors have to be considered in order to quantitatively predict translational *in vitro* positioning of nucleosomes in a consistent physical model.

That the nucleosome positions on reconstituted chromatin are not fully equilibrated reflects the fact that there are no fast *in vitro* mechanisms for the redistribution of nucleosomes along the DNA molecules. Mechanisms for thermally induced nucleosome sliding exist but are slow (27). They are based on defects that can spontaneously form at the ends of the wrapped DNA and, if they happen to exit at the other end, cause a corresponding step of the nucleosome along the DNA. Specifically, there are twist defects that carry one extra or one missing bp and loop defects that contain about 10 bp. Both have been predicted in theoretical approaches (28–32) and were observed in computer simulations (33–36). Recent experiments suggest both types of defects as well (37). However, since these mechanisms are energetically costly, *in vitro* nucleosome repositioning is very slow (27) as it has to rely on thermal fluctuations. On the other hand, *in vivo* nucleosome repositioning is typically based on ATP-dependent chromatin remodeling (20,38,39). Some of these chromatin remodelers bind to nucleosomes and use the energy from the hydrolysis of ATP to inject undertwist/overtwist pairs into the nucleosomal DNA. Only this way the high nucleosome densities observed *in vivo* can be achieved (40), but these active processes might drive the nucleosome positions away from the intrinsic bp preferences mentioned above.

In the current study, we focus on the translational sequence preferences of nucleosomes around TSS’s, as observed in *in vitro* nucleosome maps. We ask the question how these intrinsic preferences are encoded in the bp sequence. Upstream of TSS’s, the bp sequences can be chosen freely to encode nucleosomal sequence preferences, but downstream one has to consider that DNA also encodes proteins. This leads to the question whether there is any room for adjusting the GC content downstream of TSS’s. It has been shown, however, that genetic and nucleosome positioning signals can be multiplexed (41) and that there is even room for additional layers of information (42). This reflects the fact that the genetic code is degenerate: 64 codons have to encode for only 20 amino acids. Indeed, this can be used to e.g. rotationally position nucleosomes on DNA with single bp precision, as shown computationally for five positions on the yeast genome in (41) and finally for the whole genome in (43). This set of simulation studies (12,41–43) suggested that the sequence dependent elastic and geometric properties of the DNA double helix strongly affect nucleosome positioning and that these properties can be changed freely through synonymous mutations. On the experimental side, a recent experimental high-throughput study based on the cyclizability of DNA fragments (44) (see also the predictive tool based on this (45)) came to similar conclusions, including the strong role of DNA mechanics for nucleosome positioning and how it is influenced by synonymous mutations.

In these studies, only stretches of bp sequences were considered that were encoding for proteins. However, in the current study arises an additional complexity: regions downstream of the TSS’s do not only contain coding sequences but also non-coding ones, especially the 5’ non-translated region (5’UTR) at the start and various introns that interrupt the coding exons (CDS). Moreover, we define additional regions, namely the 5’ end (located 1000 bp upstream of the TSS) and the 3’ end (comprising the region between the 3’ untranslated region and 1000 bp downstream of the TSS).

The purpose of the current study is to analyse the various contributions to the GC peak around TSS’s. We call this peak a GC signal, assuming that it is a signal meant to attract nucleosomes. We compare the various types of GC signals multicellular organisms produce, and even how similar signals are the result of different mechanisms. We do not ask here why nucleosomes prefer GC-rich DNA, a question we addressed previously (12). Instead we take a purely bioinformatics approach and ask how exactly a GC-rich signal in the sequence of a given organism is encoded for via the various transcript regions. Finally, we also investigate whether the GC signals simply reflect the biased choice of synonymous codons or whether there is even a biased choice of amino acids.

## MATERIALS AND METHODS

### Data

We downloaded the unspliced transcripts with a flanking region of +1 kb and −1 kb from http://www.ensembl.org for vertebrates and from http://plants.ensembl.org for plants using the Biomart RESTful access. The version of the biomart database was Ensembl Genes 106 and Ensembl Plants Gene 53 respectively. We also downloaded the Refseq IDs for each of the transcripts from Biomart server to identify the curation level of the sequences done by the RefSeq database. The transcripts were further labeled with the curation status from RefSeq database (https://www.ncbi.nlm.nih.gov/refseq/). Supplementary Table S1 includes the distribution of transcripts in different curation states and the counts of transcripts that have a length of at least 1 kb downstream of TSS. Each transcript is defined into regions: 5’end (1000 bp upstream of TSS), 5’UTR (5’ Untranslated region), Intron, Coding exon, 3’UTR (3’ Untranslated region) and 3’end (bp’s between the end of 3’UTR and 1000 bp downstream of TSS)

### Data cleaning and formatting

In vertebrates and plants, organisms are classified as ‘selected’ based on their transcripts’ curation status. For vertebrates, this label is applied if over 5$\%$ of their transcripts are marked as PROVISIONAL, REVIEWED or VALIDATED. Meanwhile, for plants, the threshold is raised to 30$\%$. The analysis was conducted only on transcripts with these aforementioned curation levels for the selected organisms. However, for all other organisms, all transcripts, regardless of curation level, were taken into account. Supplementary Table S1 provides a list of the selected organisms along with the percentage of high-quality transcripts.

### GC signal profile

The transcripts were centered at TSS for each organism and the average GC content per base pair position is calculated for positions −1 kb to +1 kb around TSS as follows:


(1) \begin{eqnarray*} \%\text{GC}(p)=\frac{\sum _{t=1}^{T}n_{t,p}}{T} \end{eqnarray*}

where


\begin{eqnarray*} \begin{aligned} &n_{t,p} = \left\lbrace \begin{array}{@{}l@{\quad }l@{}}1 & n_{t,p} = \rm {G} \text{\;or\;} \rm {C} {\;} \text{nucleotide}\\ 0 & \text{otherwise}\end{array}\right.\\ \end{aligned} \end{eqnarray*}

and *p* is the base pair position with respect to TSS and *t* is a specific sequence out of a total of *T* sequences. Equation (1) is called full GC signal. The signal was smoothed using a 3-bp moving average before plotting, and this smoothing was also applied to all subsequent signal plots. The between-region GC content *b*(*p*) for a base pair position *p* is calculated by multiplying the average GC content *a*(*r*) of a region *r* ∈ *R*, where *R* ∈ {5′UTR, CDS, 3′UTR, Intron, 5′end, 3′end} to the density of region *r* at a position *p*. The formula is as follows:


(2) \begin{eqnarray*} {b}(p)=\sum \limits _{r\in R}\frac{\sum \limits _{t=1}^{T}a(r){\;}r_{t,p}}{T} \end{eqnarray*}

where


(3) \begin{eqnarray*} {a}(r)=\frac{\sum \limits _{t=1}^{T}\sum \limits _{p=-1000}^{1000}n_{t,p}{\;} r_{t,p}}{\sum \limits _{t=1}^{T}\sum \limits _{p=-1000}^{1000}r_{t,p}} \end{eqnarray*}

and


\begin{eqnarray*} \begin{aligned} &r_{t,p} = \left\lbrace \begin{array}{@{}l@{\quad }l@{}}1 & \text{if position}\ p\ \text{in sequence}\ t\ \text{belongs to}\ r \\ 0 & \text{otherwise.}\end{array}\right. \end{aligned} \end{eqnarray*}

The within-region signal *w*(*p*) at base pair position *p* is defined as follows:


(4) \begin{eqnarray*} {w}(p)=\sum \limits _{r\in R}\frac{\sum \limits _{t=1}^{T}n_{t,p}{\;}r_{t,p}-a(r){\;}r_{t,p}}{T}\;\mathrm{.} \end{eqnarray*}

The full signal at position *p* is the sum of *b*(*p*) and *w*(*p*). We define the normalised within-region signal $\hat{w}(p)$ for a region *r* ∈ *R*, where *R* ∈ {5′UTR, CDS, 3′UTR, Intron, 5′end, 3′end} at a base pair position *p* as follows:


(5) \begin{eqnarray*} \hat{w}(p, r)=\frac{\sum \limits _{t=1}^{T}n_{t,p}{\;}r_{t,p}}{\sum \limits _{t=1}^{T}r_{t,p}}\;\mathrm{.} \end{eqnarray*}

### CDS within-region signal profile

The coding-exon within-region signal is broken down into the contributions from the amino acid choice and from the choice of the synonymous codons. The amino acid choice signal *c*(*p*) at a base pair position *p* is calculated by multiplying the average GC content *a*′(*x*) of an amino acid *x* ∈ *X*, where *X* is the set of 20 amino acids, with the density of amino acid *x* at position *p*:


(6) \begin{eqnarray*} {c}(p)=\left(\sum \limits _{x\in X}\frac{\sum \limits _{t=1}^{T}a^{\prime }(x){\;}x_{t,p}}{T}\right) - \left(\frac{\sum \limits _{t=1}^{T}a(r)r_{t,p}}{T}\right) \end{eqnarray*}

with *r* = CDS. Here,


(7) \begin{eqnarray*} {a^{\prime }}(x)=\frac{\sum \limits _{t=1}^{T}\sum \limits _{p=0}^{1000}n_{t,p}{\;} x_{t,p}}{\sum \limits _{t=1}^{T}\sum \limits _{p=0}^{1000}x_{t,p}} \end{eqnarray*}

and


\begin{eqnarray*} \begin{aligned} &x_{t,p} = \left\lbrace \begin{array}{@{}l@{\quad }l@{}}1 & \text{if nucleotide at}\ p\ \text{in}\ t\ \text{is contained } \\ & \text{ in any of the triplets encoding}\ x \\ 0 & \text{otherwise.}\end{array}\right. \end{aligned} \end{eqnarray*}

The triplets comprising each transcript are derived from the annotated translation initiation site position, as provided by the Biomart database.

The synonymous codon signal *s*(*p*) at base pair position *p* is defined as follows:


(8) \begin{eqnarray*} {s}(p)=\sum \limits _{x\in X}\frac{\sum \limits _{t=1}^{T}(n_{t,p}{\;}x_{t,p})-(a^{\prime }(x){\;}x_{t,p})}{T}\;\mathrm{.} \end{eqnarray*}

The coding-exon within-region signal at position *p*, one of the terms in Eq. (4), is the sum of *c*(*p*) and *s*(*p*).

### Signal size

Let *S* = *s*_1_, *s*_2_, *s*_3_, …*s*_*p*_ be any GC signal, where *s*_*p*_ is the GC content at base pair position *p* with respect to TSS, and *p* ∈ [1, 1000]. Then the signal size σ_*S*_ for GC signal *S* is defined as


(9) \begin{eqnarray*} \sigma _{S}=\frac{\sum \limits _{p=1}^{1000}\left|s_{p} - \mu _{S}\right|}{1000} \end{eqnarray*}

where


\begin{eqnarray*} \begin{aligned} &\mu _{S} = \text{mean of the GC signal}\ S. \end{aligned} \end{eqnarray*}

Equation (9) offers equal consideration to signals irrespective of their distance from the mean. Unlike, for other quantities, e.g. the standard deviation which amplifies the impact of values further from the mean.

### Clustering

We used the hierarchical clustering method based on Ward’s criterion (46), which aims to minimize the within-cluster sum of squares by merging the two clusters that result in the smallest increase in the total sum of squares at each iteration. The features used to define the clusters were the signal sizes of between-region and constituents of within-region signals. The number of clusters was decided based on the Calinski-Harabasz (CH) (47) Index and silhouette score (48). To investigate the importance of the features in each cluster, we utilized the Random Forest Classifier, a machine learning algorithm that builds an ensemble of decision trees and makes predictions based on the average prediction of each tree (49). For each cluster, we first converted the cluster labels into One-vs-All binary labels, where each class is binary and represents whether a data point belongs to the given cluster or not. We then trained a Random Forest Classifier on the data and extracted the feature importance scores using information gain. Information gain measures the reduction in entropy achieved by adding a particular feature to the decision tree and is used to rank the importance of each feature in each cluster.

### Regression analysis

We determined the signal size for CDS within-region signal, amino acid choice signal, and synonymous codon signal across all vertebrate and plant species. The signal size of the CDS within the region served as our dependent variable, while the latter two as independent variables. We carried out a linear regression analysis for different classes of vertebrates, and exclusively for the Magnoliopsida class in plants. Supplementary Table S2 contains the variable weights. The regression analysis was executed using the scikit-learn python package (50). We also conducted a two-tailed *t*-test to evaluate the significance of the variable weights.

## RESULTS

### Genome-wide GC content characterization around TSS’s

Here we study genome-wide averages of the GC content centered around the TSS’s in a 2000 bp window, see e.g. the black curve in the upper left plot of Figure 2 for the human genome. We systematically break down such signals into their individual components, which we call between-region and within-region signals. The between-region signal is caused by the fact that different regions have on average different GC contents. E.g., alternating exons and introns can lead to a non-uniform average GC profile in the genome-wide average, even in a hypothetical case where the GC content would be perfectly constant within all exons and all introns. The fact that the GC content of any given type of region is never constant as a function of the distance from the TSS (even in the genome-wide average) gives rise to the within-region signal. This signal is especially interesting on coding exons, as this is the fraction of the total signal that shows multiplexing of protein coding and mechanical information for nucleosome positioning.

**Figure 1.**
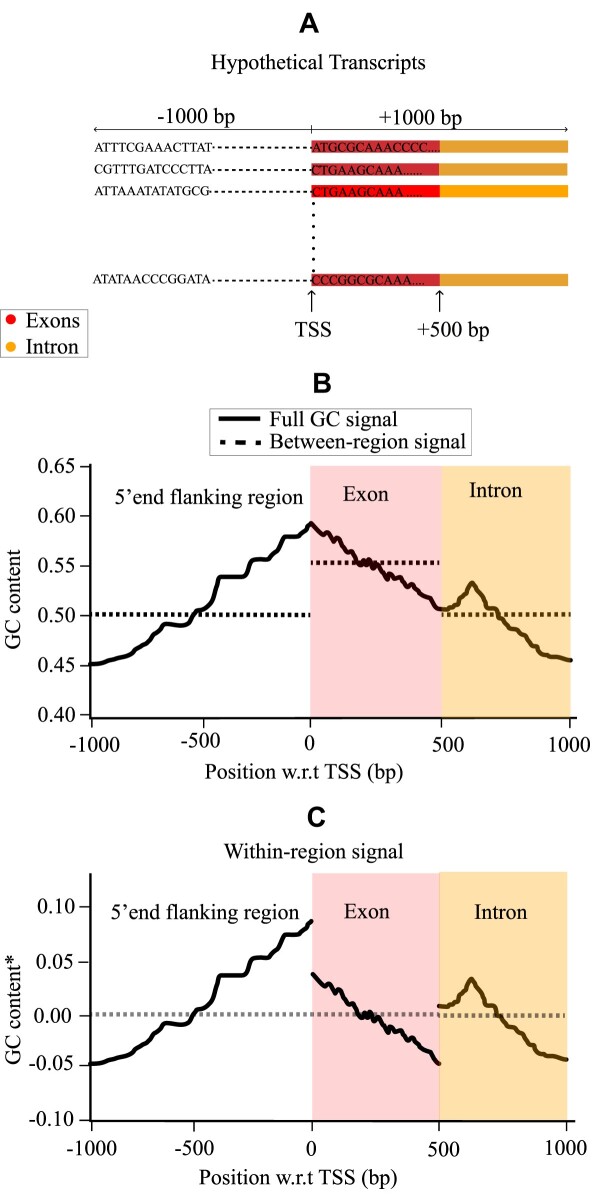
Illustration of the classification of a hypothetical GC signal. This example represents the genome-wide average of the GC signal in a window of 2000 bp centered at TSS. For simplicity, for the hypothetical genome in (**A**) it is assumed that the bp in the non-shaded area belongs to the 5’end flanking region, in the red area only to exons and in the yellow only to introns. The (hypothetical) GC signal from this genome is represented as solid line in (**B**). The dashed line in (B) shows the between-region signal. The between-region signal exists due to differences in the average GC content of introns and exons. (**C**) Within-region signal corresponding to the deviation of the full signal from the between-region signal. It accounts for the non-constant GC profiles within regions. The summation of the between-region signal and within-region signal forms the full GC signal. *Note that the GC content is centered around the between-region GC content and referred to simply as GC content in the further analysis.

**Figure 2.**
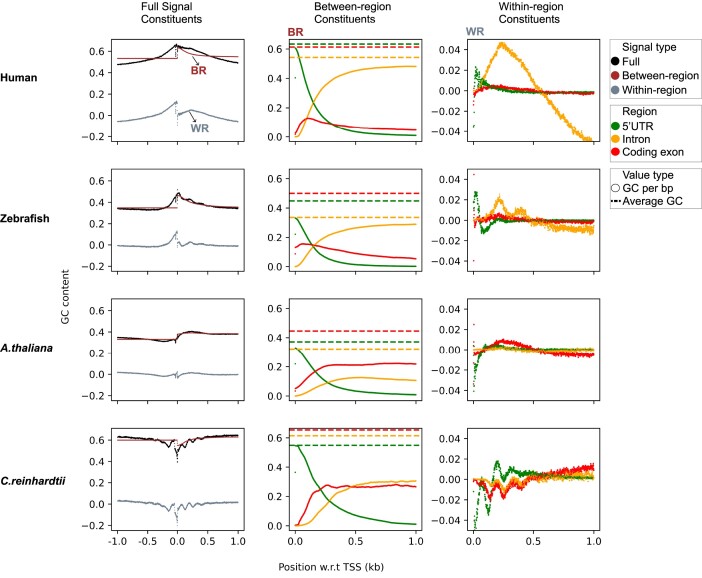
GC signal around TSS for human, zebrafish, *A. thaliana* and *C. reinhardtii*. The plots on the leftmost column show the contributions of both between-region signals (BR) and within-region signals (WR) to the overall GC signals (FS). The plots in the middle column depict the transcript constituents of the between-region signal. The dots represent the GC content per base pair for different regions (as described in Eq. 2, Materials and Methods) and mostly appear as continuous curves. The dashed horizontal lines represent the average GC content values of the various regions (Eq. 3, Materials and Methods). The plots in the right column display the transcript constituents of the within-region signals. Both the middle and rightmost plots only show signals downstream of TSS. The negligible contributions of 3’UTR and 3’end flanking regions to the signal are not shown here and can be found in Supplementary Figure S2.

Figure 1 illustrates the genome-wide average GC content per bp and its breakdown into between-region and within-region signals for a hypothetical genome, centered at the TSS. For simplicity, we assume three types of regions of equal lengths in all transcripts, see Figure 1A. The dashed horizontal lines in b represent the between-region component, which arises from the differences in average GC content between regions. In addition, individual regions contribute via the within-region signal. This signal, which is the deviation of the full signal from the between-region component, accounts for non-constant GC profiles within regions, as seen in Figure 1C. It reflects the freedom of each region to itself incorporate mechanical information. As the within-region signal is centered around the between-region signal, it contains also stretches with negative values. Adding within-region and the between-region components produces the full GC signal.

In the following, we analyze the GC content profile around the TSS’s for vertebrates, plants, yeast, *Drosophila melanogaster* and *Caenorhabditis elegans*. While we focus on the GC signal profile for selected organisms in the next subsections, the supplementary S0 contains the profiles for all the organisms considered in this study.

#### Vertebrates

Here we present the genome-wide GC signal around TSS’s for two vertebrates, human and zebrafish. Studies show that nucleosome positioning in human and zebrafish genomes correlates with intrinsic sequence properties, where high GC rich sequences tend to have higher nucleosome occupancy (4,11,51–53). The top two rows in Figure 2 present the GC signals of these two vertebrates and the breakdown of these signals into their various contributions. The leftmost panels show the signal breakdown of the full GC signal into the between-region and the within-region components. The between-region signal before TSS is constant as one averages only over the 5′ end flanking sequence. Downstream of the TSS, the between-region signal for zebrafish follows quite closely the full signal whereas for humans the two curves differ substantially with the full signal being concave and the between-signal being convex. The difference in curvature is caused by the within-region signal that features a broad peak centered around 250 bp downstream of TSS. In zebrafish, on the other hand, the within-signal is flat overall but shows a wave pattern that decays with distance to the TSS.

An important observation for both species is that both the between-region and the within-region signal show jumps in GC content around the TSS, but the full signal is almost continuous. This suggests that the full GC content might constitute a biologically meaningful signal, possibly for controlling the positions and stabilities of nucleosomes.

The middle panels of Figure 2 show the between-region components broken down into their transcript region constituents. These plots only show the relevant bp region, namely the 1000 bp downstream of the TSS. The curves (each actually a collection of points that appear continuous at the resolution shown) depict the actual contributions of the various transcript regions to the GC profile, while the dashed horizontal lines give their average GC values. Each curve has been calculated by multiplying the density distribution of the corresponding region with the region’s average GC content; the densities alone can be inspected in Supplementary Figure S1. If there were only one type of transcript region at a given bp position, the curve of that region would touch the corresponding dashed line. As can be seen for humans, this is almost the case directly downstream of TSS where the 5’UTR regions dominate. Moving downstream, CDS’s and introns become increasingly important, but further downstream the density of the CDS’s decreases and introns dominate. Because for humans the average GC content of introns is smaller than the average GC content of 5’UTR’s, the overall GC profile of the between-region signal decreases. A similar picture holds for the between-region component of zebrafish, but here the CDS’s play a more important role already directly downstream of TSS.

Finally, the right panels of Figure 2 present the within-region signals in human and zebrafish. Here we observe a dramatic difference between the human and the zebrafish genome. Humans show a broad peak which is mainly caused by the introns together with a very minor contribution from the CDS’s. Zebrafish, on the other hand, shows a wave pattern to which several within-region signals contribute: the first peak is caused exclusively by the 5’UTR’s, the second peak mostly by introns (but there are also minor contributions from 5’UTR and CDS) and the third peak exclusively by introns.

Note that the between-region and within-region components have a rather different origin. The between-region component results from an interplay between different average GC values of the various regions and the nonuniform densities of these regions downstream of TSS. On the other hand, the within-region signals feature one or several peaks positioned at well-defined distances from TSS, to which various transcript regions individually contribute. The latter profile with its peaks emerging on top of a smoother between-region GC profile has thus more of a signal character. But also the between-region component, which results from an interplay of several transcript regions, contributes strongly to the overall GC profile and thus influences positional preferences and the associated stability of nucleosomes.

The GC profiles of other selected vertebrate organisms (chicken, cow, macaque, mouse, pig, rat, orangutan and a species of frog) show similar signaling patterns as the human GC signal, see Supplementary Figure S2. Most notably, all these vertebrate organisms also feature the broad peak around 250 bp downstream of TSS, and the main contribution to this peak always comes from the within-region intron signal. Remarkably, also the largest peak contributed by the introns in zebrafish is at that location (see Figure 2).

**Figure 3.**
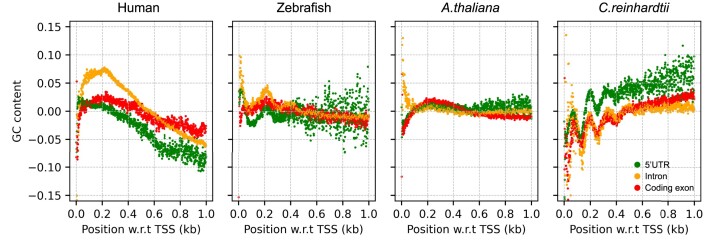
Normalised within-region signal of different regions for human, zebrafish, *A. thaliana* and *C. reinhardtii*. After removing the density effect from the within-region signal, the contributions from introns still dominate in human and zebrafish but also coding exons show substantial contributions. *A. thaliana* and *C. reinhardtii* are less affected by the normalization.

The curves of the various contributions to the full GC signal presented in Figure 2 present the combined effect of density and GC content of the corresponding regions. To learn about the various contributions per bp, we introduce in Figure 3 the normalised within-region signals, which we obtain by dividing the within-region signals from Figure 2 by their densities (see Materials and Methods). For humans, the effect of introns per bp is still large compared to other regions, Figure 3. On the other hand, in zebrafish the normalized intron effect is only slightly more pronounced than the CDS and 5′UTR signals, Figure 3. The wave pattern is still clearly visible, including a contribution from the introns to the first peak which was not visible in Figure 2 due to the low density of introns at that position. Supplementary Figure S3 presents curves of normalised within-region signals of other selected organisms. We observe that in all the selected organisms the introns have the highest per bp contribution of all the regions.

#### Plants

For the examples presented so far, we found only very small contributions of the CDS within-region signals to the full signals. This means that there is not much evidence that multiplexing between protein-coding and nucleosome positioning is important in these genomes, at least on the genome-wide average around TSS. Remarkably, the situation is different for plants, as shown in the following.

The plots in the lower two rows of Figure 2 present the GC signals and their components for the model organism *Arabidopsis thaliana*, a small flowering plant, and *Chlamydomonas reinhardtii*, a unicellular green alga. The GC content for *A. thaliana* shows a peak downstream of TSS following a dip upstream of TSS, whereas *C. reinhardtii* shows quite strong undulations in GC content with several peaks and dips. In *A. thaliana*, the position of the GC signal peak downstream of TSS coincides with the nucleosome occupancy peak mentioned in (54). Also, the GC content in *A. thaliana* is enriched within the core of well-positioned nucleosomes (54). Remarkably, when breaking down the signals into their components in both the between-region and the within-region signals, the contributions from CDS are most dominant in *A. thaliana* and also substantial in *C. reinhardtii*, see Figures 2 and 3, demonstrating the importance of protein coding regions for GC signals in these plants. Additionally, for *C. reinhardtii* the phases of the signal contributions from CDS, 5′UTR and introns are synchronised with each other, see Figure 2, right.

Also for other selected plants, the contributions of CDS to the GC signal are always important and typically higher than the contributions from other regions for both the between-region and within-region signal (Supplementary Figures S2 and S3).

#### Other model organisms

**Figure 4.**
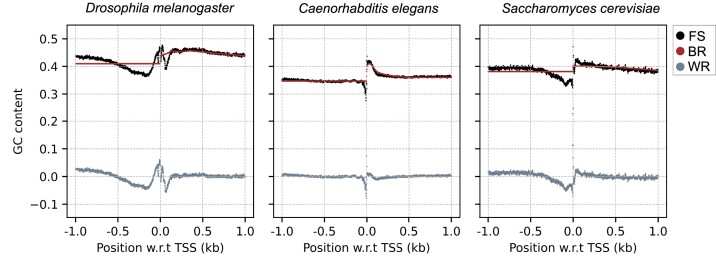
GC signal profiles of *D. melanogaster*, *C. elegans* and *S. cerevisiae*. Each figure shows the full GC signal (FS) around TSS broken down into the between-region (BR) and the within-region components (WR).

It is established that G/C nucleotide enrichment correlates with nucleosome positioning in fly (52,55), worm (11,56,57) and yeast (3,7,11). Figure 4 shows the GC signal analysis for *D. melanogaster* (fly), *C. elegans* (worm) and *S. cerevisiae* (yeast). For all three organisms we find a significant drop in the full GC signal immediately upstream of TSS caused by the within-region signal. Genome-wide studies in these organisms have shown that the upstream region in the vicinity of TSS is associated with nucleosome free regions (NFR) (3,17,52,56). In the case of *D. melanogaster* the GC signal has a Z-shaped form in the close vicinity of TSS, Figure 4(left): The signal first drops at −200 bp and then peaks at TSS and then drops again at +100 bp. The dips in the GC signal downstream and upstream of the TSS are associated with the NFR. As a result, the center of the +1 nucleosome is found 135 bp downstream of TSS (52). For *C. elegans* the nucleosome occupancy maps *in vitro* (57) and *in vivo* (58) align well with the GC signal profile in Figure 4(middle). Also in yeast, Figure 4(right), the upstream region is a NFR and the peak in the GC signal downstream of the TSS is associated with the +1 nucleosome (59).

We also studied the constituents of the between-region and within-region signals for fly, worm and yeast (Supplementary Figure S2). For the fly genome, the between-region contribution to the GC profile downstream of TSS is rather flat, as the strong variations in the various transcriptional regions almost cancel each other. The presence of a peak immediately followed by a dip in this region is mainly caused by the within-region 5’UTR signal. In worm, the GC signal shows a prominent peak downstream of TSS which reflects a peak in the CDS density, the transcriptional element with the highest average GC content. This is also consistent with the observation in (57) that exons are intrinsically more susceptible to nucleosome formation compared to introns. The within-region signal is rather flat. In the case of yeast, transcripts lack the 5’UTR and introns play a minor role. Due to the short lengths of many genes, the CDS contribution is partially taken over by the 3’ end within the 1000 bp window considered here. As their respective average GC contents are similar, the resulting between-region profile is rather flat.

### Clustering of organisms by GC signal

In the previous section, we found large differences between the GC signals around TSS of different organisms, especially in the way these signals came about through the combination of the various between-region and within-region components. Based on this observation we compare here the signals for a large number of organisms, namely 211 vertebrates. To make this comparison feasible, we project the full signal and its contributing elements onto scalars. Specifically, we introduce the concept of signal size, defined as the deviation of the signal from its mean signal. The details of this definition are explained in Methods. Note that this approach has a cost in that we loose information about the specific shape of the signals, but it allows us to compare large numbers of organisms in one diagram.

We use a clustering method called hierarchical clustering to group together vertebrates whose full GC signal results from similar combinations of sub-signals. The clustering algorithm takes into account six parameters, namely the signal sizes of the between-region signal and the constituents of the within-region signal for each organism. We exclude signal contributions from the 5’ end flanking region for clustering because we focus here on the region downstream of TSS. The Calinski-Harabasz score (47) and the silhouette score (48) indicate that the optimal number of clusters for the 211 vertebrate organisms is two, as both scores maximize this number of clusters (Supplementary Figure S4).

**Figure 5.**
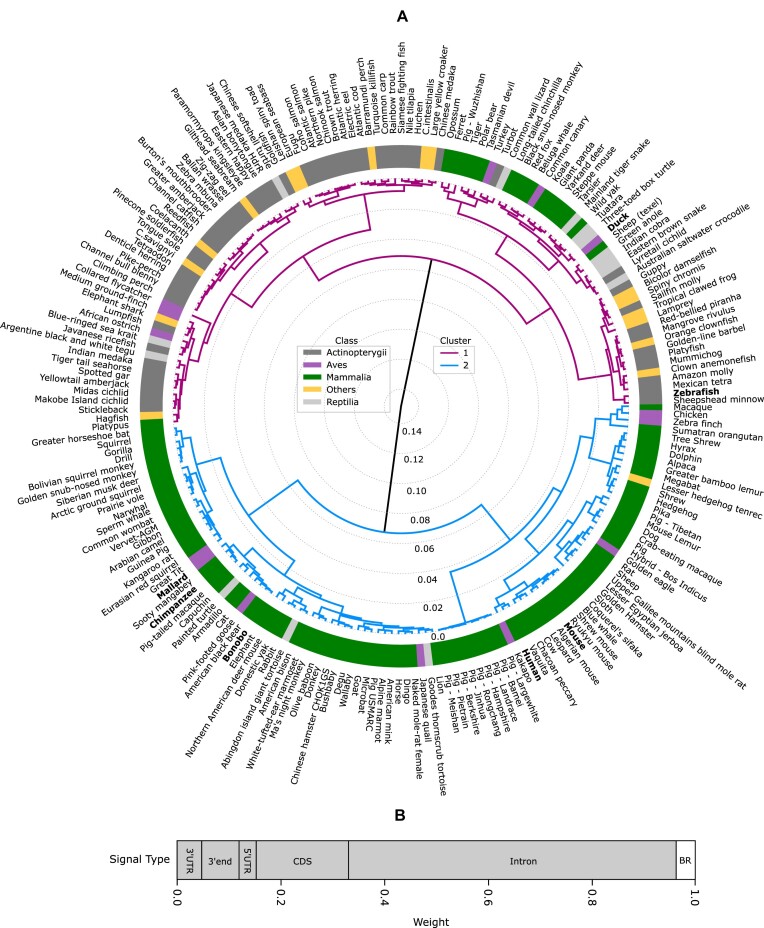
Two clusters of vertebrate organisms. (**A**) The diagram shows the clusters formed by the hierarchical clustering algorithm based on Ward’s criterion using the signal size of the between-region signal and the within-region constituent signals. The values give the level of dissimilarity (distance) between the organisms/clusters. The color of the lines represents the cluster and the color of the boxes the taxonomic class. Ascidiacea, Amphibia, Chondrichthyes, Actinopteri, Myxini and Hyperoartia are grouped as ‘Others’ due to the low number of species in each of these classes. The height of the branches indicates the dissimilarity between clusters. (**B**) Stacked histogram illustrating the relative importance of the different signals for the formation of the clusters. The shaded area indicates the constituents of the within-region signal and emphasizes their importance over the between-region (BR) signal (white).

Figure 5A shows the two clusters with dissimilarity distances. Cluster 1 mainly contains the Actinopterygii class, but interestingly it has some mammalia species as well. Cluster 2 contains mostly the mammalia class. Contrary to expectation, the species belonging to the same classes are not always grouped closely. For instance, duck and mallard both belong to the Aves taxonomic class and are genetically closely related but fall into different clusters. Duck here refers to the domestic duck, which is a descendant of the mallard when the mallard has been domesticated in China some 3000 years ago (60). Similarly, chimpanzee and bonobo, the living species closest to humans, are not closely grouped with humans.

To gain a better understanding of these observations, we investigated the contributions of the various signal sizes to the formation of the clusters. Figure 5B shows that the majority of the cluster segmentation depends on the signal size from the intron within-region signals ($63\%$), followed by signal size of the coding exon within-region signal ($18\%$). The between-region signal has only about ($4\%$) contribution in the formation of the clusters. This also explains why mallard and duck are not grouped closely as they differ in their intron within-region signals (see Supplementary S0). A similar difference is also observed for humans, chimpanzees, and bonobos, with humans having different intron within-region signals than chimpanzees and bonobos.

If instead we take only two parameters, the total between-region and within-region signals, to define the clustering, we observe a different grouping of the species (see Supplementary Figure S5). Duck and mallard are now grouped together but human is still not grouped with chimpanzee and bonobo. The grouping is dominated by the within-region signal, which contributes $92\%$ to the clustering. In fact, chimpanzees and bonobos have similar total within-region signals that differ from the corresponding signal in humans. Also duck and mallard have similar total within-region signals but the individual components differ, explaining why they are far in the diagram in Figure 5A but close in the diagram in Supplementary Figure S5.

We also conducted a similar analysis for plant species and identified two distinct clusters (Supplementary Figure S5). Similar to the findings in vertebrates, genetically related plant species are not always grouped together, indicating that the clustering is not solely based on genetic relatedness. Interestingly, we found that in plants, the difference in between-region signal contributed significantly (about $45\%$) to the grouping of species. This is in contrast to vertebrates, where the between-region signal contribution is very small due to its similarity across species. For example, *Galdieria sulphuraria*, despite being closely related to *C. reinhardtii*, is clustered with *A. thaliana* due to the difference in their between-region signals, which is also reflected in their GC signal (see Supplementary S2). This highlights the importance of considering the between-region signal in plant species for accurate clustering.

Overall, our results provide novel insights into the diversity of GC signal profiles in vertebrates and plants. Even when they are evolutionary closely related, species create different total GC signals around TSS and, even similar total signals are obtained by prioritizing different transcript elements. Further investigations are necessary to understand the mechanisms underlying these similarities and differences.

### Intron effects on GC signals

So far we have considered the GC signal as a signal on the DNA. However, it could be that the signals are not intended for the DNA but for the mRNA instead. Of particular interest is here our finding, reported above, that introns are the main contributors to the GC signal in vertebrates but fall behind CDS and 5’UTR regions in plants. Do the GC signals get stronger or weaker when the introns are removed from the pre-mRNA transcripts? And is the behavior of vertebrates and plants the same or opposite in this regard? Should it be the case that signals increase with intron removal, one could speculate that the GC signal on the DNA is just a side effect of a signal on the mRNA where it might serve some other function, e.g. regulating translation speed in ribosomes, which would affect cotranslational protein folding (42). For the following analysis, we only consider the region downstream of the TSS, since the upstream region does not contribute to the mRNA GC signal.

#### Vertebrates

**Figure 6.**
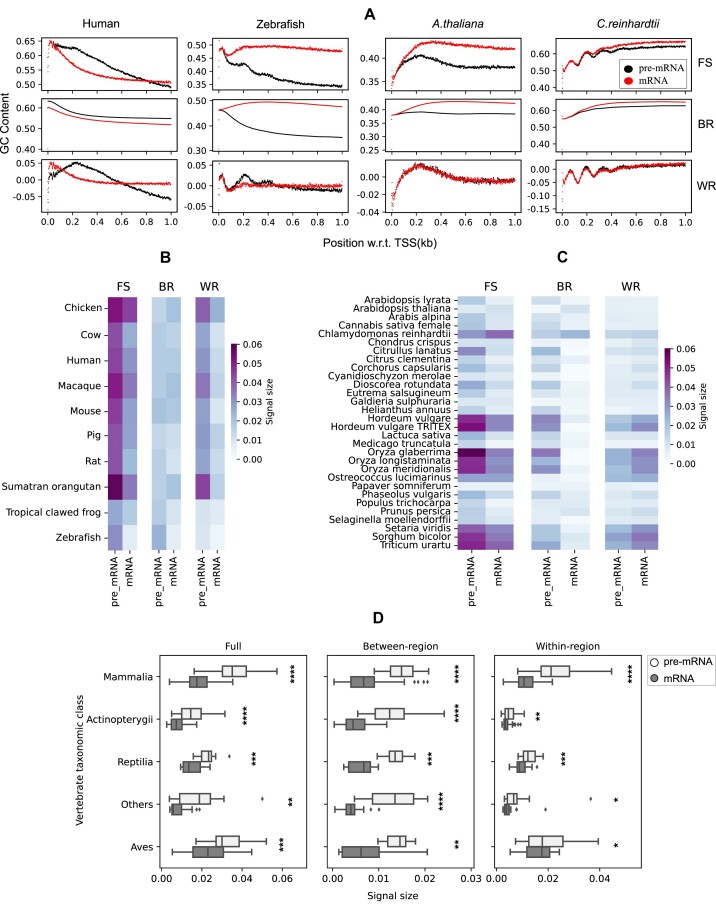
Signal size comparison between pre-mRNA and mRNA sequences for vertebrates and plants. (**A**) GC signal comparison between pre-mRNA and mRNA sequences downstream of TSS for human, zebrafish, *A. thaliana* and *C. reinhardtii*. (**B**) Heatmap of full, between-region and within-region signal size between pre-mRNA and mRNA for selected vertebrates. FS: full signal, BR: between-region signal and WR: within-region signal. (**C**) Same as (B) but for selected plant species. (**D**) Signal size comparison between pre-mRNA and mRNA sequences across vertebrate taxonomic classes. Wilcoxon ranked sign test was used to compute the *P*-values between the groups. *P < 0.05; **P ≤ 0.01; ***P ≤ 0.001; ****P ≤ 0.0001.

Figure 6A shows the comparison between the signal profiles of pre-mRNA and mRNA sequences for human and zebrafish. In humans, the full GC signal decays faster for the mRNA than for the pre-mRNA. At the same time, the between-region signal shape does not change much, while the within-region signal loses its peak around 250 bp downstream of TSS. In contrast, the genome of zebrafish shows the opposite effect on the signal profile when going from pre-mRNA to mRNA as the full signal flattens and shows a higher GC content. This is closely mirrored by the between-region signal. Finally, removing the introns for the within-region signal leads to a loss of the characteristic wave pattern. Supplementary Figure S6 provides comparisons for other selected vertebrates which typically exhibit similar behavior as for the human genome.

In Figure 6B, we compare pre-mRNA and mRNA signal sizes for human, zebrafish and eight other vertebrates. For the full signal, the signal strength is reduced in the mRNA sequences compared to the pre-mRNA sequences. This observation typically applies to other vertebrates as well, see Supplementary Figure S7. The between-region signal size is also weaker for mRNA compared to pre-mRNA for the selected organisms, except for chicken, macaque and orangutan. Also in other vertebrate organisms, the between-region signal is typically weaker in the mRNA sequence (Supplementary Figure S7). Within-region signal sizes show the same trend, with the mRNA signal being weaker than the pre-mRNA signal in almost all of the organisms, cf. Figure 6B and Supplementary Figure S7.

To get a more general overview, Figure 6D shows the distributions of the full, the between-region and the within-region signal sizes for various vertebrate taxonomic classes, comparing pre-mRNA and mRNA sequences. These distributions are consistent with the behavior we observed for individual organisms, namely that the full, the between-region and the within-region signal sizes are smaller on average for mRNA sequences than for pre-mRNA sequences. Note that in general, Mammalia and Aves classes have higher signal sizes for both sequences compared to other classes.

Although introns contribute most significantly to the GC signals downstream of TSS in most vertebrates (see Figures 2 and 5), removal of the introns, going from pre-mRNA to mRNA, weakens the GC signal. This suggests that in vertebrates the GC signal on mRNA is an attenuated and scrambled version of the original signal present on the DNA sequence, where it might serve to influence nucleosome positioning and stability around TSS.

#### Plants

The right two columns in Figure 6A show a comparison between pre-mRNA and mRNA sequences centered on TSS for *A. thaliana* and *C. reinhardtii*. For both organisms, the within-region signal remains almost the same while there is a change in the full signal caused by a change of the between-region signal. Since CDS and 5’UTR regions contribute most to the full signal in pre-mRNA and both regions are also present in the mRNA sequence, the changes in the signals are not significant.

Figure 6C provides a comparison between 30 selected species (including *A. thaliana* and *C. reinhardtii*). In most cases, the full signal sizes and the between-region signal sizes are higher in pre-mRNA sequences than in mRNA sequences. On the other hand, the within-region signal size is typically slightly higher in the mRNA sequences compared to pre-mRNA. However, this tendency is always strongly compensated by the between-region signals. These observations can also be made in Supplementary Figure S7, which provides a comparison of all the plants considered here. Unlike vertebrates, we have not grouped plants into taxonomic classes because the plants species mostly belong to the Magnoliopsida class, see Supplementary Table S2 for the exact distribution of the species by taxonomic classes. Nevertheless, the Magnoliopsida class shows a significant difference between DNA sequence and mRNA sequence for the full signal, between-region and within-region signal [Wilcoxon ranked sign test, *P* < 0.0001].

Overall, as in vertebrates and plants, GC signals are stronger on the DNA and removal of introns weakens the signal, suggesting that GC signals might be biologically relevant on the DNA. This is also consistent with the observation that the DNA GC signal has for some genomes additional features, e.g. the characteristic undulations for the zebrafish GC signal that disappear when going to mRNA, see Figure 6A. In addition, the fact that GC levels just upstream of TSS match with GC content just downstream of TSS, see Figure 2, supports this notion.

### Amino acid effect on GC signals

In this section we focus on the within-region signal of CDS’s. This is of particular interest because coding exons code for proteins and the question arises how they can also carry a GC signal. As mentioned in the introduction, this is possible in principle because the genetic code is degenerate. For the 18 out of 20 amino acids that have more than one synonymous codon, there is always the possibility to change the GC content by switching between synonymous codons. However, there is an alternative possibility to generate a within-region CDS signal: exchanging amino acids. It is known that many amino acids can be exchanged without affecting protein folding (61–63). For the sake of the argument assume that each codon in a set of synonymous codons is used equally likely. Then a change in GC content at a given codon position can be achieved by changing from one amino acid to another amino acid that has a different average GC content and similar physiochemical properties. We define this average for a given amino acid as the fraction of G’s and C’s of its set of synonymous codons. This study focuses on the contribution of amino acid choice as a whole to the formation of the GC signal, without breaking the signal down into the individual amino acid contributions. In the future we plan to investigate whether an exchange between amino acids of similar physiochemical properties has occurred in the evolution of genomes.

To determine the two possible contributions to the CDS within-region GC signal, we use a similar scheme as before, namely we split the signal into its contributions. One contribution is the amino acid choice, where we assign to each amino acid the average GC content of its set of synonymous codons, and the other contribution accounts for the bias within the set of synonymous codons (see Materials and Methods for details).

#### Vertebrates

**Figure 7.**
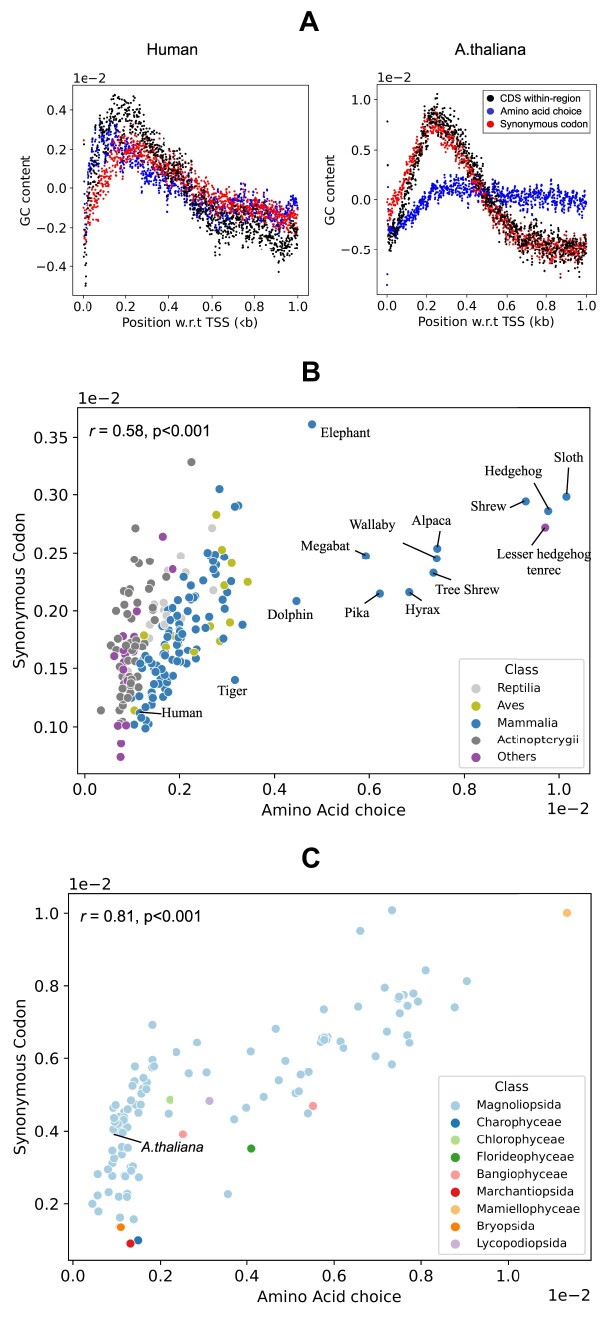
Contributions to the CDS within-region signal. (**A**) Synonymous codon bias (red) and amino acid choice (blue) both contribute to the full CDS within-region signal (black) in humans but synonymous codon choice dominates in *A. thaliana*. (**B** and **C**) Signal size correlation between the amino acid choice and synonymous codon bias for all vertebrates and plants respectively. *r*: Pearson coefficient, *P*: *P*-value.

The plot on the left of Figure 7A shows the human CDS within-region signal (black) and the contributions from amino acid choice (blue) and from synonymous codon bias (red). Remarkably, both sub-signals contribute approximately equally to the CDS within-region signal. The profiles for other vertebrates are presented in Supplementary Figure S8.

We investigated the correlation between amino acid choice and synonymous codons signal size for all vertebrates that we considered in this study. Figure 7B presents the signal strength of the amino acid choice versus the signal strength of the synonymous codon bias. These two quantities are positively correlated (*r* = 0.58, *P* < 0.001). We find that the relationship between amino acid choice and synonymous codon signal size varies taxonomically. While the synonymous codon bias largely influences the CDS within-region signal in Mammalia, exceptions like Alpaca and Megabat show a dominant amino acid choice. In Aves, the amino acid choice mostly shapes the CDS within-region signal. However, the synonymous codon bias is more prominent in Actinopterygii, Reptilia, and ‘Others’ groups. Supplementary Table S2 further solidifies these conclusions with weights of amino acid choice and synonymous codon contributions derived from regression analysis.

#### Plants

The plot on the right of Figure 7A shows the contributions for the CDS within-region signal for *A. thaliana*. In contrast to human, the synonymous codon bias is mainly responsible for the CDS within-region signal. Looking at all available plant species in Figure 7C, we find, as in vertebrates, a positive correlation of the signal sizes between amino acid choice and synonymous codon bias with *r* = 0.81 and *P* < 0.001. Moreover, the CDS within-region signal is primarily formed by either approximately equal contributions from the amino acid choice and synonymous codons, or mostly by synonymous codons with a minor contribution from the amino acid choice. The contribution of the amino acid choice is generally not dominant in plants, which is in contrast to vertebrates (Supplementary Table S2).

In summary, GC signals on coding exons have two different ways of encoding GC content. The first possibility consists of exploiting the degeneracy of the genetic code and influencing the GC content by choosing between synonymous codons. This is an example of multiplexing between protein sequence information and nucleosome positioning, as previously discussed (41–43). The second option makes use of the fact that some amino acids can be exchanged without affecting protein folding and adjusts the GC content by choosing amino acids that have synonymous codons with an appropriate GC content on average. Remarkably, depending on the vertebrate class, either the first or the second option dominates the within-region CDS signal. On the other hand, plants either use both contributions equally or mostly use synonymous codon bias to create GC signals.

## DISCUSSION

In this study, we analyzed the various contributions from transcriptional elements (mainly 5’ UTR’s, coding exons and introns) to the peaks in GC content observed around transcription start sites (TSS) in genome-wide averages of multicellular organisms. The motivation behind this study is that nucleosomes exhibit preferences for certain base pair sequences, including a preference for GC-rich DNA. Note, however, that individual nucleosomes near a TSS can only ‘see’ the specific base pair sequence around that particular TSS and do not care about genome-wide averages. This leads to the question whether genome-wide averages are useful quantities to look at.

To learn more about this problem, we developed a classification scheme by decomposing the genome-wide GC signal into two types of contributions from transcriptional elements: between-region and within-region signals. Between-region signals reflect the fact that different transcriptional element may have different average GC content, while within-region signals account for possible inhomogeneities in the average GC content within given elements as a function of distance from TSS. We found that both types of signals are present in the various genomes considered here. This is important as it shows that GC peaks are not only a consequence of the densities of the different transcriptional elements but that there are systematic within-region contributions as seen in Figure 3, which shows normalized within-region signals. This suggests that genome-wide averaging is indeed a useful approach to learn about nucleosome positioning effects around TSS.

More specifically, in this study we first performed an analysis for two vertebrates and two plants and found large differences in the various contributions to the signals between vertebrates and plants. We found also large differences between the two vertebrates and between the two plants, but there were also similarities. What both vertebrates have in common is that the contributions from the introns dominate, while for the plants the contributions from the coding exons are most important.

Next, we presented an overview of the GC signal strengths for all available vertebrates by performing a cluster analysis, finding two clusters for the vertebrates. The important factors distinguishing the clusters are between-region and within-region signals from introns. We observed in our analysis that some vertebrate classes stand out, especially Actinopterygii, which mostly belong to one cluster. In general, however, evolutionary closely related species show often large variations in their signal contributions. As a result, these species even belong to different clusters.

We also addressed the question of whether the GC signal is meant for the original DNA base pair sequence or has a potentially more important function downstream in the production of the proteins. To do this, we compared signal strengths on sequences with and without introns, i.e. on pre-mRNA and on mRNA. This analysis indicated that signal strengths are stronger on pre-RNA, suggesting that the TSS GC-peaks have more likely a biological function along the DNA, possibly to control nucleosome positioning and stability.

Finally, we focused on one particular contribution, namely the within-region signal in coding exons. This is of particular interest since coding exons encode for proteins but also show a non-vanishing within-region signal that contributes to the GC signal. This type of multiplexing is possible because of the degeneracy of the genetic code. Remarkably, however, we found for the human genome that only about half of the signal stems from the biased choice of synonymous codons. An equally important contribution comes from the choice of amino acids which is especially strong closer to the TSS. For many mammals the amino acid choice is even the dominant contribution whereas in plants the opposite is typically the case.

The observation that the amino acid choice contributes to the CDS within-region signal raises the possibility that codon usage bias and amino acid sequence may play a role in nucleosome positioning in these organisms. Further investigation is needed to determine the biological significance of these findings and their potential implications for nucleosome positioning in eukaryotes. In this context it will be useful to study this signal for different classes of genes in various organisms. One can also apply our classification scheme to other genomic landmarks, e.g. to intron-exon boundaries.

## Supplementary Material

lqad080_supplemental_files

## Data Availability

The underlying data used for this work is fully available on the Biomart database (https://www.ensembl.org). Specifically, the version employed for vertebrates was ‘Ensembl Genes 106’, while the version for plants was ‘Ensembl Plants Gene 53’. The procedure to download the sequences is explained in the supplementary file ‘Data_Acquisition.zip’. Any additional information and analysis scripts are available upon request.
