# Supplementary material for: The role of transcript regions and amino acid choice in nucleosome positioning
Source: NAR Genom Bioinform. 2023 Sep 11;5(3):lqad080. doi: 10.1093/nargab/lqad080 (PMC10495542; doi:10.1093/nargab/lqad080)
Supplement: lqad080_supplemental_files [file lqad080_supplemental_files.zip › manuscript_supplementary.pdf]

Figure S1

Densities of transcript elements within a window of 2 kp centered at TSS for selected vertebrates and plants. Plots show the densities of 5'end flanking regions (cyan), 5'UTR's (green), introns (orange), coding exons (red), 3'UTR (blue), and 3'end flanking regions (gray). The density upstream of TSS is one due to the presence of only 5'end flanking regions.

Vertebrates

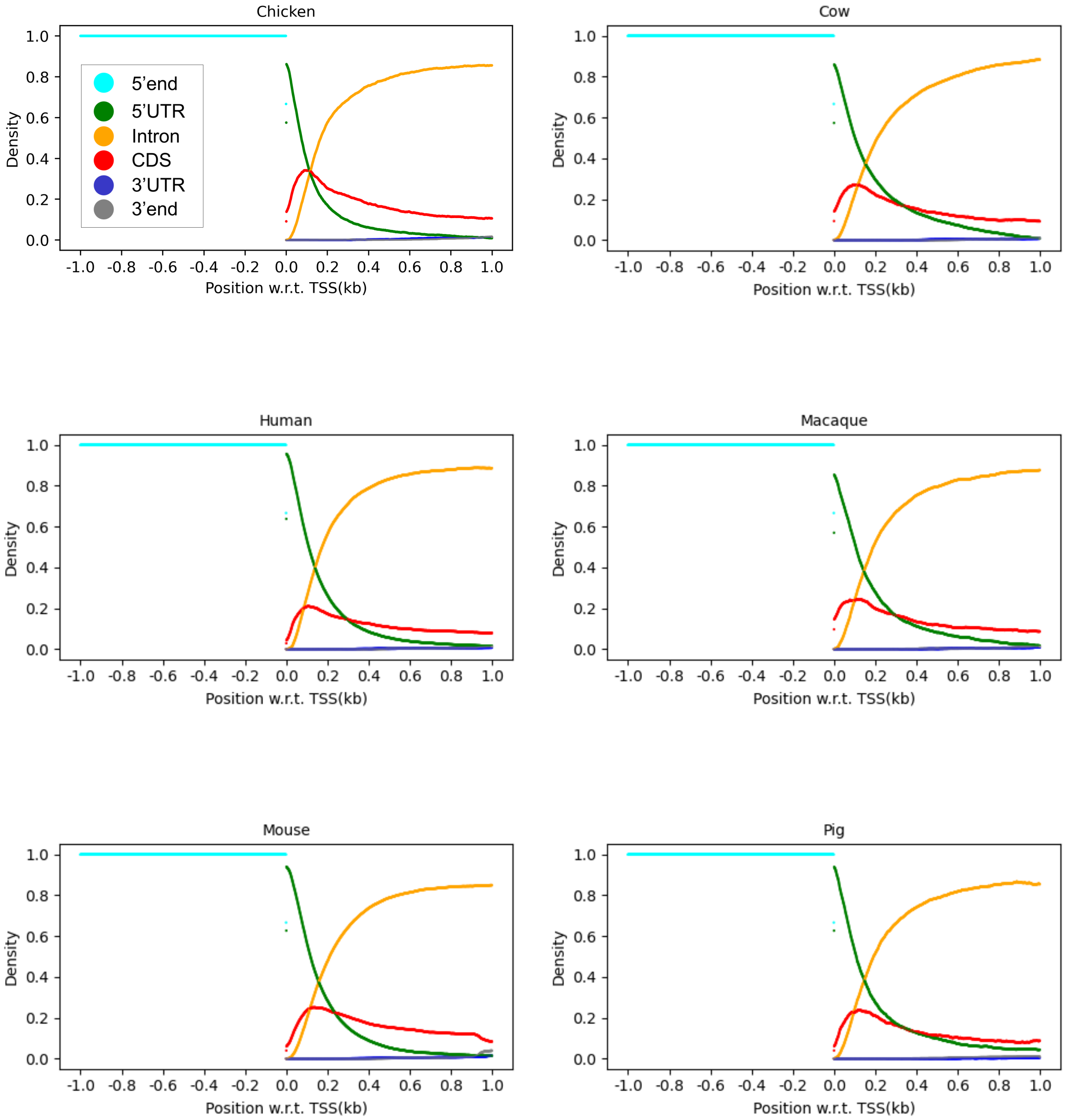

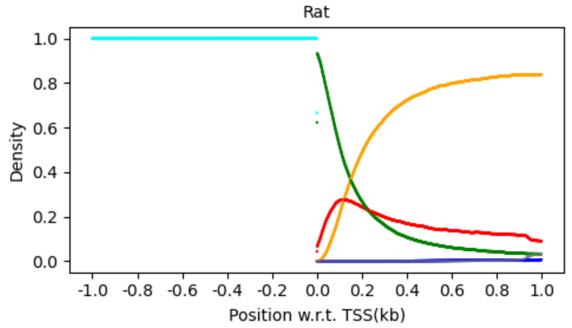
Rat
Density
Position w.r.t. TSS(kb)

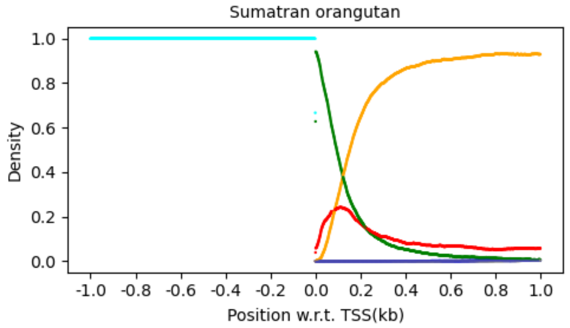
Sumatran orangutan
Density
Position w.r.t. TSS(kb)

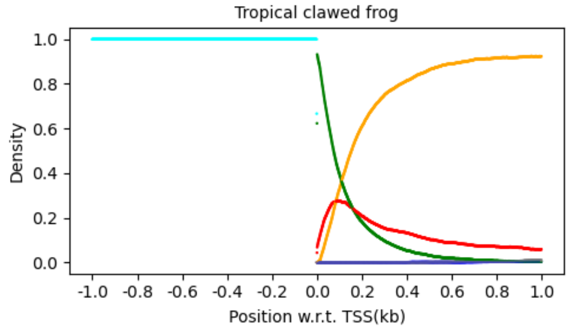
Tropical clawed frog
Density
Position w.r.t. TSS(kb)

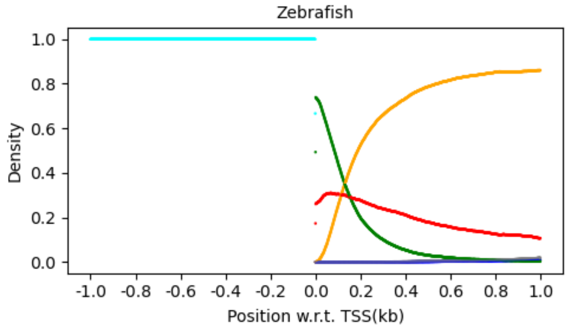
Zebrafish
Density
Position w.r.t. TSS(kb)

Plants

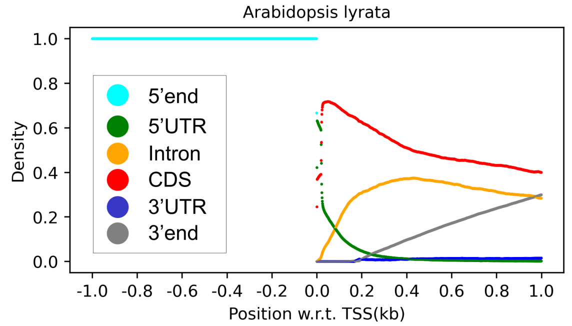
Arabidopsis lyrata
5’end
5’UTR
Intron
CDS
3’UTR
3’end
Density
Position w.r.t. TSS(kb)

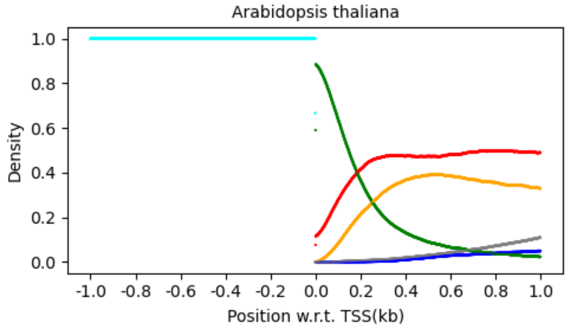
Arabidopsis thaliana
Density
Position w.r.t. TSS(kb)

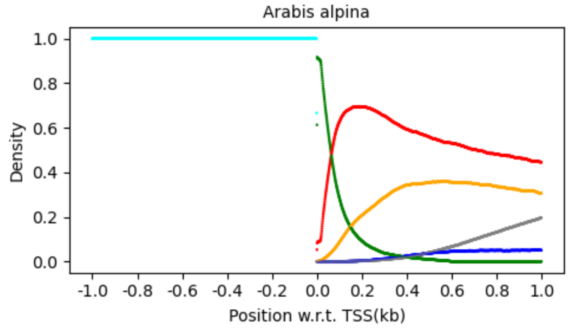
Arabis alpina
Density
Position w.r.t. TSS(kb)

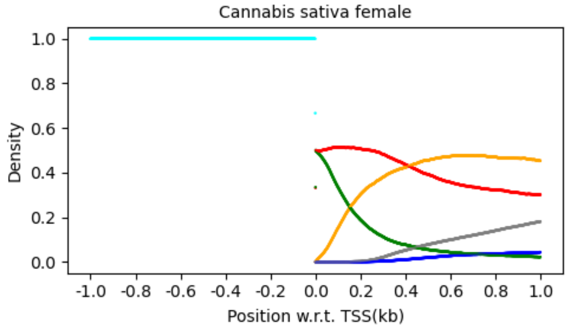
Cannabis sativa female
Density
Position w.r.t. TSS(kb)

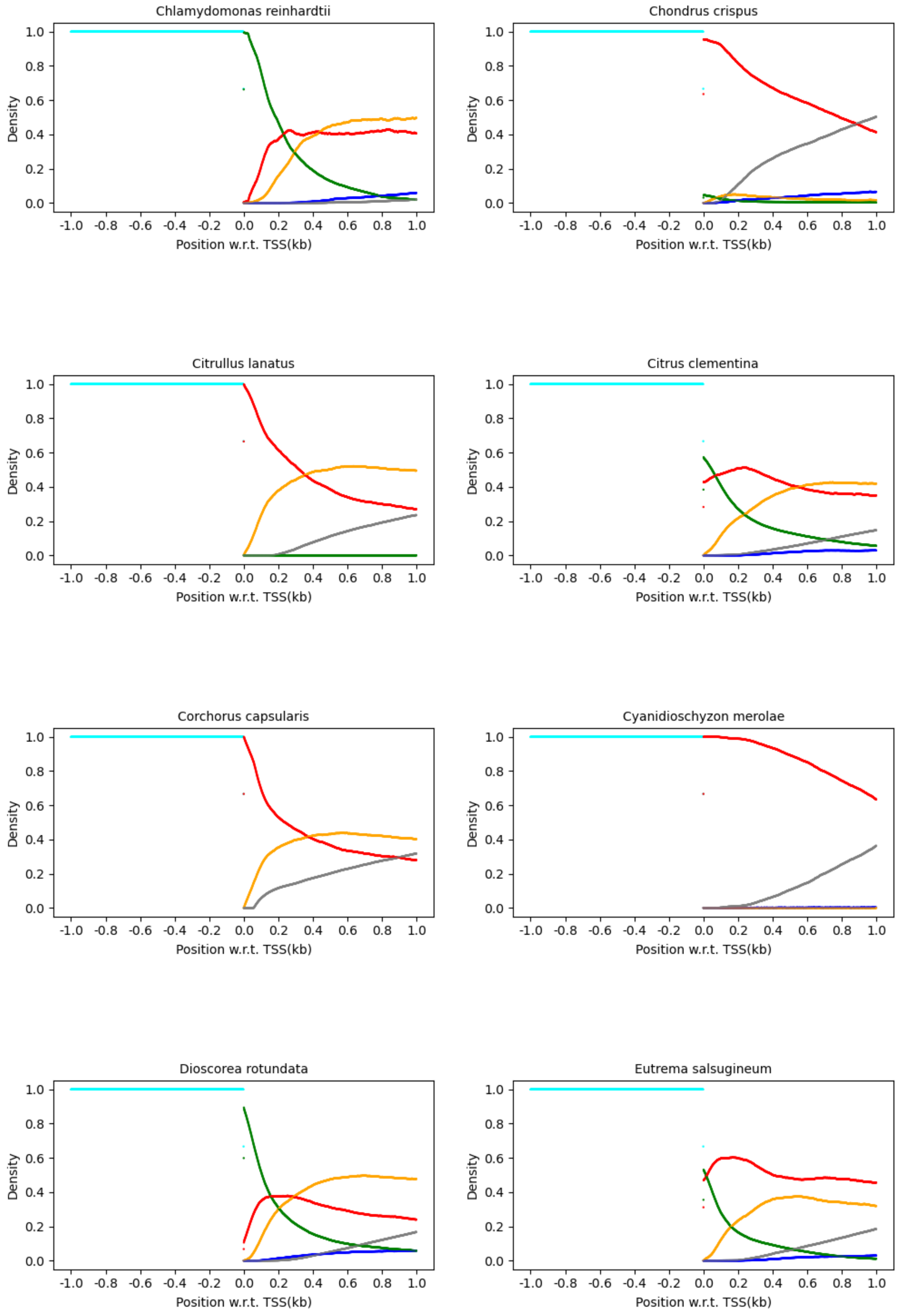
Chlamydomonas reinhardtii
Chondrus crispus
Citrullus lanatus
Citrus clementina
Corchorus capsularis
Cyanidioschyzon merolae
Dioscorea rotundata
Eutrema salsugineum
Density
Position w.r.t. TSS(kb)
1.0
0.8
0.6
0.4
0.2
0.0
-1.0
-0.8
-0.6
-0.4
-0.2
0.0
0.2
0.4
0.6
0.8
1.0

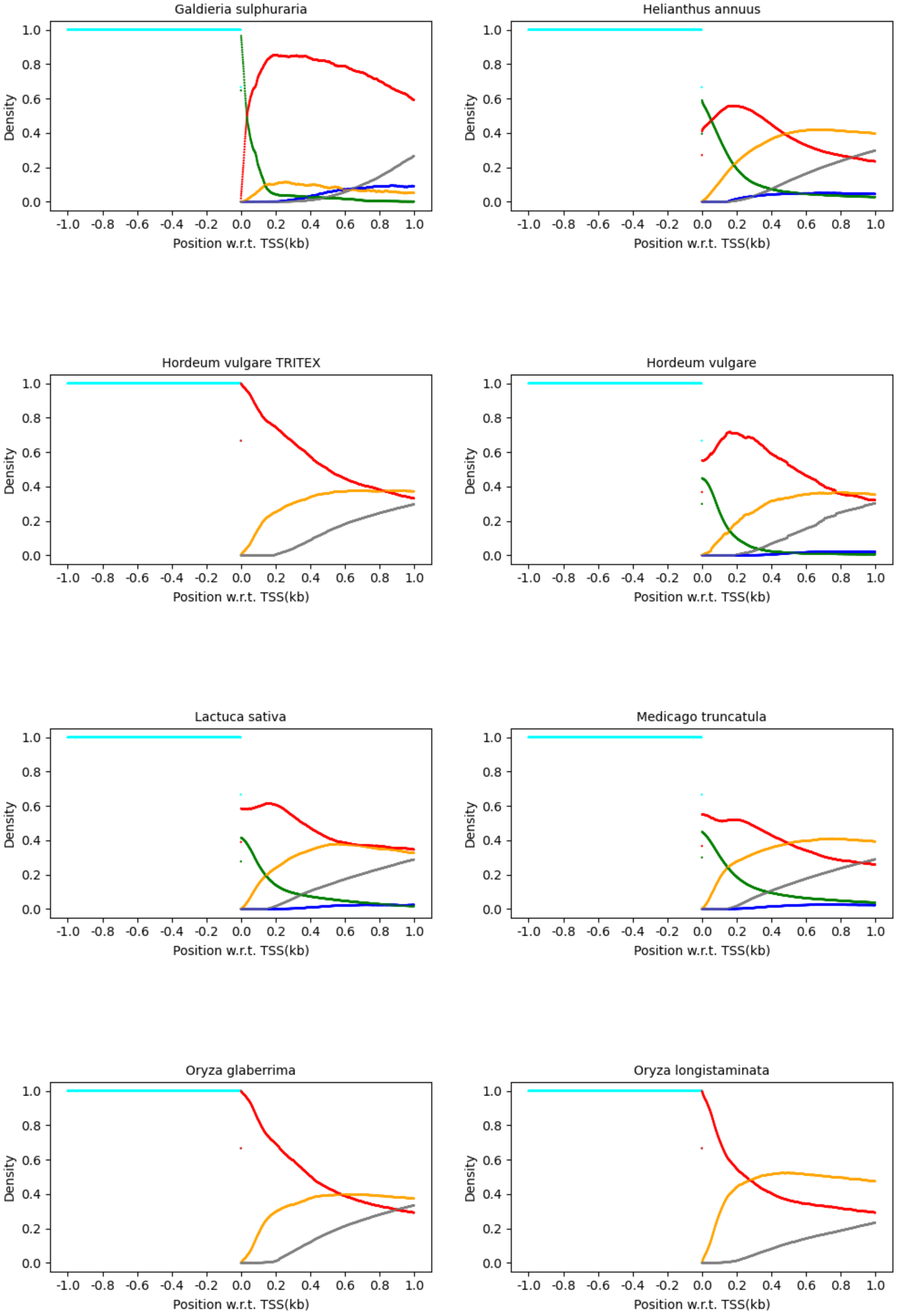
Galdieria sulphuraria
Helianthus annuus
Hordeum vulgare TRITEX
Hordeum vulgare
Lactuca sativa
Medicago truncatula
Oryza glaberrima
Oryza longistaminata
Density
Position w.r.t. TSS(kb)

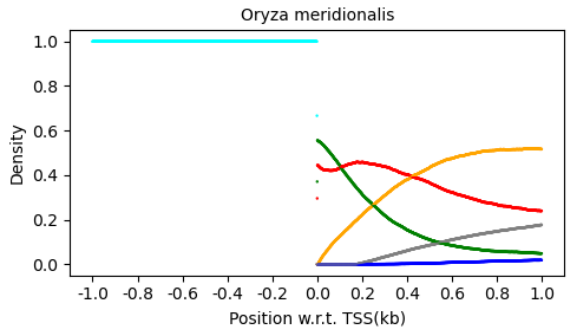
Oryza meridionalis
Density
Position w.r.t. TSS(kb)

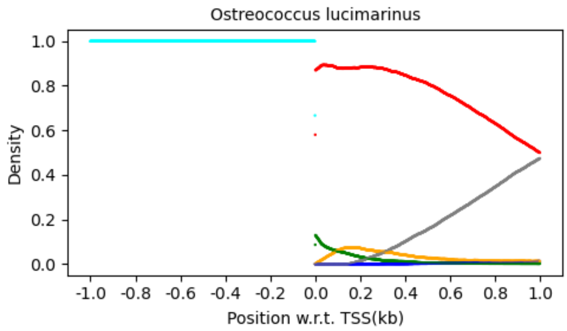
Ostreococcus lucimarinus
Density
Position w.r.t. TSS(kb)

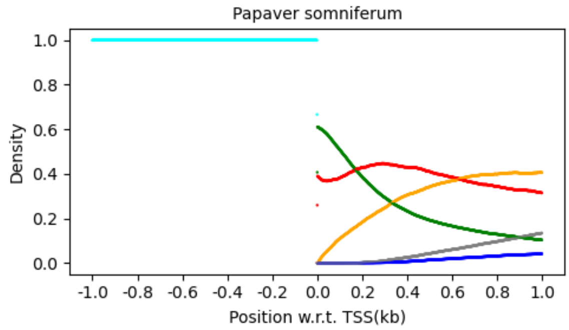
Papaver somniferum
Density
Position w.r.t. TSS(kb)

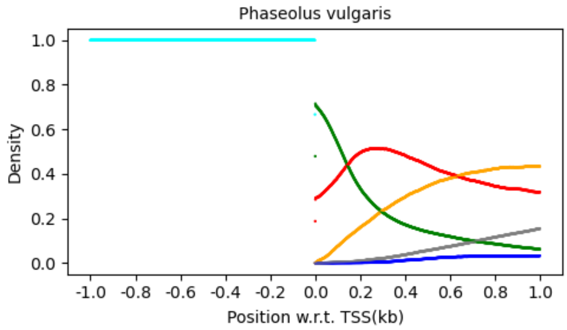
Phaseolus vulgaris
Density
Position w.r.t. TSS(kb)

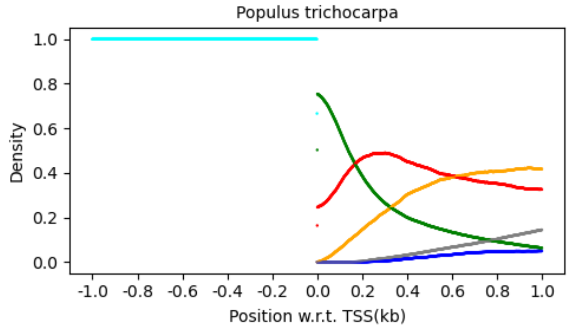
Populus trichocarpa
Density
Position w.r.t. TSS(kb)

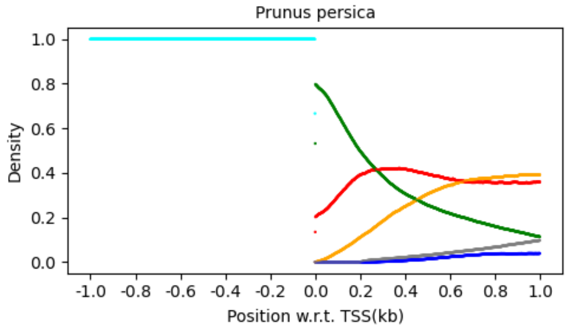
Prunus persica
Density
Position w.r.t. TSS(kb)

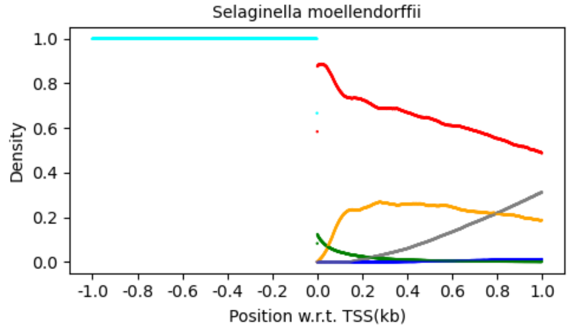
Selaginella moellendorffii
Density
Position w.r.t. TSS(kb)

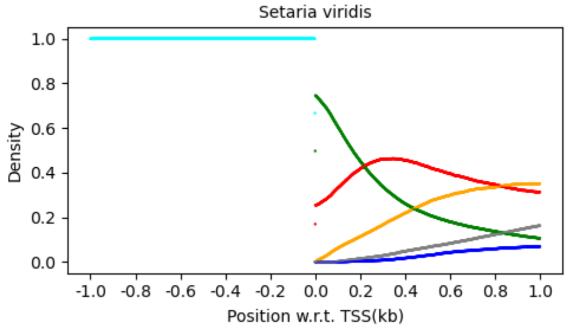
Setaria viridis
Density
Position w.r.t. TSS(kb)

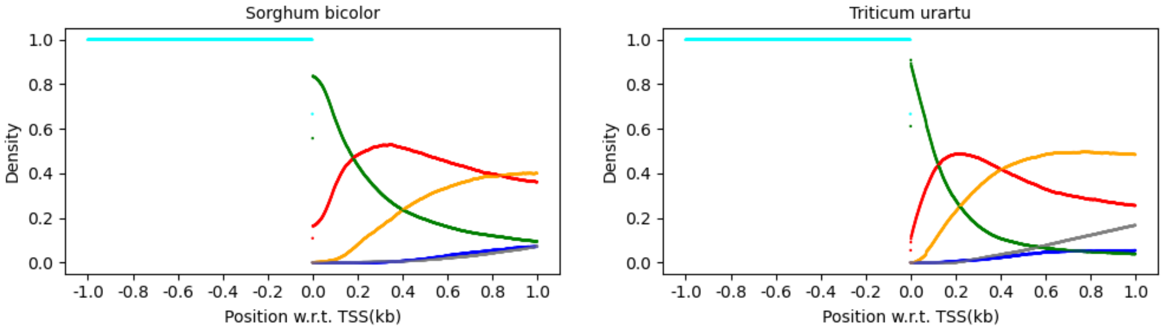
Sorghum bicolor
Triticum urartu
Density
Position w.r.t. TSS(kb)
1.0
0.8
0.6
0.4
0.2
0.0
-1.0
-0.8
-0.6
-0.4
-0.2
0.0
0.2
0.4
0.6
0.8
1.0

Figure S2

Genome-wide GC signal profiles around TSS of selected vertebrates, plants, *C. elegans* (worm), *D. melanogaster* (fly) and *S. cerevisiae* (yeast). The leftmost panel figures show the contributions of the between-region signals (BR) and the within-region signals (WR) to the full GC signals (FS). Middle figures display the transcript constituents of the between-region signal. The dashed lines in the middle figures represent the average GC content values of the various regions, and the dots of a specific color represents the between-region GC content per base pair of the corresponding region. Right-most figures show the transcript constituents of the within-region signal. Middle and right-most plots show only the region downstream of TSS.

Vertebrates

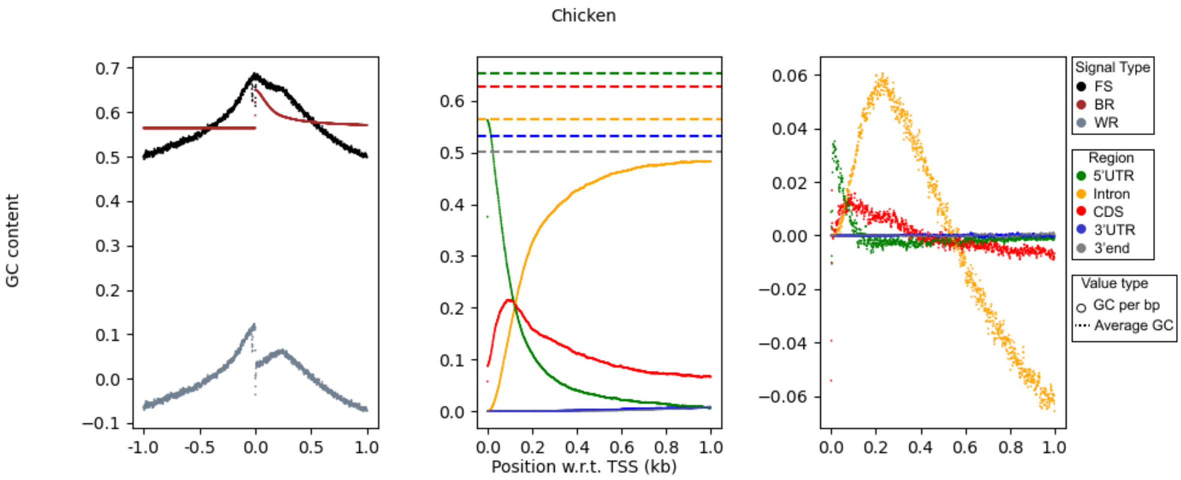

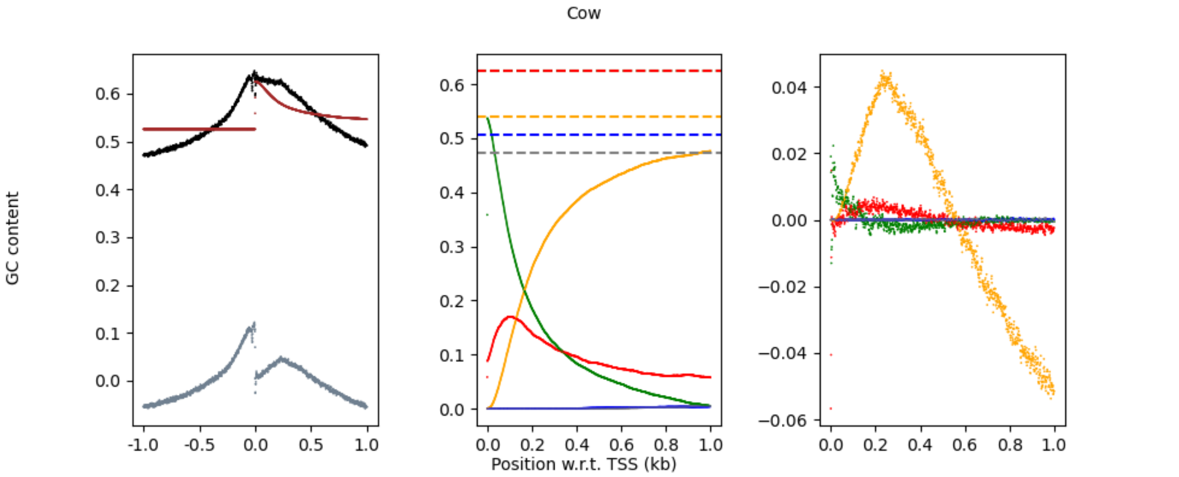

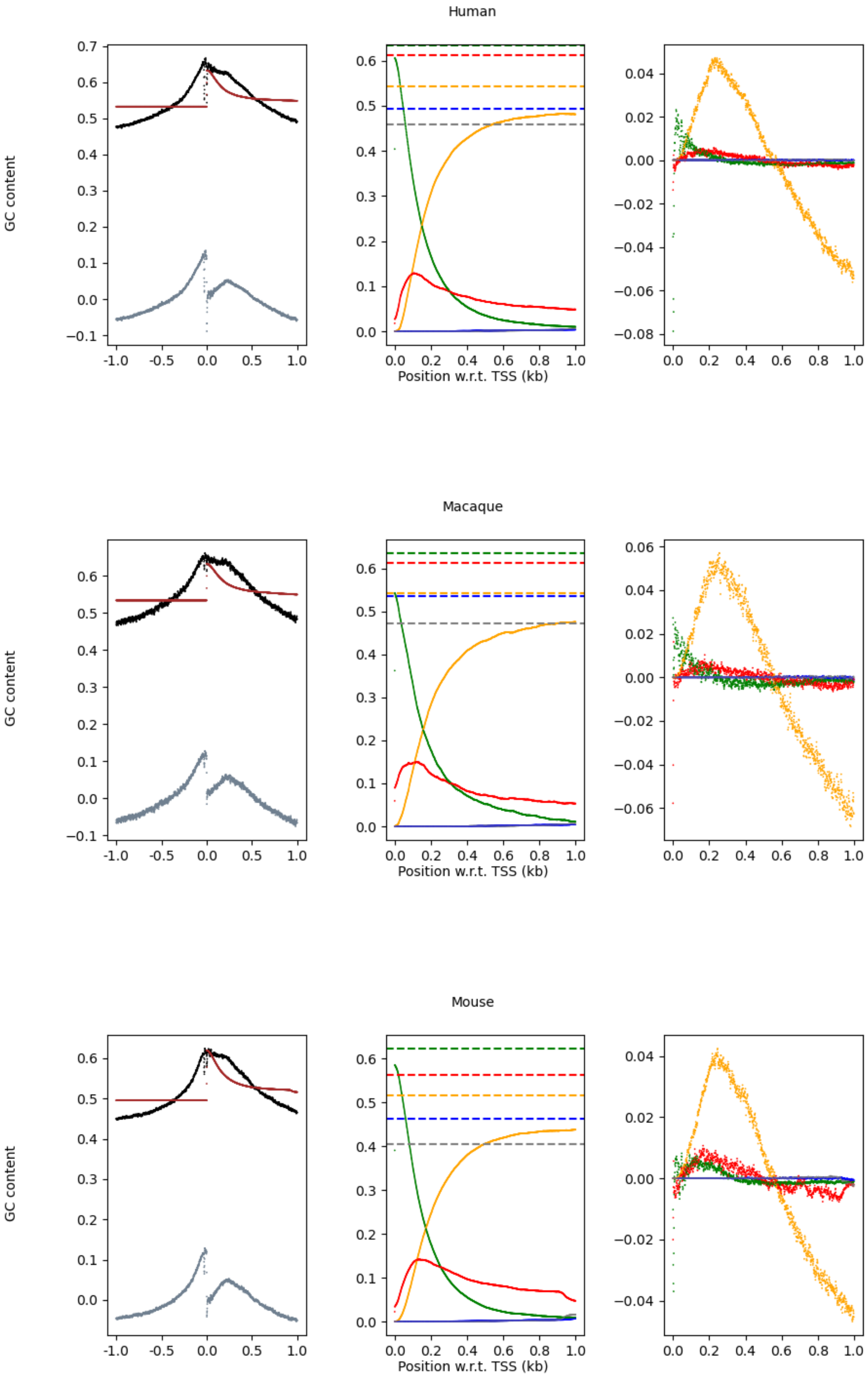
Human
Macaque
Mouse
GC content
Position w.r.t. TSS (kb)

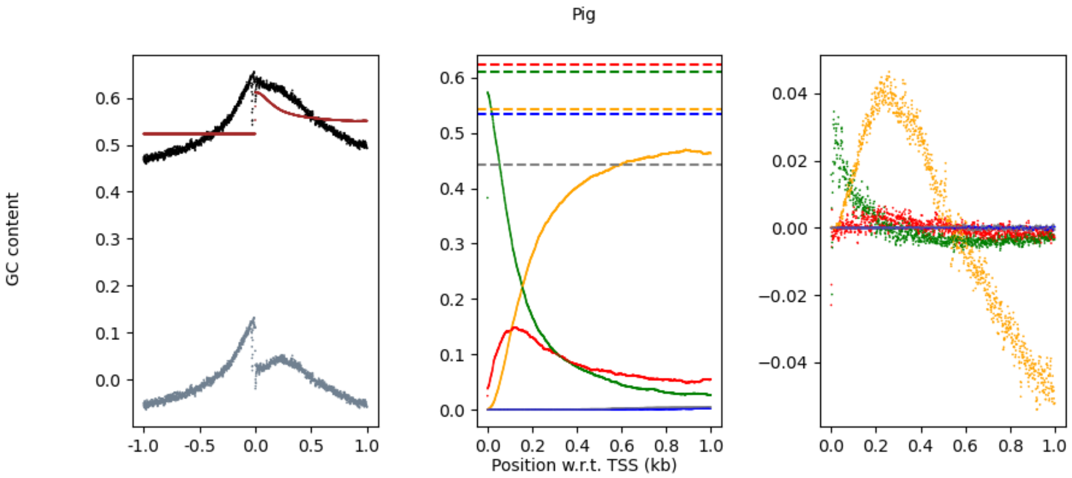
Pig
GC content
Position w.r.t. TSS (kb)

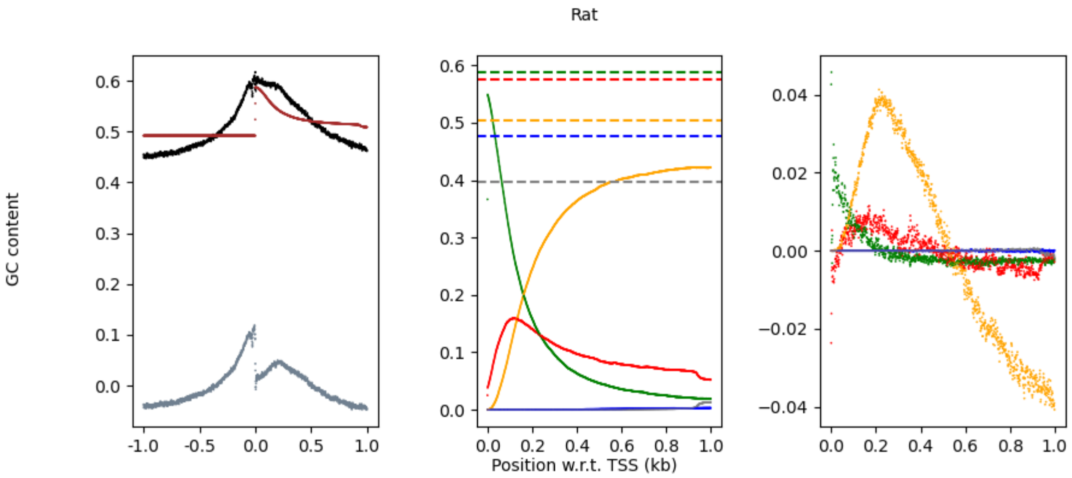
Rat
GC content
Position w.r.t. TSS (kb)

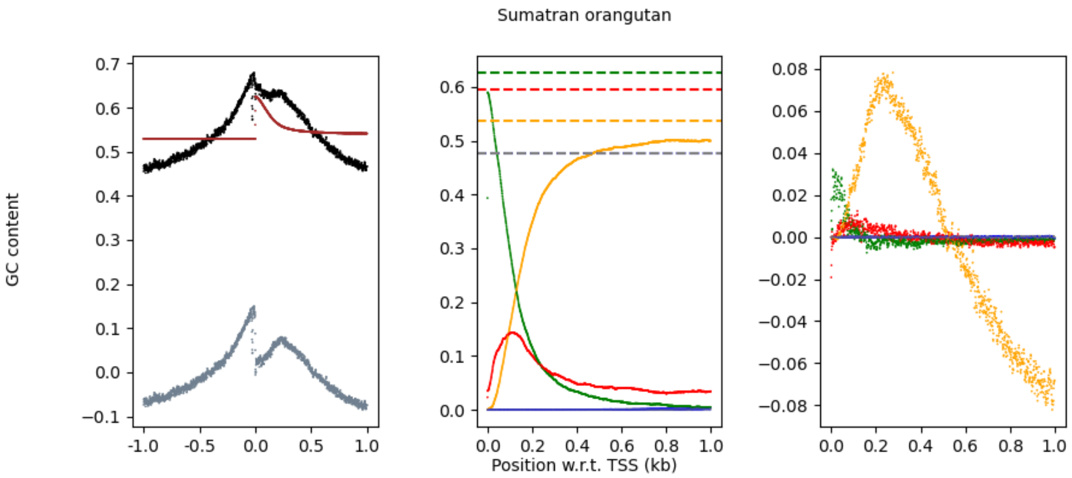
Sumatran orangutan
GC content
Position w.r.t. TSS (kb)

Tropical clawed frog

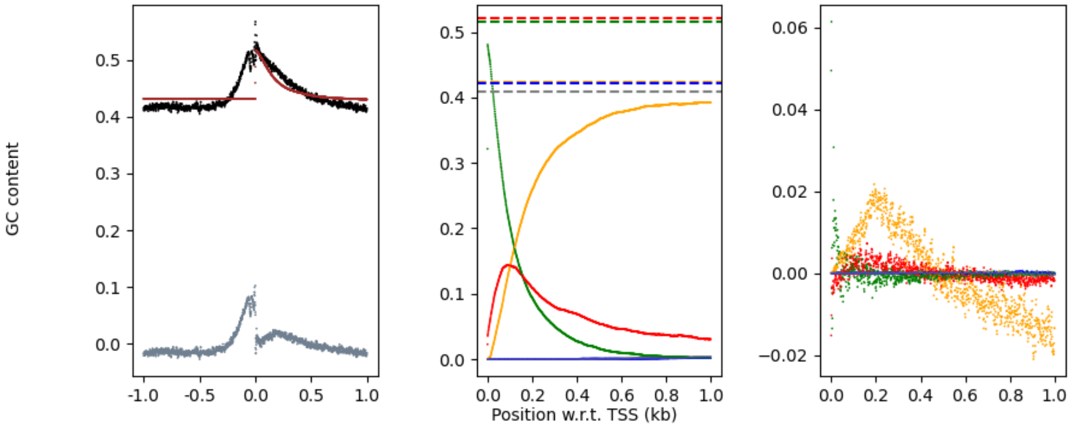

Zebrafish

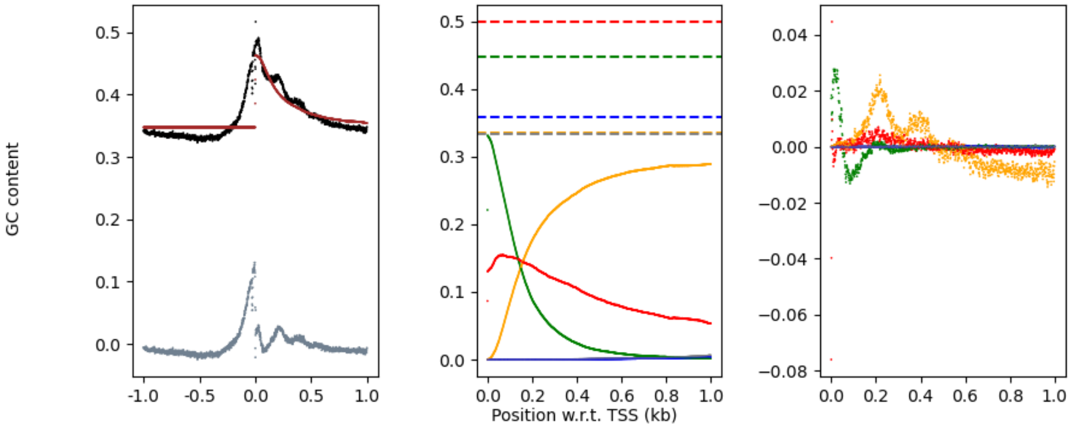

Plants

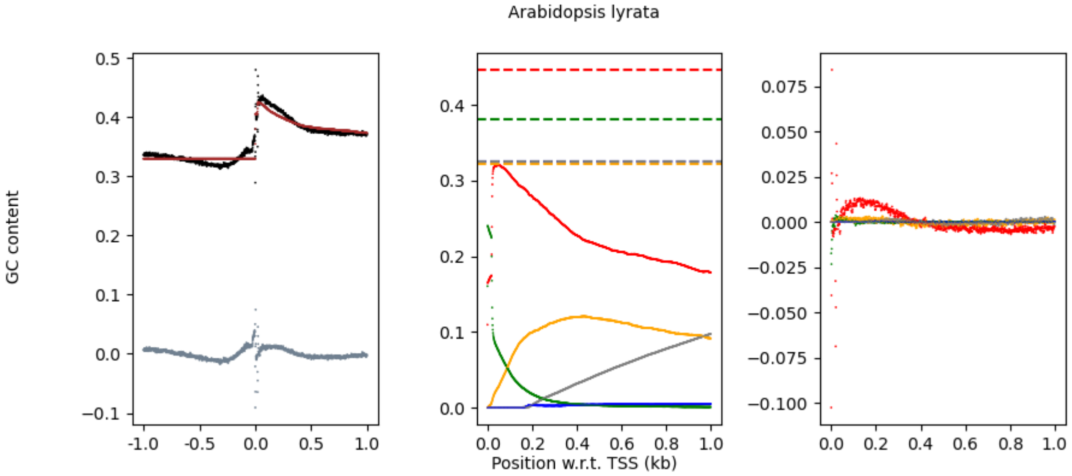

Arabidopsis lyrata
GC content
Position w.r.t. TSS (kb)

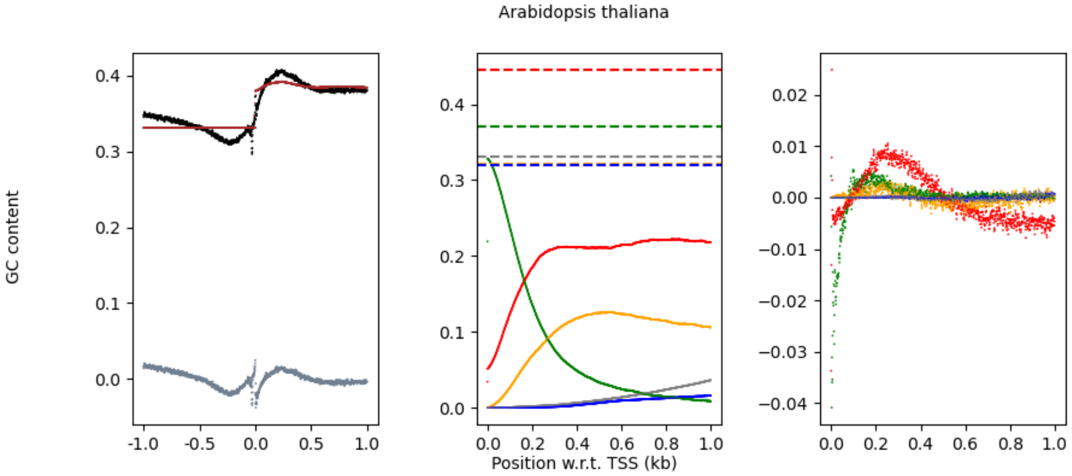

Arabidopsis thaliana
GC content
Position w.r.t. TSS (kb)

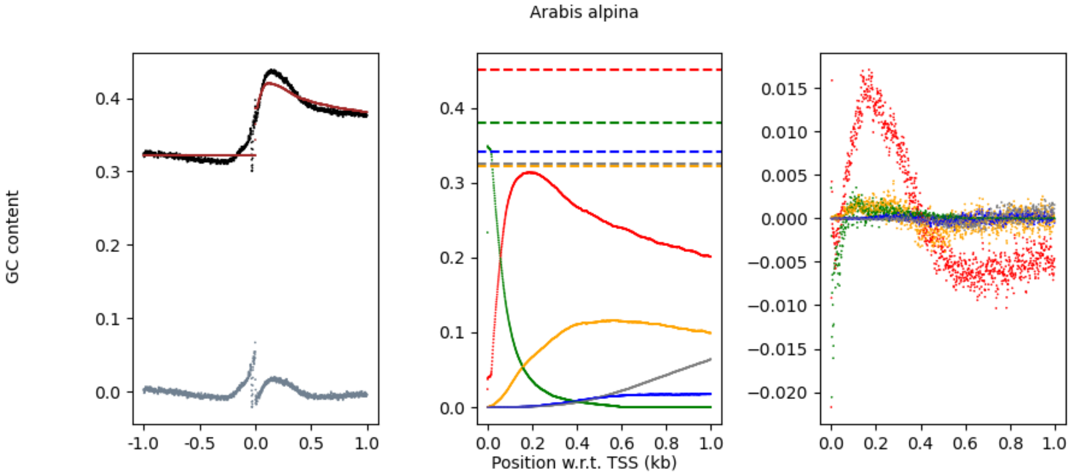

Arabis alpina
GC content
Position w.r.t. TSS (kb)

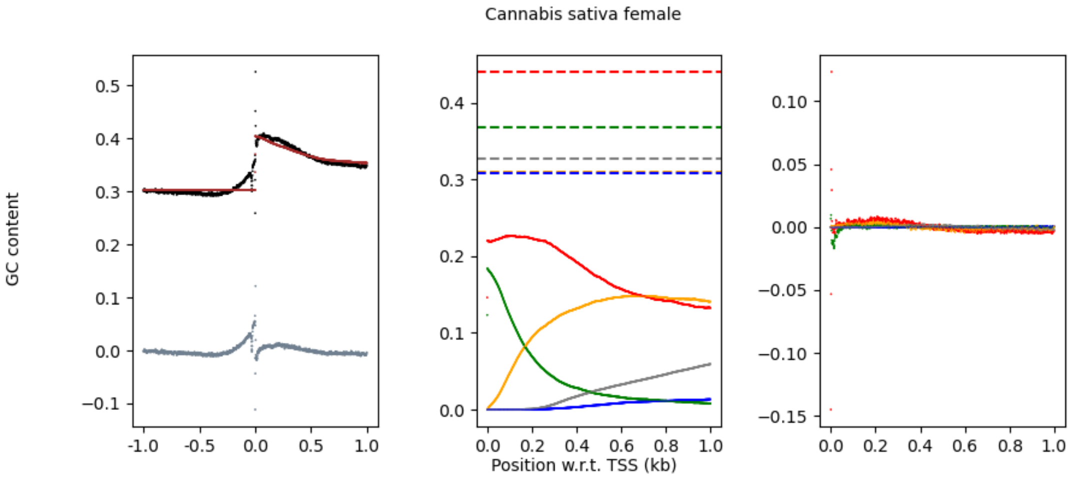
Cannabis sativa female
GC content
0.5
0.4
0.3
0.2
0.1
0.0
−0.1
-1.0 -0.5 0.0 0.5 1.0
0.4
0.3
0.2
0.1
0.0
0.0 0.2 0.4 0.6 0.8 1.0
Position w.r.t. TSS (kb)
0.10
0.05
0.00
−0.05
−0.10
−0.15
0.0 0.2 0.4 0.6 0.8 1.0

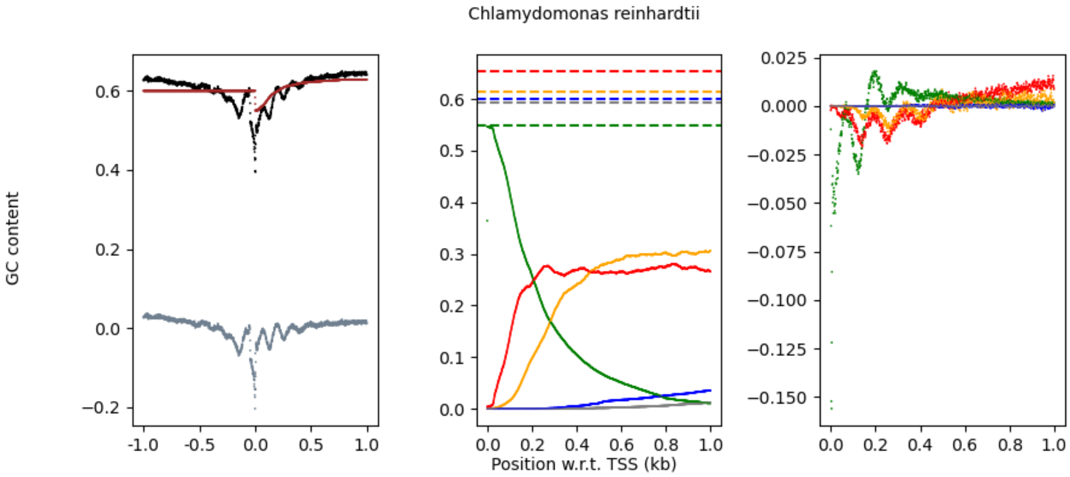
Chlamydomonas reinhardtii
GC content
0.6
0.4
0.2
0.0
−0.2
-1.0 -0.5 0.0 0.5 1.0
0.6
0.5
0.4
0.3
0.2
0.1
0.0
0.0 0.2 0.4 0.6 0.8 1.0
Position w.r.t. TSS (kb)
0.025
0.000
−0.025
−0.050
−0.075
−0.100
−0.125
−0.150
0.0 0.2 0.4 0.6 0.8 1.0

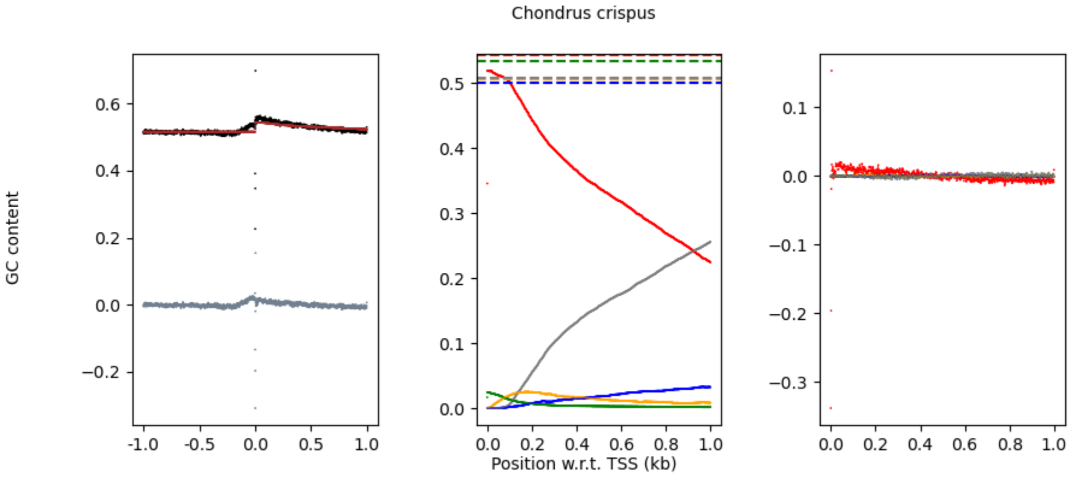
Chondrus crispus
GC content
0.6
0.4
0.2
0.0
−0.2
-1.0 -0.5 0.0 0.5 1.0
0.5
0.4
0.3
0.2
0.1
0.0
0.0 0.2 0.4 0.6 0.8 1.0
Position w.r.t. TSS (kb)
0.1
0.0
−0.1
−0.2
−0.3
0.0 0.2 0.4 0.6 0.8 1.0

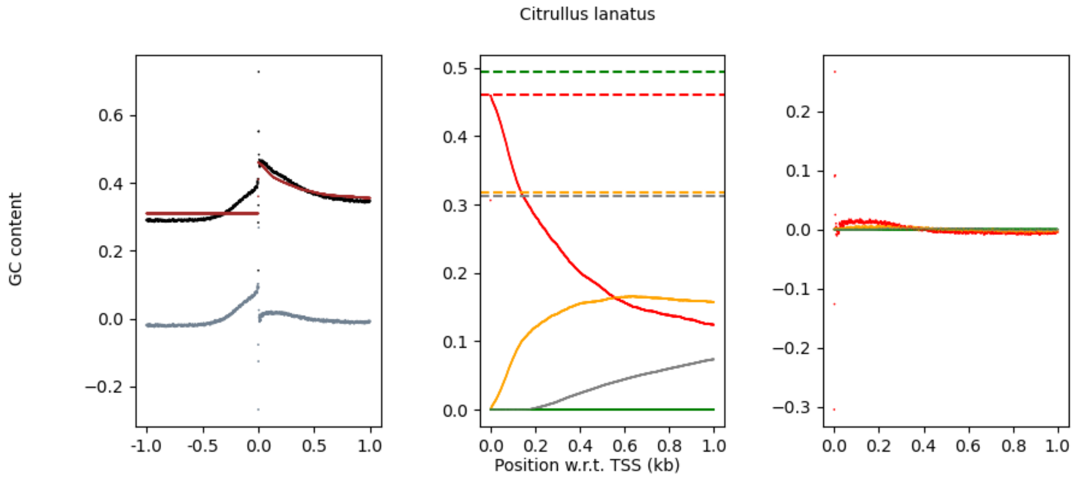
Citrullus lanatus
GC content
Position w.r.t. TSS (kb)

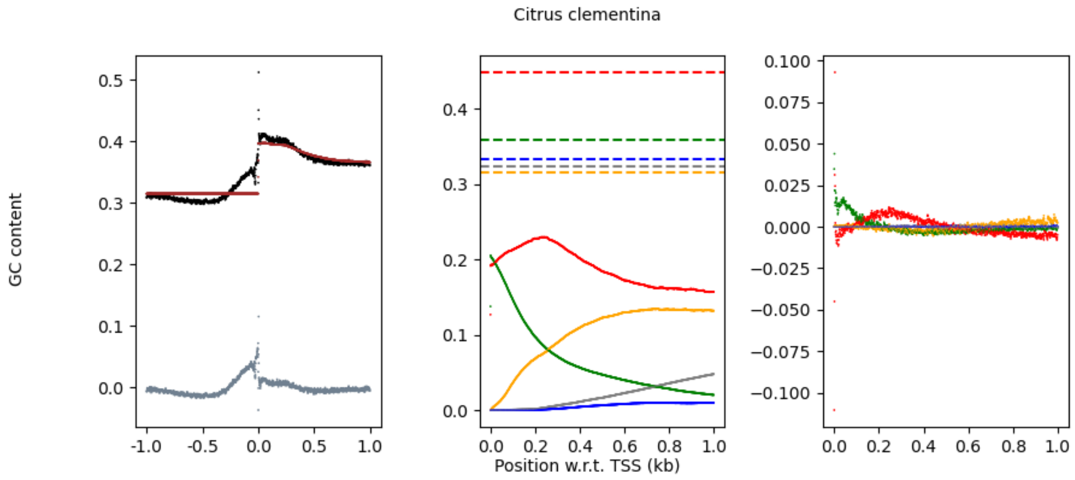
Citrus clementina
GC content
Position w.r.t. TSS (kb)

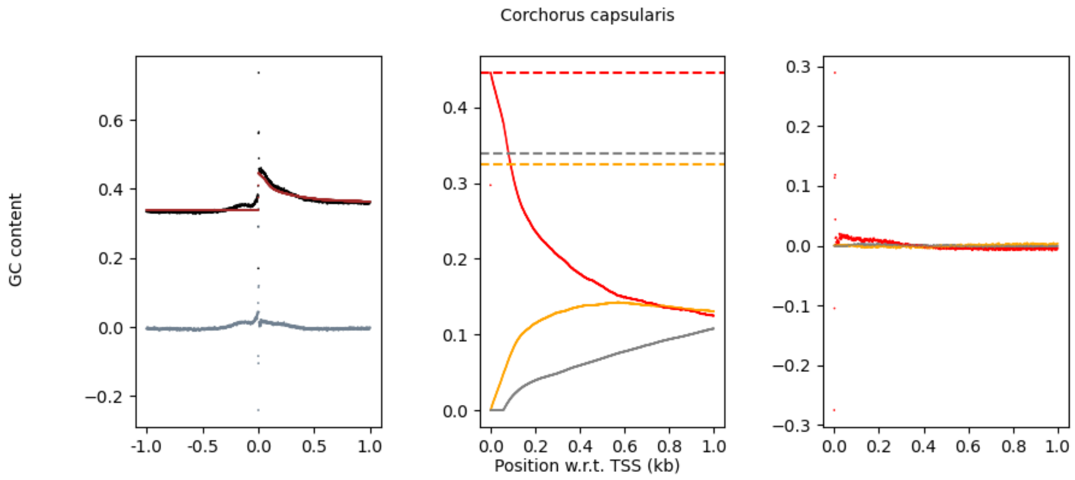
Corchorus capsularis
GC content
Position w.r.t. TSS (kb)

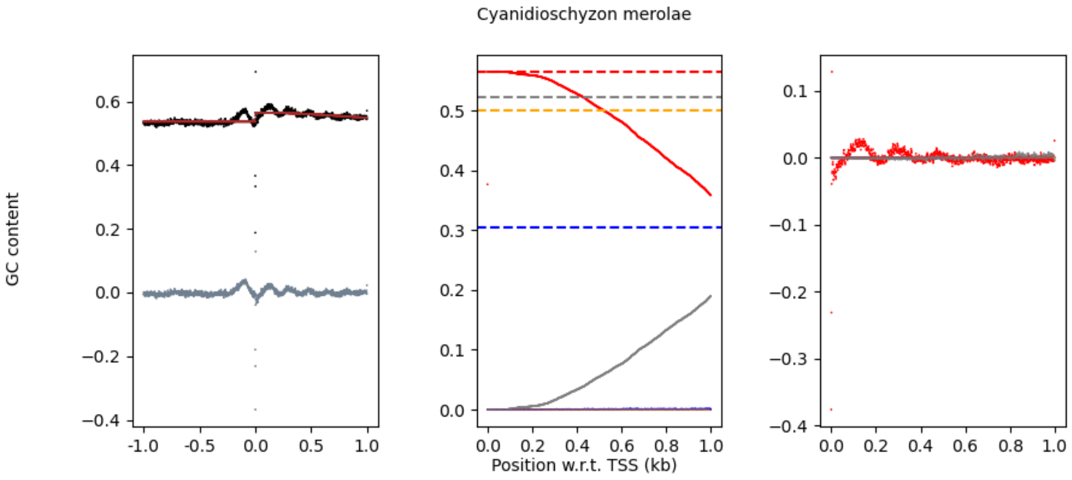
Cyanidioschyzon merolae
GC content
Position w.r.t. TSS (kb)

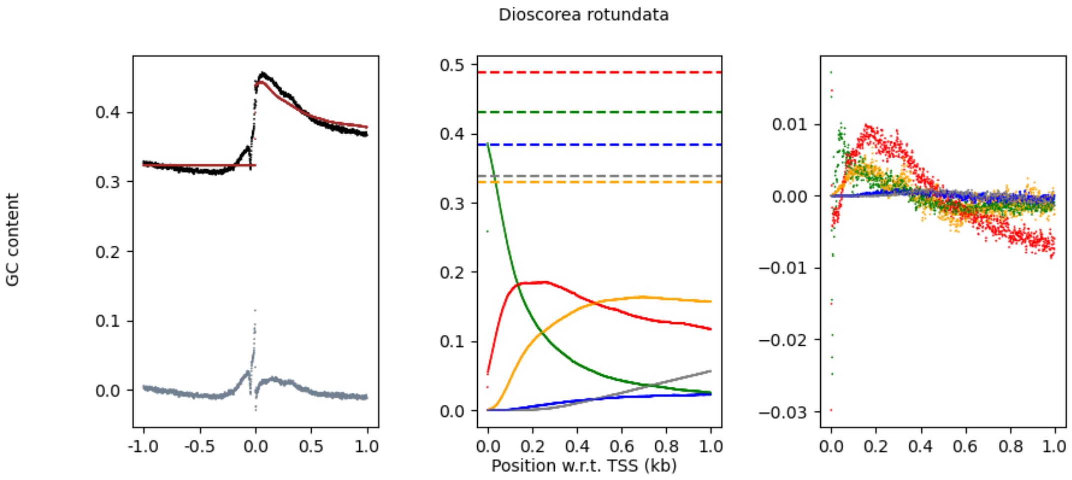
Dioscorea rotundata
GC content
Position w.r.t. TSS (kb)

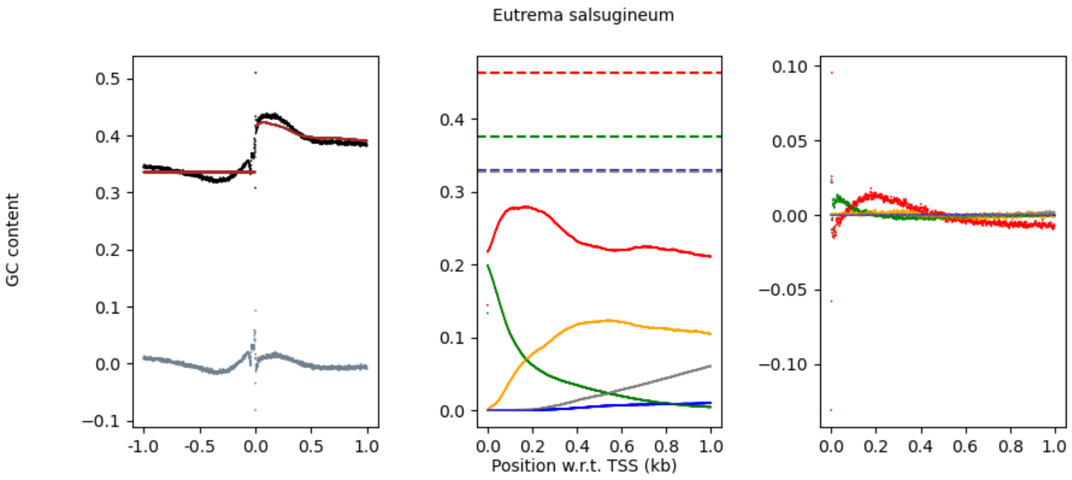
Eutrema salsugineum
GC content
Position w.r.t. TSS (kb)

Galdieria sulphuraria

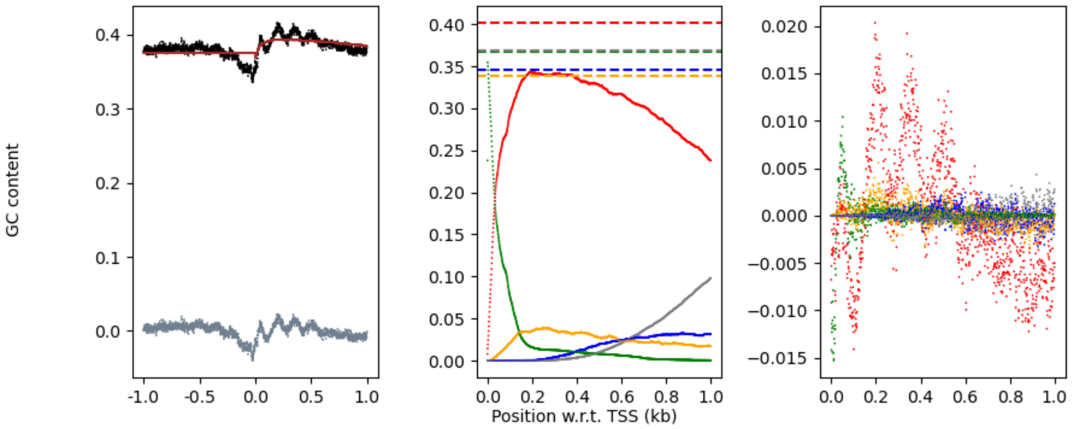

Helianthus annuus

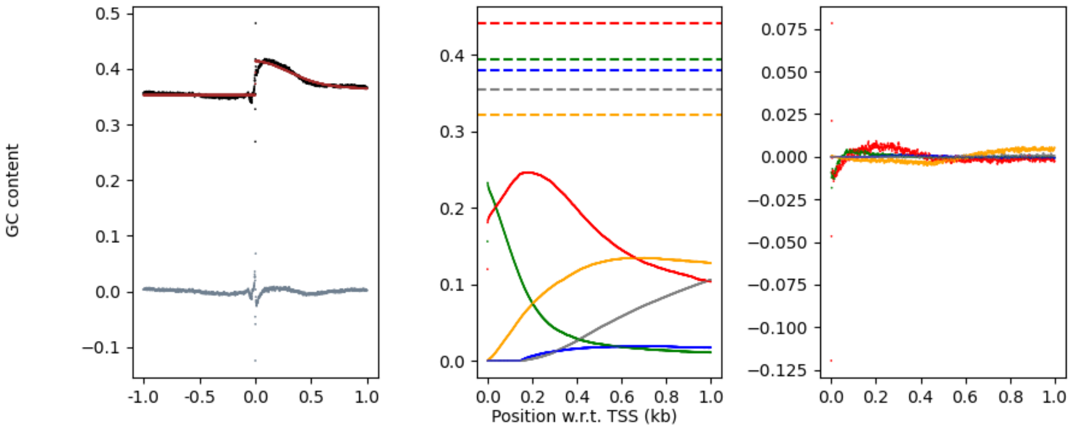

Hordeum vulgare TRITEX

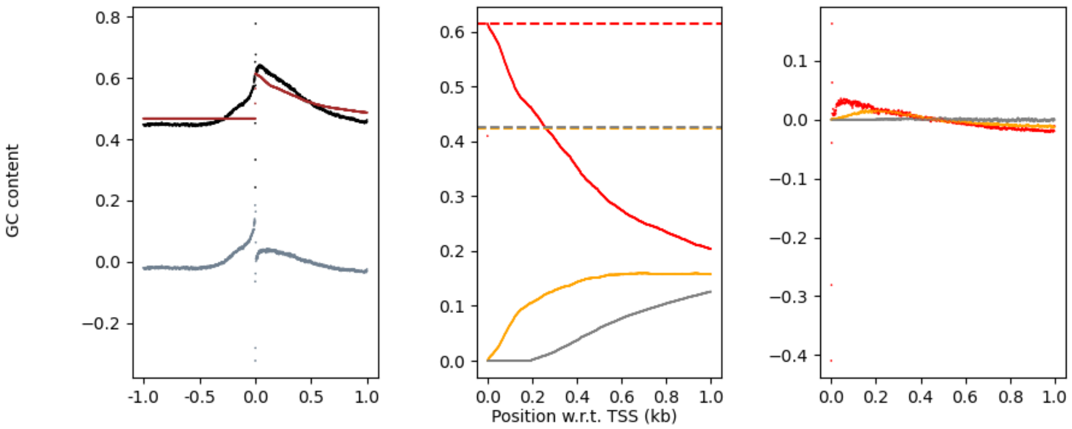

Hordeum vulgare

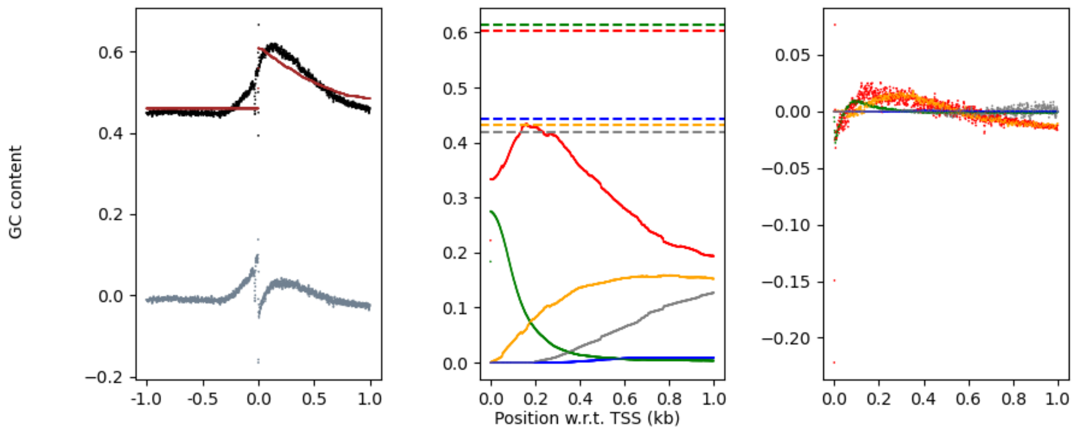

Lactuca sativa

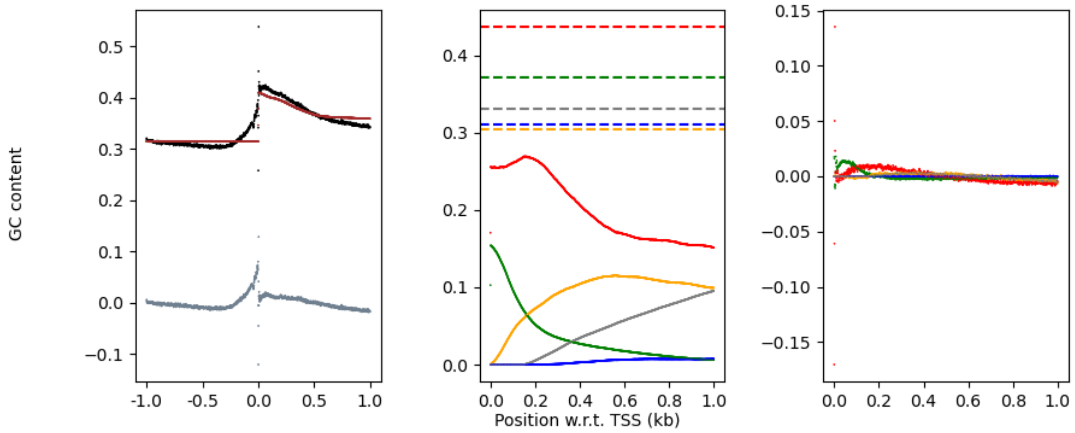

Medicago truncatula

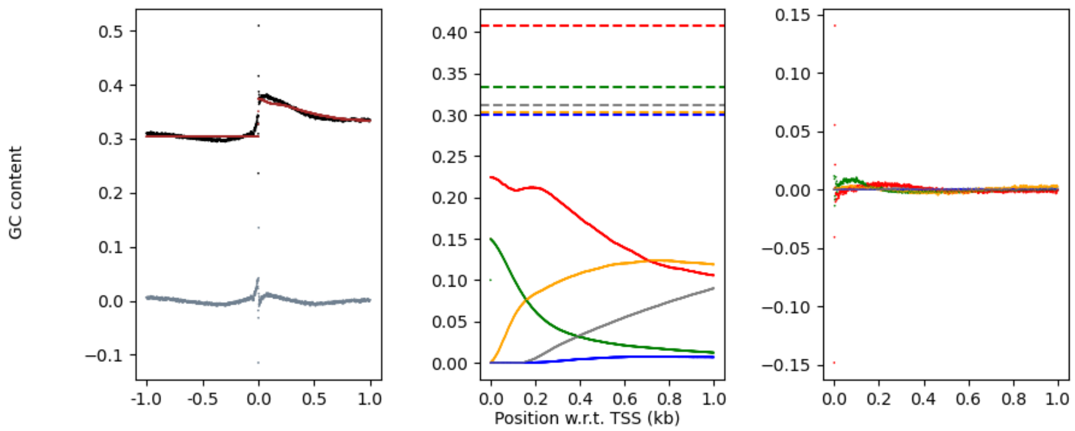

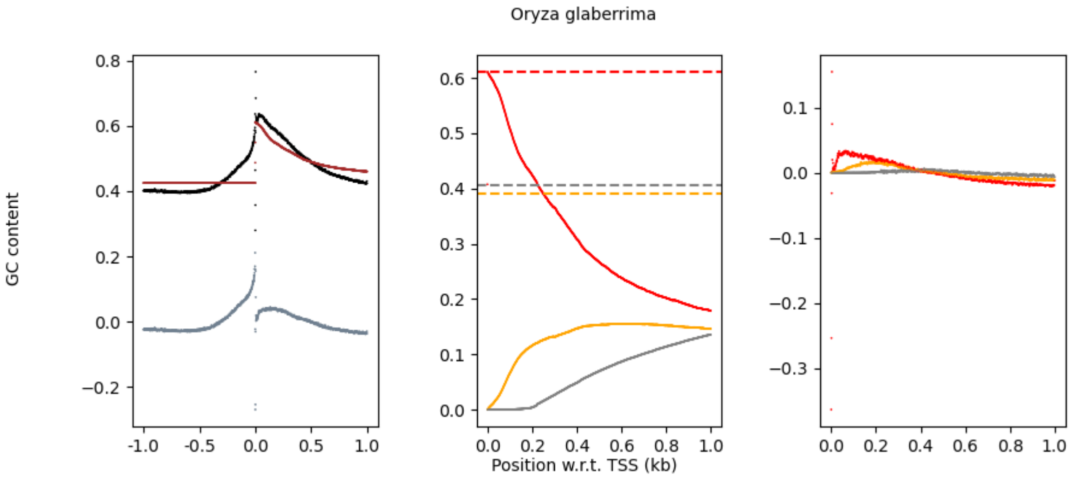
Oryza glaberrima
GC content
Position w.r.t. TSS (kb)

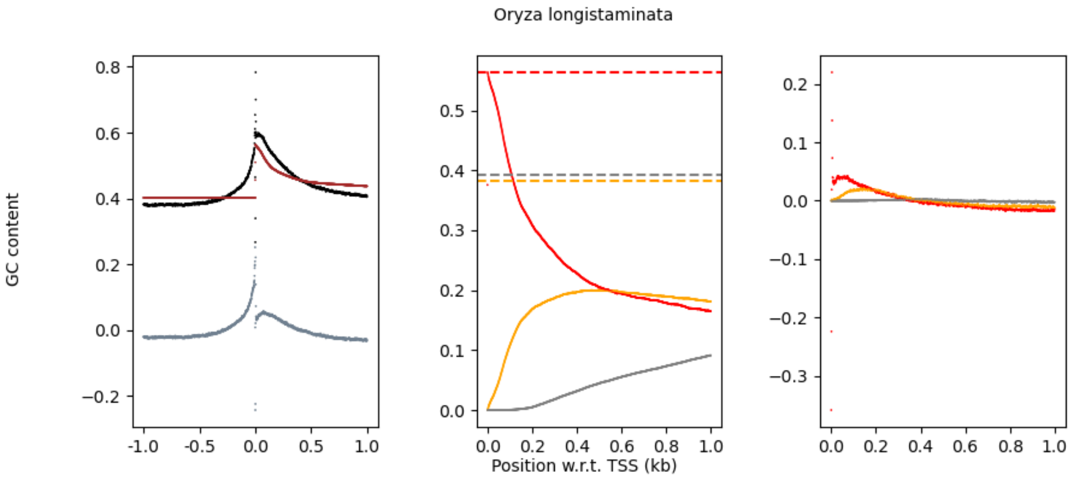
Oryza longistaminata
GC content
Position w.r.t. TSS (kb)

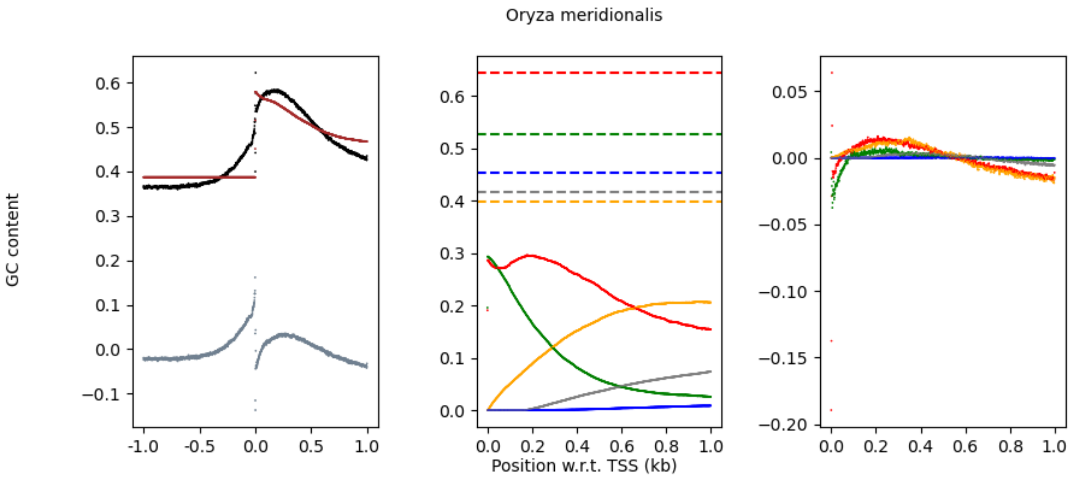
Oryza meridionalis
GC content
Position w.r.t. TSS (kb)

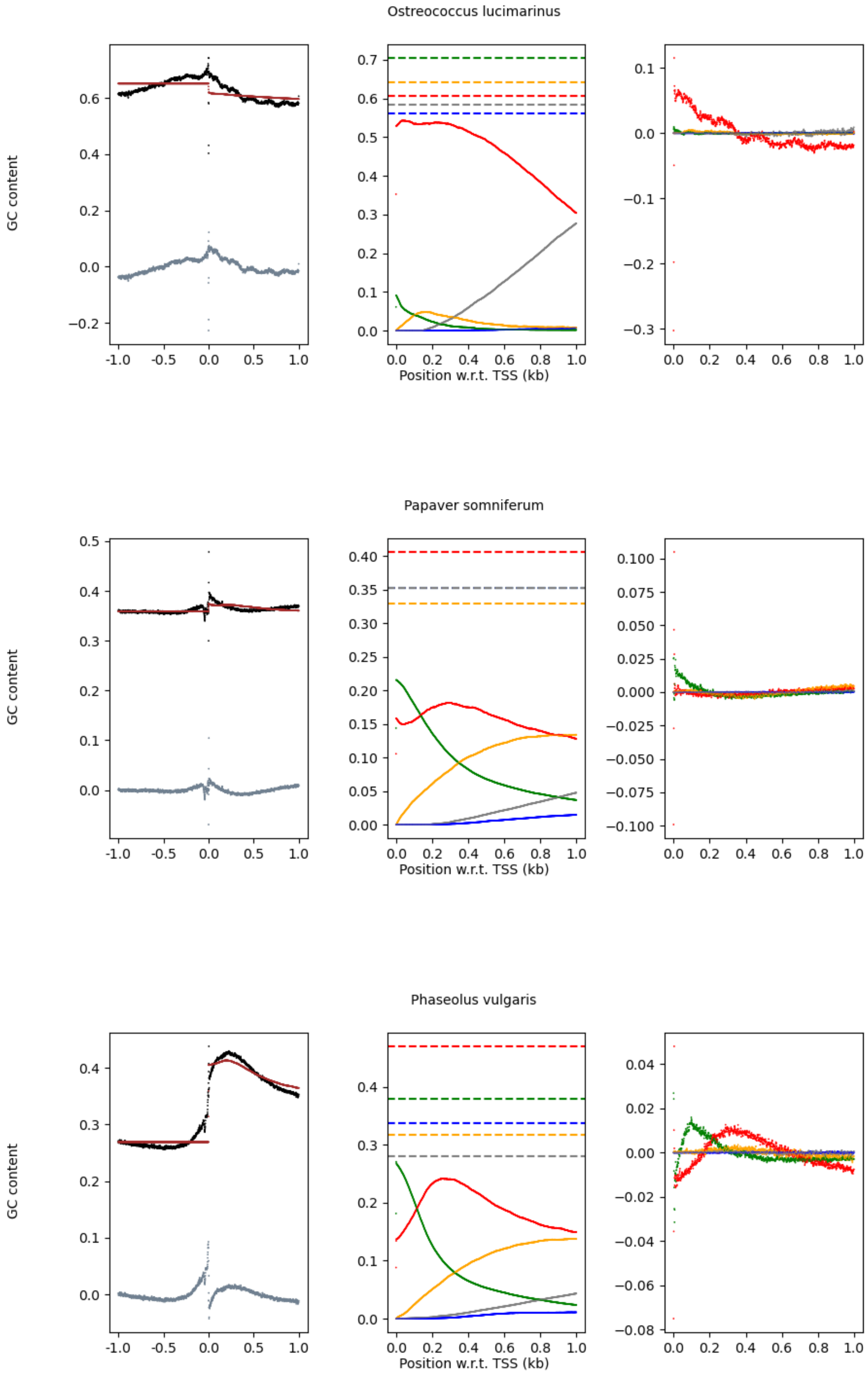

Ostreococcus lucimarinus
GC content
Position w.r.t. TSS (kb)
Papaver somniferum
GC content
Position w.r.t. TSS (kb)
Phaseolus vulgaris
GC content
Position w.r.t. TSS (kb)

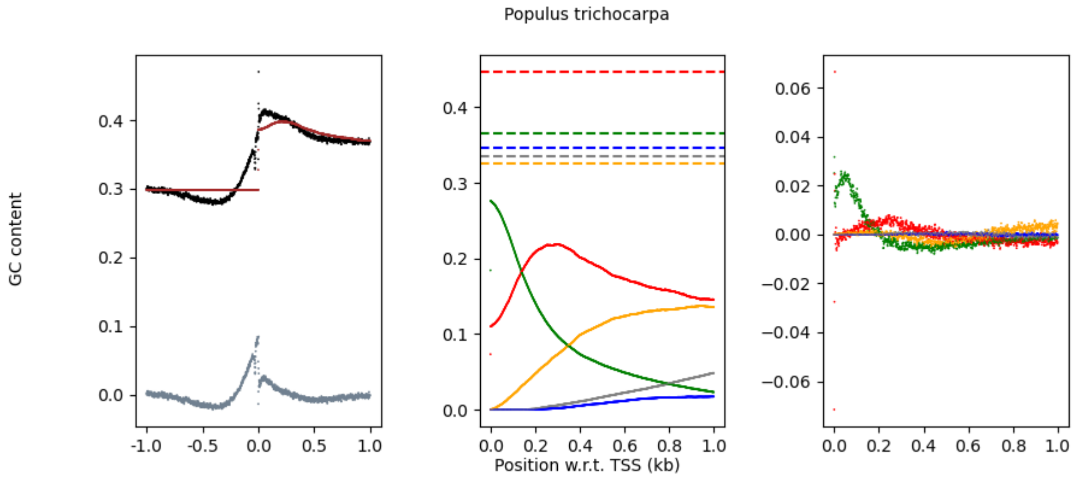
Populus trichocarpa
GC content
Position w.r.t. TSS (kb)

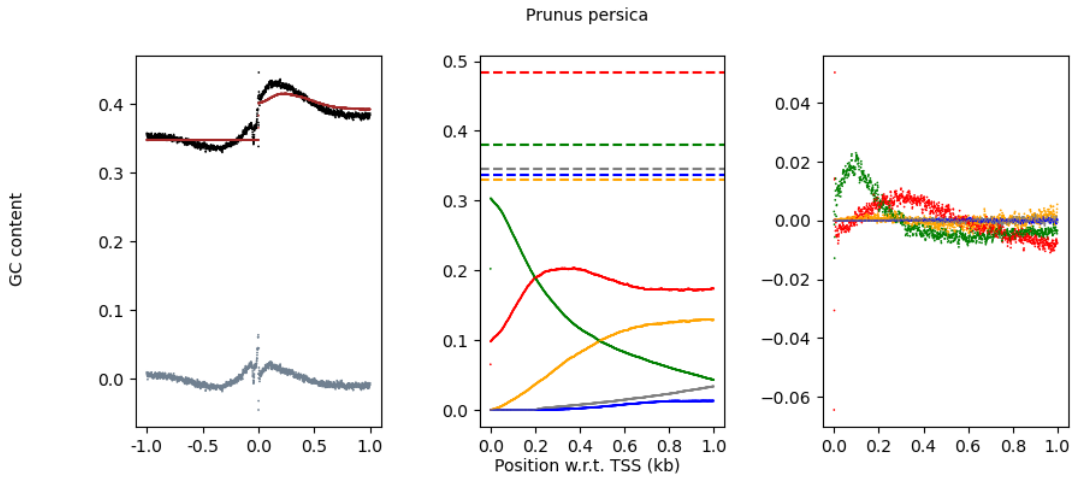
Prunus persica
GC content
Position w.r.t. TSS (kb)

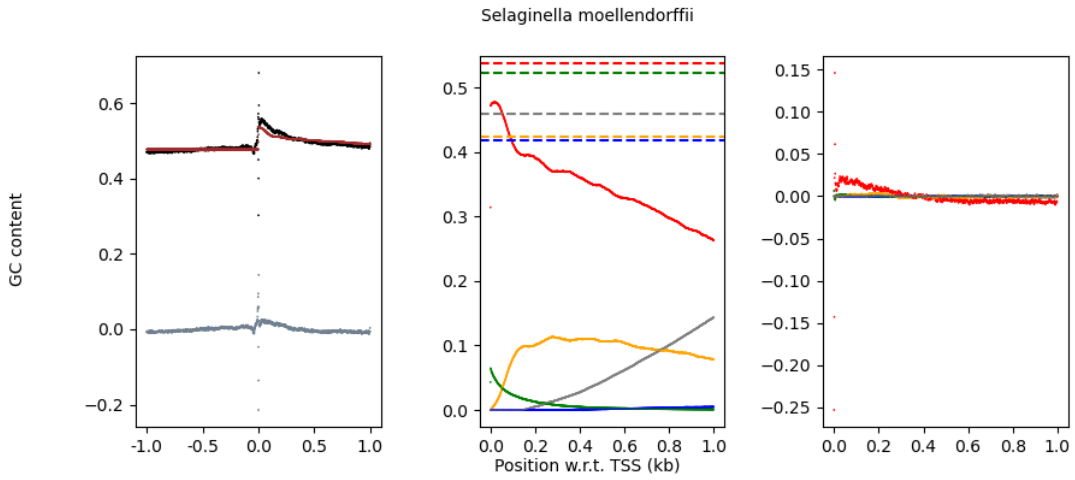
Selaginella moellendorffii
GC content
Position w.r.t. TSS (kb)

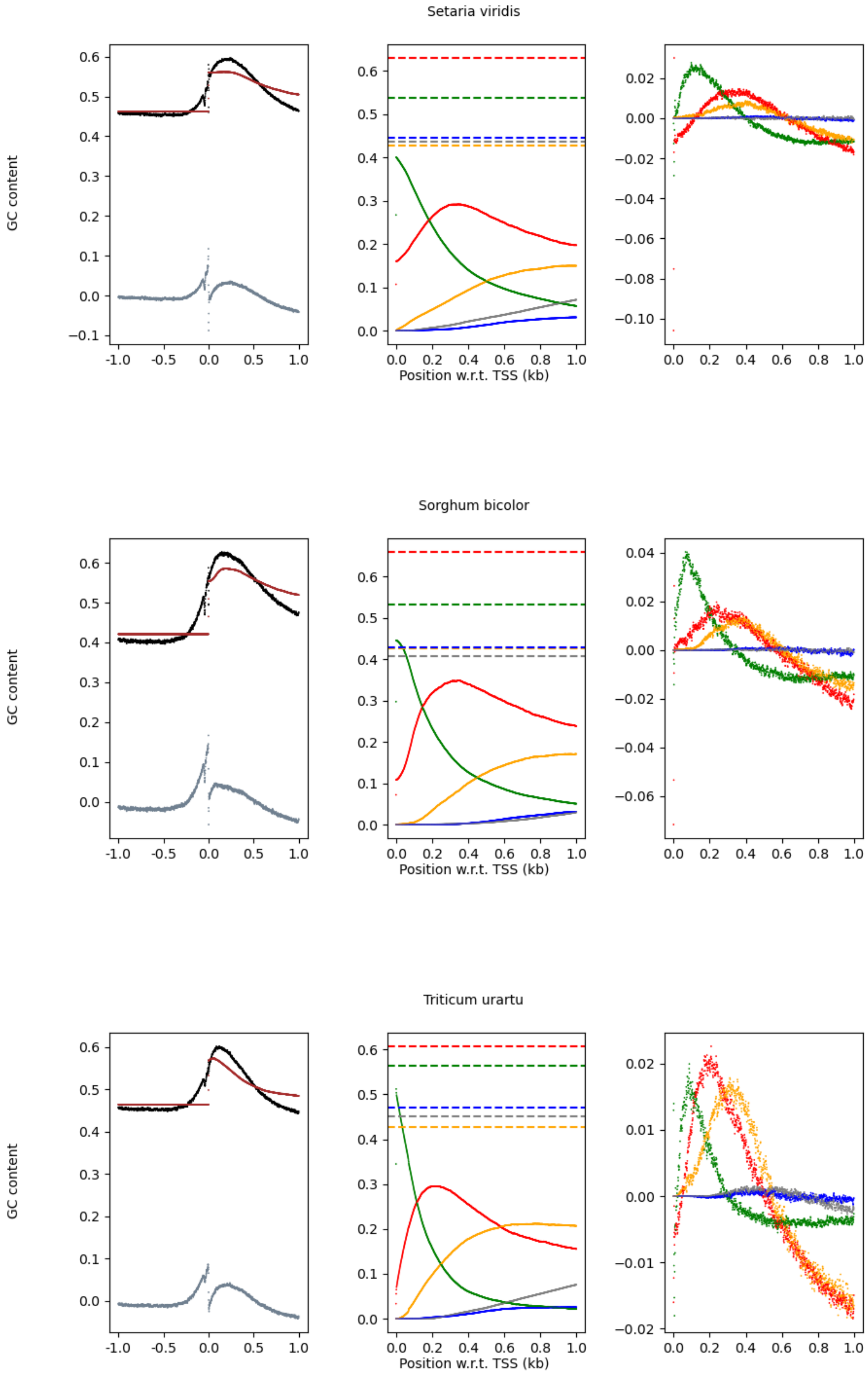
Setaria viridis
GC content
Position w.r.t. TSS (kb)
Sorghum bicolor
GC content
Position w.r.t. TSS (kb)
Triticum urartu
GC content
Position w.r.t. TSS (kb)

Others

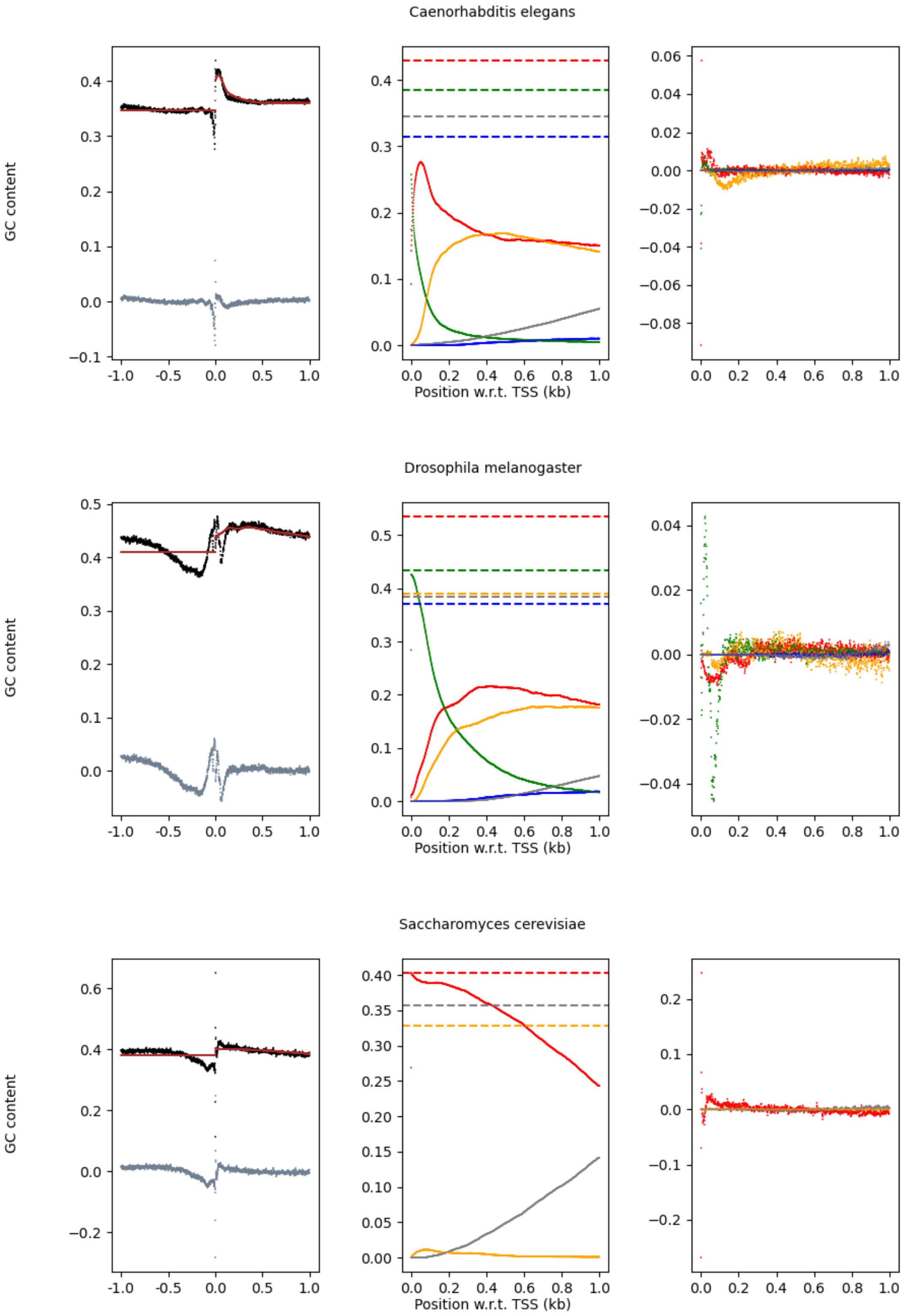

Figure S3

The bar chart (left figure) shows the average GC content of transcriptional regions and the scatterplot (right figure) shows the normalized within-region signal for selected vertebrates and plants (see Methods for details).

For the plots of the normalized within-region signal, data points with Z-scores greater than 5 were excluded.

Vertebrates

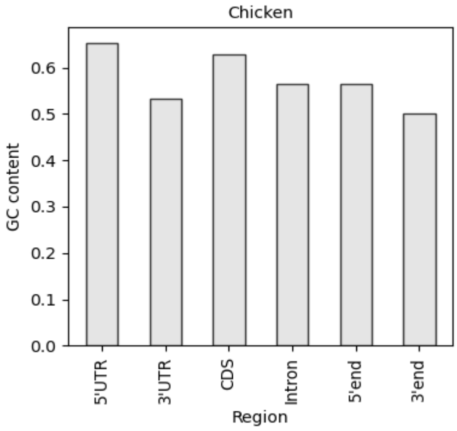

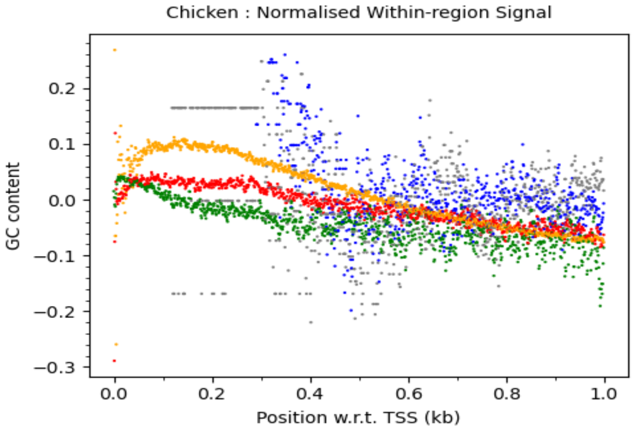

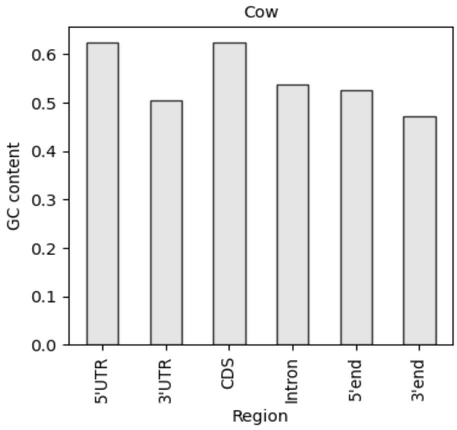

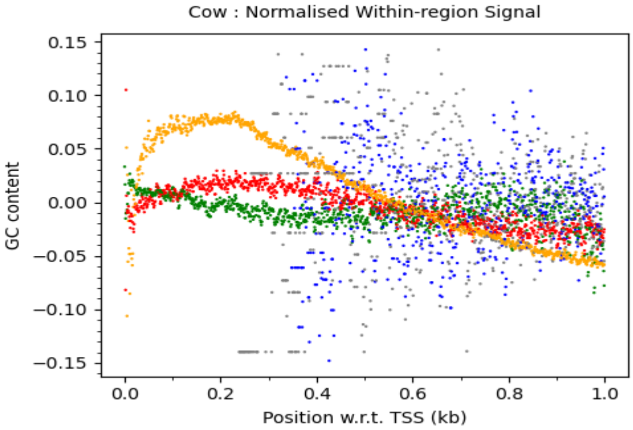

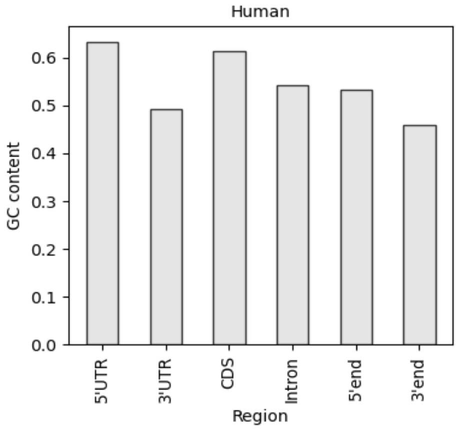

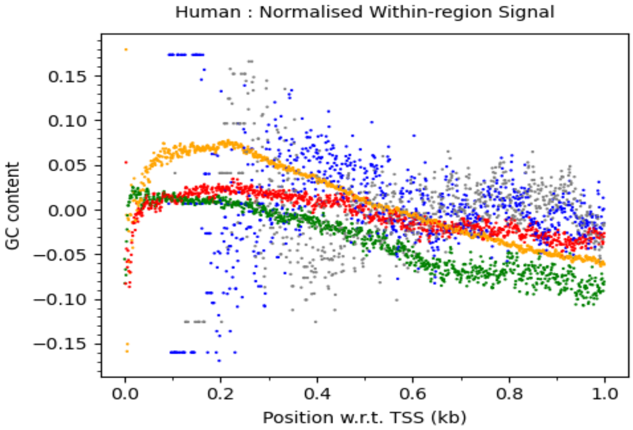

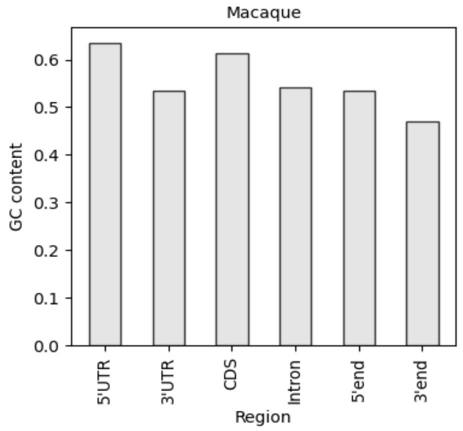
Macaque
GC content
0.0
0.1
0.2
0.3
0.4
0.5
0.6
5'UTR
3'UTR
CDS
Intron
5'end
3'end
Region

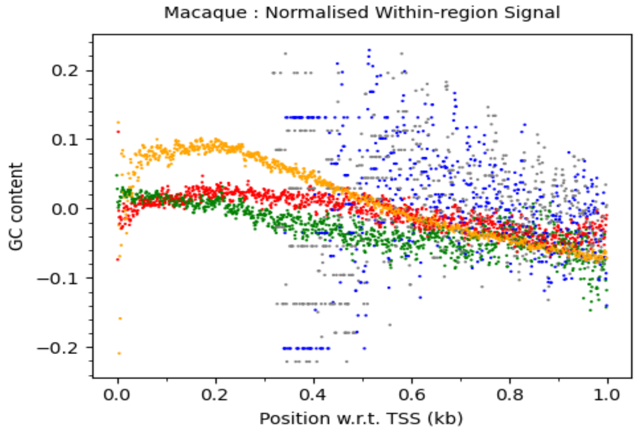
Macaque : Normalised Within-region Signal
GC content
−0.2
−0.1
0.0
0.1
0.2
0.0
0.2
0.4
0.6
0.8
1.0
Position w.r.t. TSS (kb)

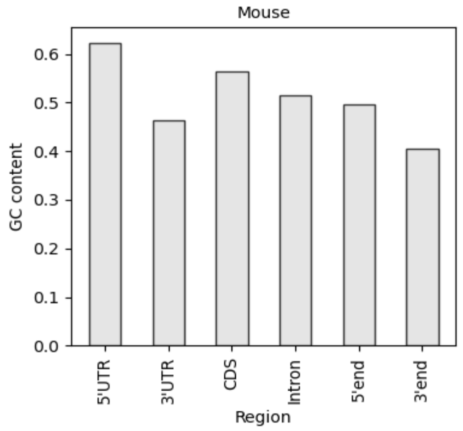
Mouse
GC content
0.0
0.1
0.2
0.3
0.4
0.5
0.6
5'UTR
3'UTR
CDS
Intron
5'end
3'end
Region

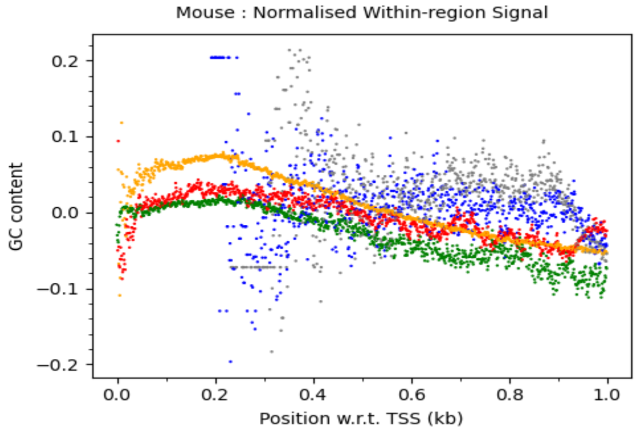
Mouse : Normalised Within-region Signal
GC content
−0.2
−0.1
0.0
0.1
0.2
0.0
0.2
0.4
0.6
0.8
1.0
Position w.r.t. TSS (kb)

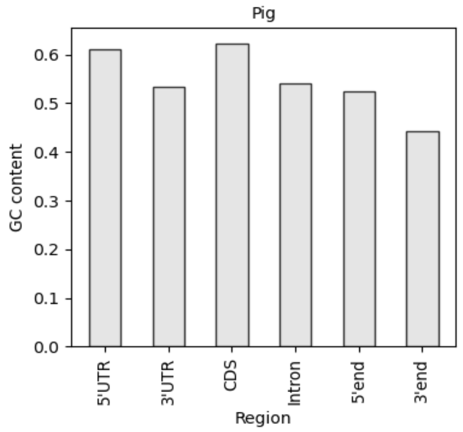
Pig
GC content
0.0
0.1
0.2
0.3
0.4
0.5
0.6
5'UTR
3'UTR
CDS
Intron
5'end
3'end
Region

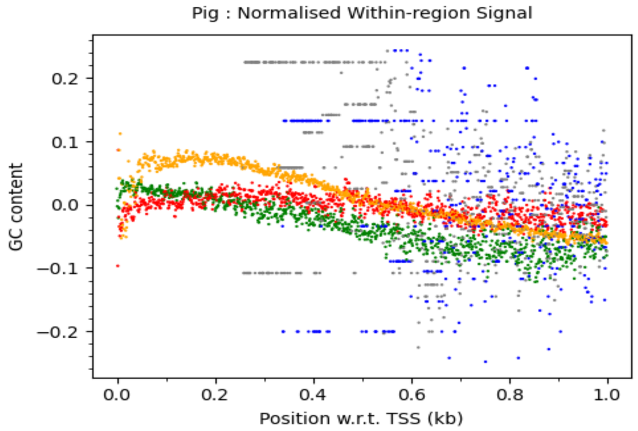
Pig : Normalised Within-region Signal
GC content
−0.2
−0.1
0.0
0.1
0.2
0.0
0.2
0.4
0.6
0.8
1.0
Position w.r.t. TSS (kb)

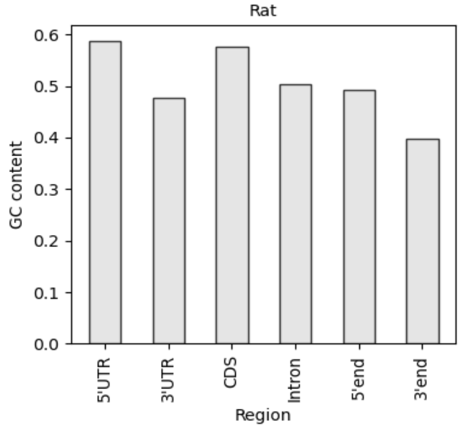
Rat
GC content
Region
5'UTR
3'UTR
CDS
Intron
5'end
3'end

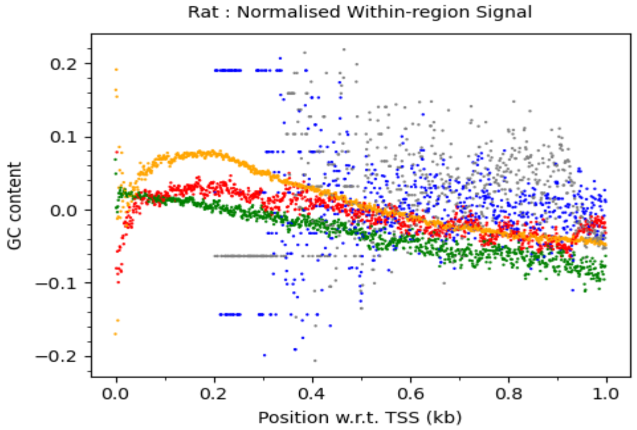
Rat : Normalised Within-region Signal
GC content
Position w.r.t. TSS (kb)

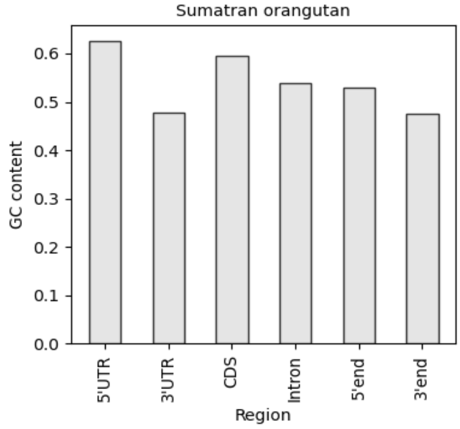
Sumatran orangutan
GC content
Region
5'UTR
3'UTR
CDS
Intron
5'end
3'end

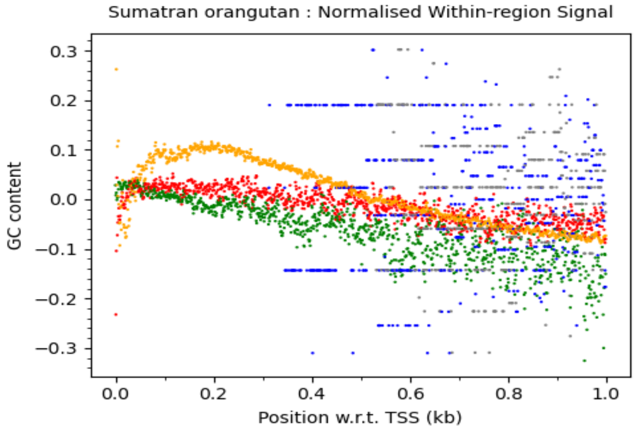
Sumatran orangutan : Normalised Within-region Signal
GC content
Position w.r.t. TSS (kb)

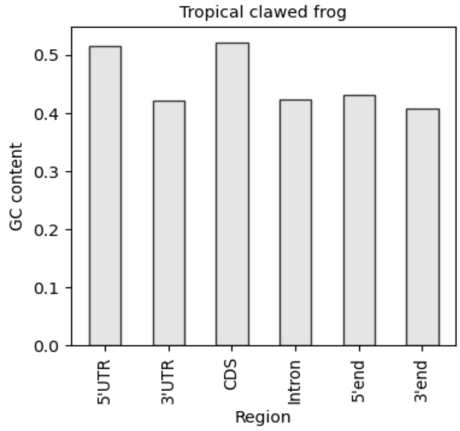
Tropical clawed frog
GC content
Region
5'UTR
3'UTR
CDS
Intron
5'end
3'end

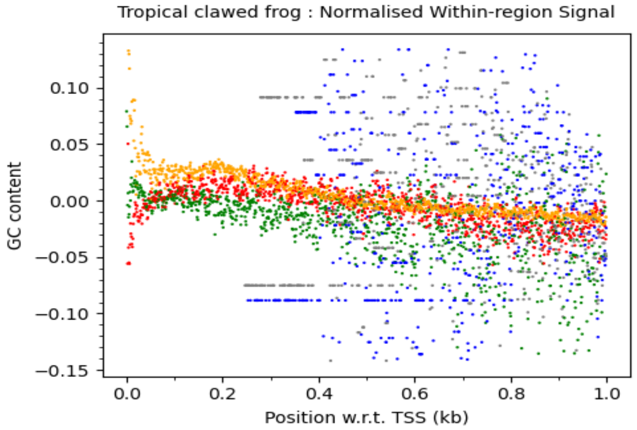
Tropical clawed frog : Normalised Within-region Signal
GC content
Position w.r.t. TSS (kb)

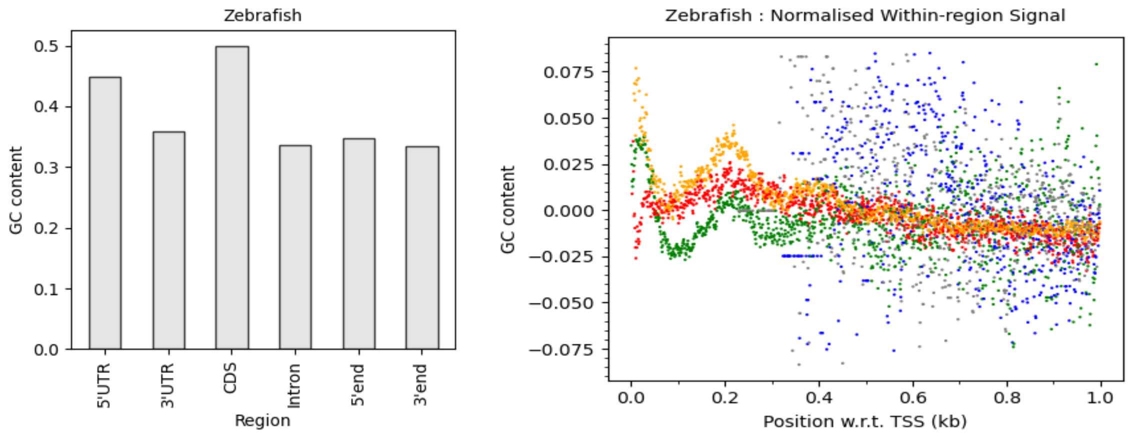
Zebrafish
GC content
Region
5'UTR
3'UTR
CDS
Intron
5'end
3'end
Zebrafish : Normalised Within-region Signal
GC content
Position w.r.t. TSS (kb)

Plants

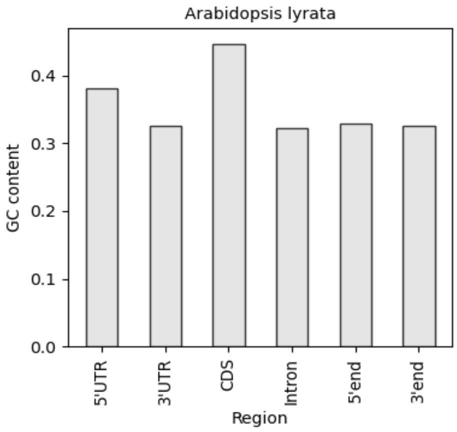
Arabidopsis lyrata
GC content
0.0
0.1
0.2
0.3
0.4
5'UTR
3'UTR
CDS
Intron
5'end
3'end
Region

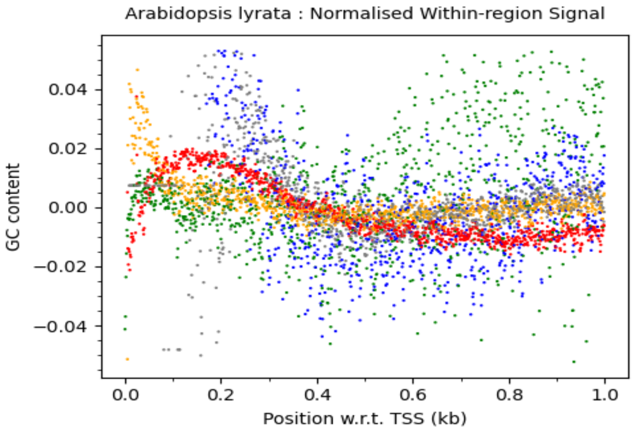
Arabidopsis lyrata : Normalised Within-region Signal
GC content
−0.04
−0.02
0.00
0.02
0.04
0.0
0.2
0.4
0.6
0.8
1.0
Position w.r.t. TSS (kb)

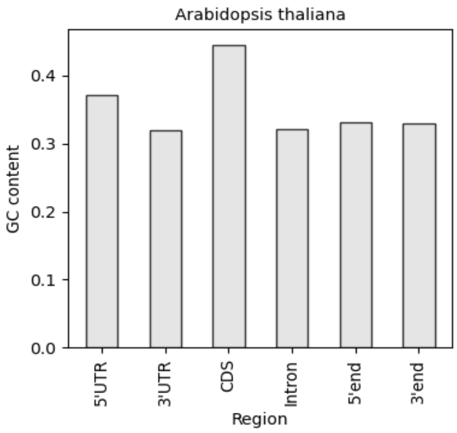
Arabidopsis thaliana
GC content
0.0
0.1
0.2
0.3
0.4
5'UTR
3'UTR
CDS
Intron
5'end
3'end
Region

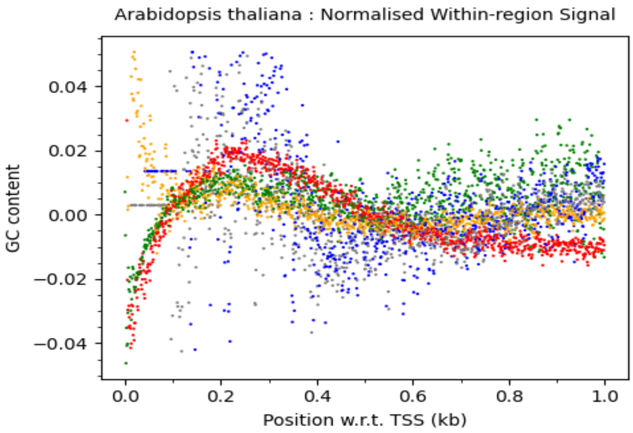
Arabidopsis thaliana : Normalised Within-region Signal
GC content
−0.04
−0.02
0.00
0.02
0.04
0.0
0.2
0.4
0.6
0.8
1.0
Position w.r.t. TSS (kb)

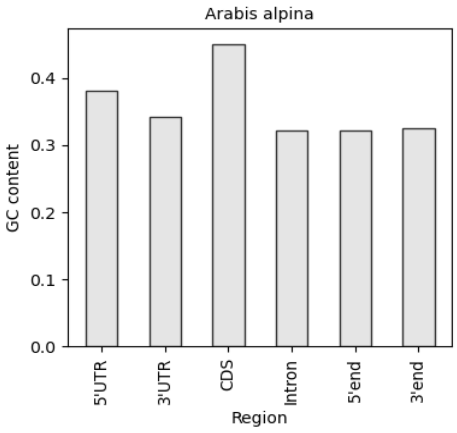
Arabis alpina
GC content
0.0
0.1
0.2
0.3
0.4
5'UTR
3'UTR
CDS
Intron
5'end
3'end
Region

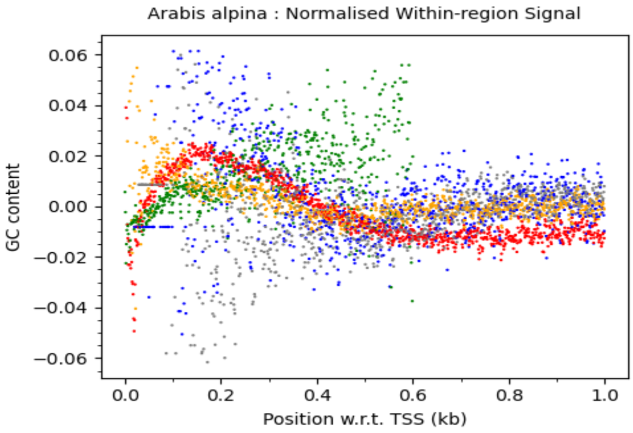
Arabis alpina : Normalised Within-region Signal
GC content
−0.06
−0.04
−0.02
0.00
0.02
0.04
0.06
0.0
0.2
0.4
0.6
0.8
1.0
Position w.r.t. TSS (kb)

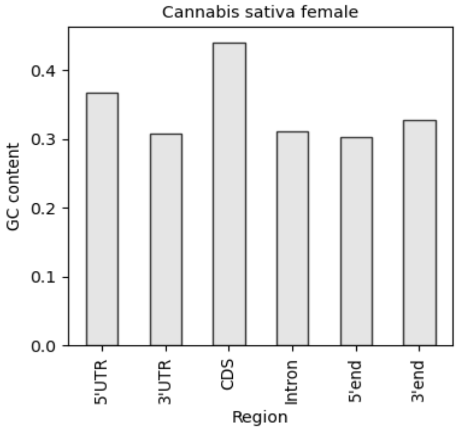
Cannabis sativa female
GC content
0.0
0.1
0.2
0.3
0.4
5'UTR
3'UTR
CDS
Intron
5'end
3'end
Region

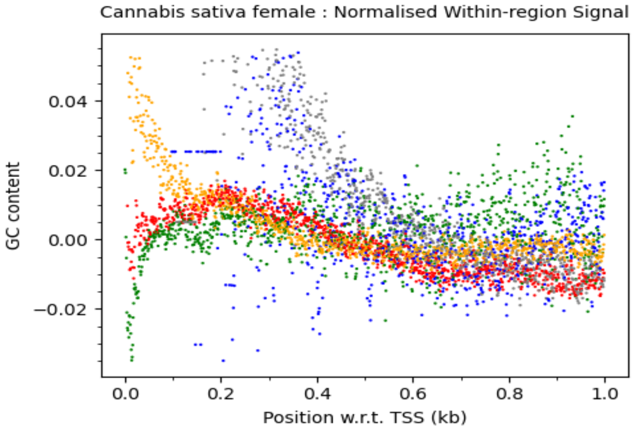
Cannabis sativa female : Normalised Within-region Signal
GC content
−0.02
0.00
0.02
0.04
0.0
0.2
0.4
0.6
0.8
1.0
Position w.r.t. TSS (kb)

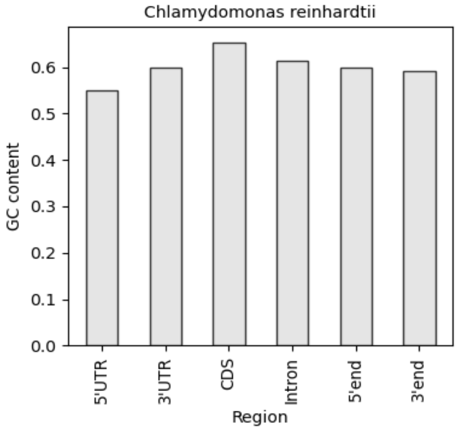
Chlamydomonas reinhardtii
GC content
0.0
0.1
0.2
0.3
0.4
0.5
0.6
5'UTR
3'UTR
CDS
Intron
5'end
3'end
Region

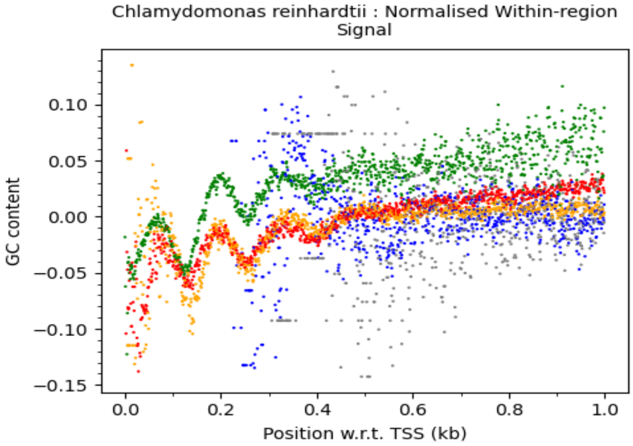
Chlamydomonas reinhardtii : Normalised Within-region Signal
GC content
−0.15
−0.10
−0.05
0.00
0.05
0.10
0.0
0.2
0.4
0.6
0.8
1.0
Position w.r.t. TSS (kb)

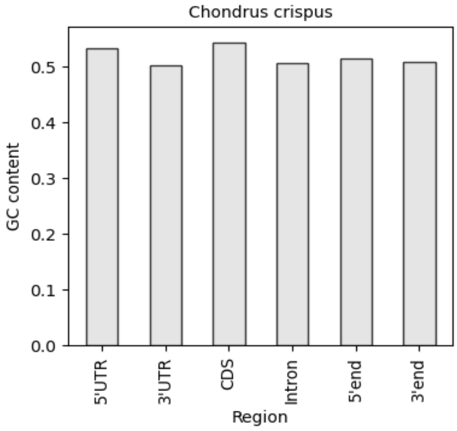
Chondrus crispus
GC content
0.0
0.1
0.2
0.3
0.4
0.5
5'UTR
3'UTR
CDS
Intron
5'end
3'end
Region

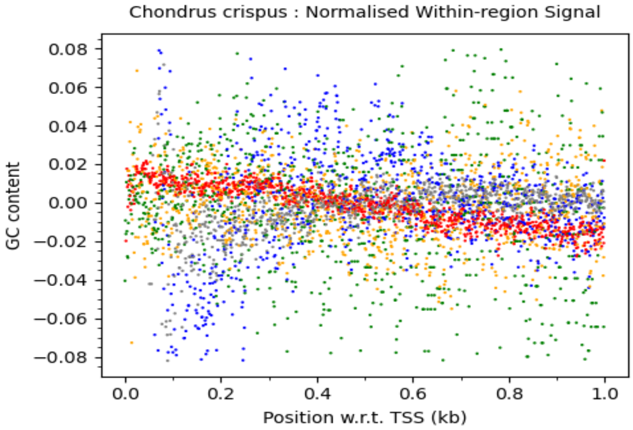
Chondrus crispus : Normalised Within-region Signal
GC content
−0.08
−0.06
−0.04
−0.02
0.00
0.02
0.04
0.06
0.08
0.0
0.2
0.4
0.6
0.8
1.0
Position w.r.t. TSS (kb)

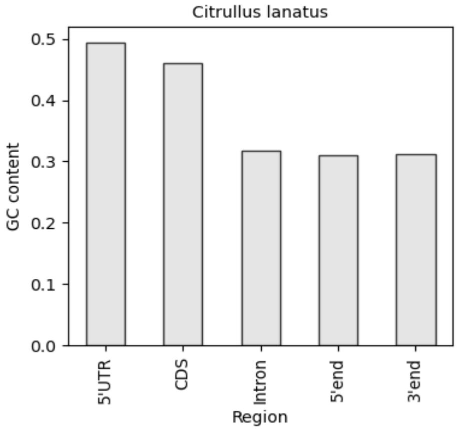
Citrullus lanatus
GC content
0.0
0.1
0.2
0.3
0.4
0.5
5'UTR
CDS
Intron
5'end
3'end
Region

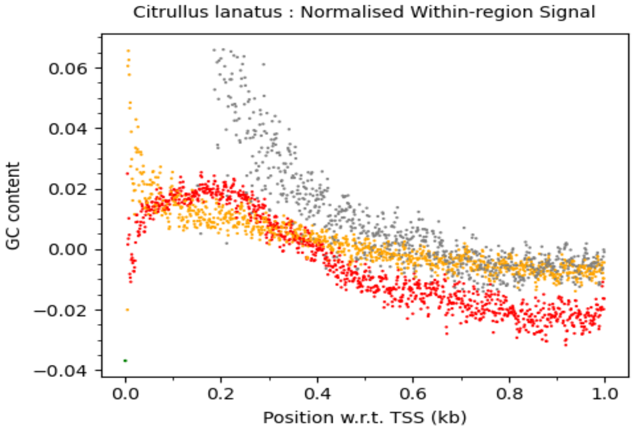
Citrullus lanatus : Normalised Within-region Signal
GC content
−0.04
−0.02
0.00
0.02
0.04
0.06
0.0
0.2
0.4
0.6
0.8
1.0
Position w.r.t. TSS (kb)

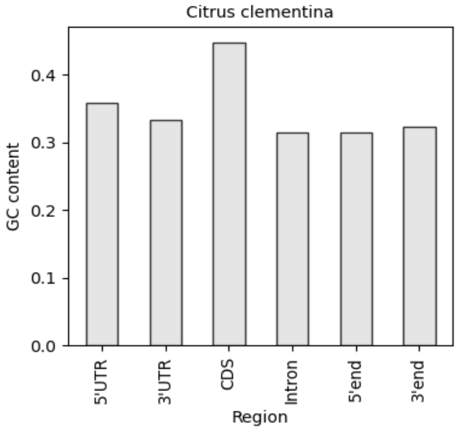
Citrus clementina
GC content
0.0
0.1
0.2
0.3
0.4
5'UTR
3'UTR
CDS
Intron
5'end
3'end
Region

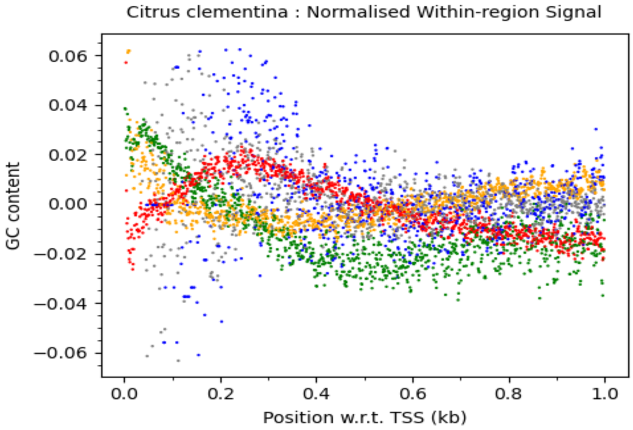
Citrus clementina : Normalised Within-region Signal
GC content
−0.06
−0.04
−0.02
0.00
0.02
0.04
0.06
0.0
0.2
0.4
0.6
0.8
1.0
Position w.r.t. TSS (kb)

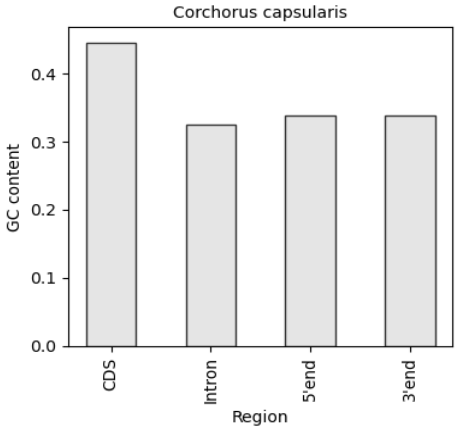
Corchorus capsularis
GC content
0.0
0.1
0.2
0.3
0.4
CDS
Intron
5'end
3'end
Region

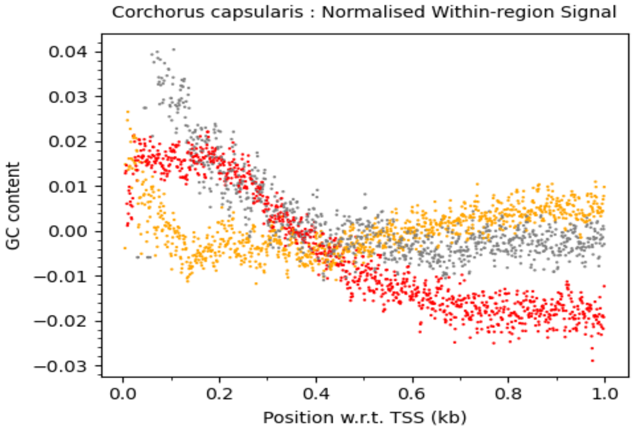
Corchorus capsularis : Normalised Within-region Signal
GC content
−0.03
−0.02
−0.01
0.00
0.01
0.02
0.03
0.04
0.0
0.2
0.4
0.6
0.8
1.0
Position w.r.t. TSS (kb)

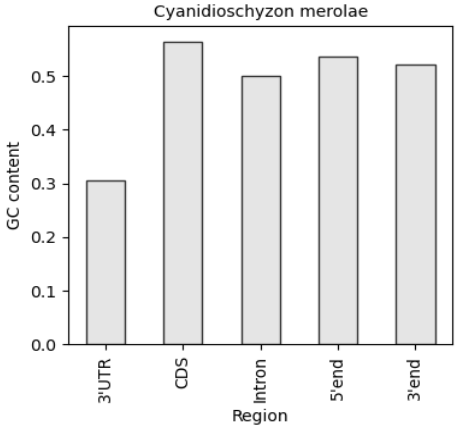
Cyanidioschyzon merolae
GC content
0.0
0.1
0.2
0.3
0.4
0.5
3'UTR
CDS
Intron
5'end
3'end
Region

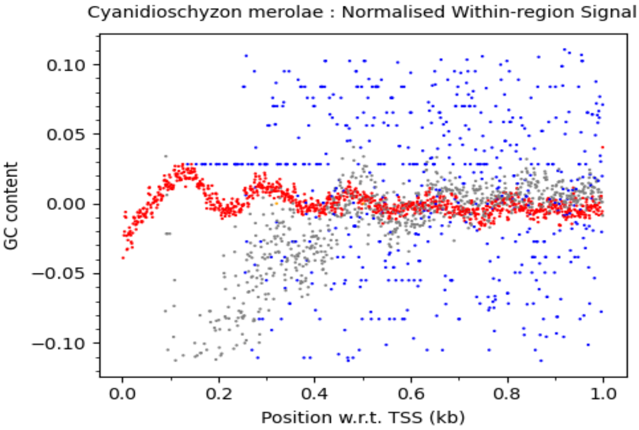
Cyanidioschyzon merolae : Normalised Within-region Signal
GC content
0.10
0.05
0.00
−0.05
−0.10
0.0
0.2
0.4
0.6
0.8
1.0
Position w.r.t. TSS (kb)

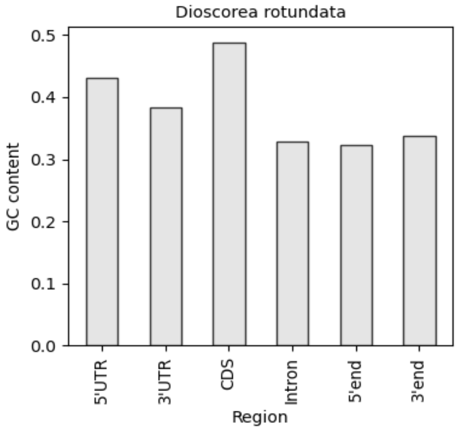
Dioscorea rotundata
GC content
0.0
0.1
0.2
0.3
0.4
0.5
5'UTR
3'UTR
CDS
Intron
5'end
3'end
Region

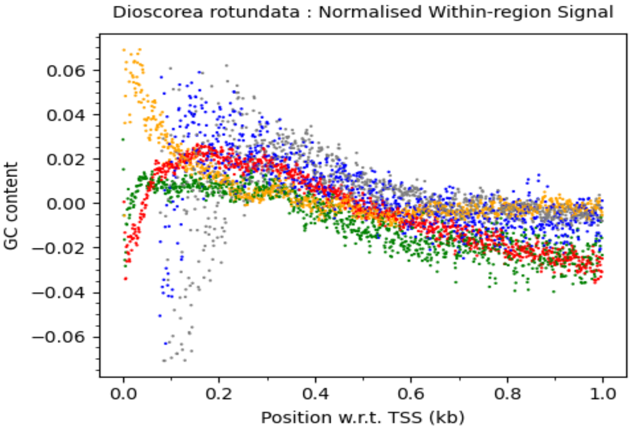
Dioscorea rotundata : Normalised Within-region Signal
GC content
0.06
0.04
0.02
0.00
−0.02
−0.04
−0.06
0.0
0.2
0.4
0.6
0.8
1.0
Position w.r.t. TSS (kb)

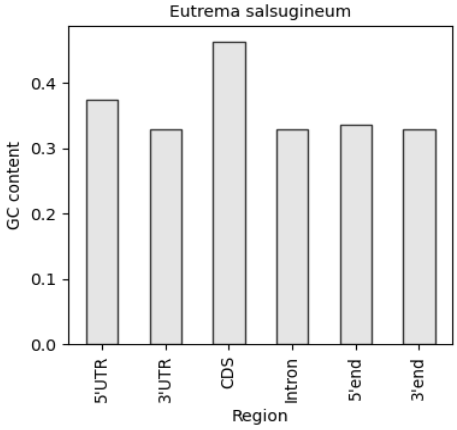
Eutrema salsugineum
GC content
0.0
0.1
0.2
0.3
0.4
5'UTR
3'UTR
CDS
Intron
5'end
3'end
Region

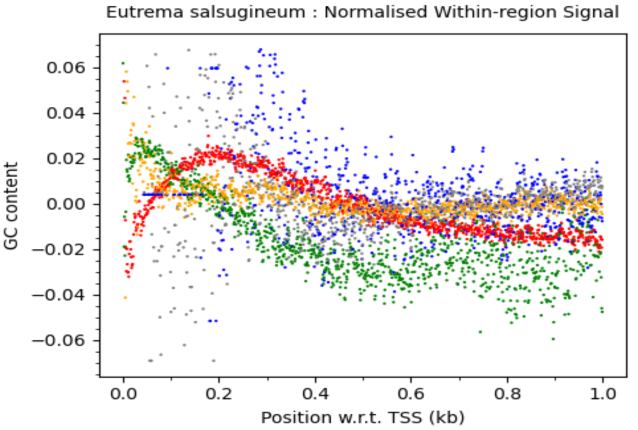
Eutrema salsugineum : Normalised Within-region Signal
GC content
0.06
0.04
0.02
0.00
−0.02
−0.04
−0.06
0.0
0.2
0.4
0.6
0.8
1.0
Position w.r.t. TSS (kb)

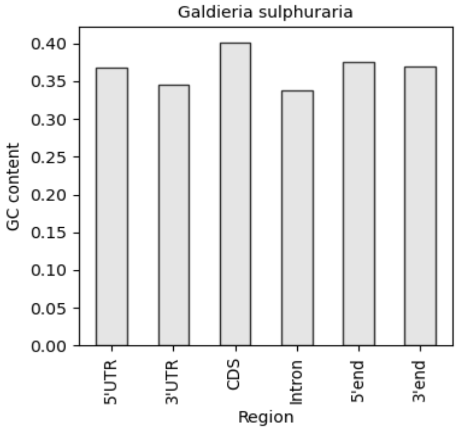
Galdieria sulphuraria
GC content
Region
5'UTR
3'UTR
CDS
Intron
5'end
3'end

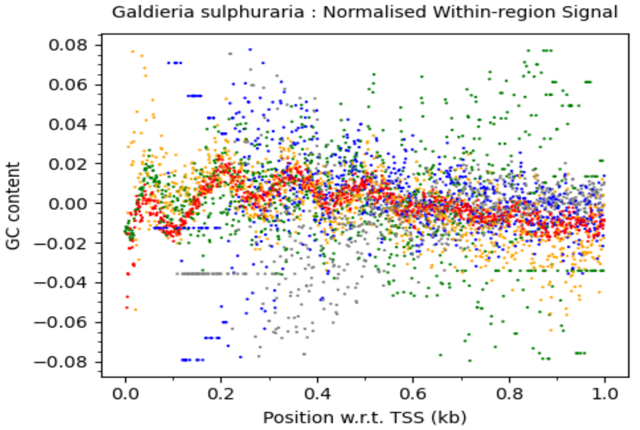
Galdieria sulphuraria : Normalised Within-region Signal
GC content
Position w.r.t. TSS (kb)

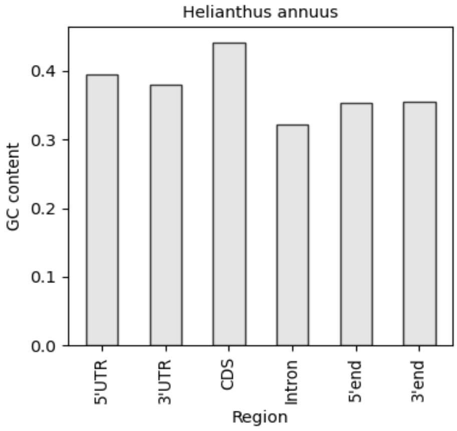
Helianthus annuus
GC content
Region
5'UTR
3'UTR
CDS
Intron
5'end
3'end

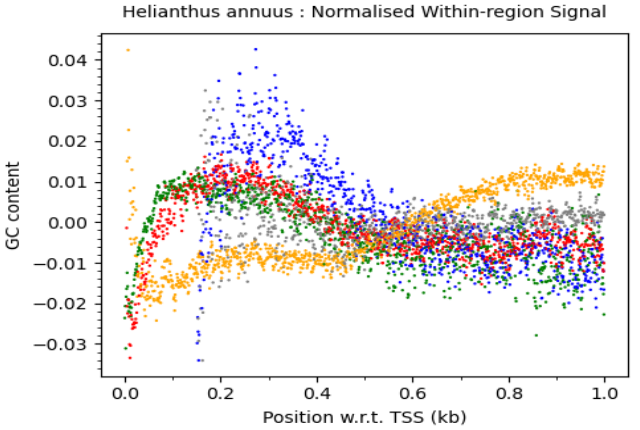
Helianthus annuus : Normalised Within-region Signal
GC content
Position w.r.t. TSS (kb)

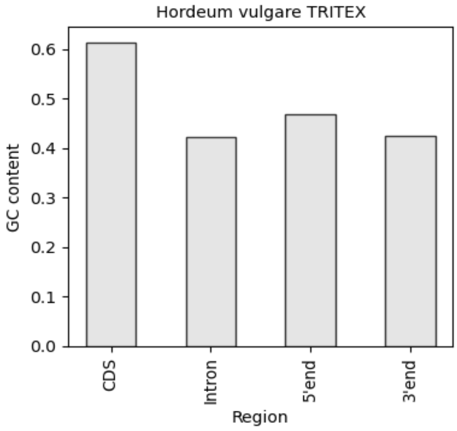
Hordeum vulgare TRITEX
GC content
Region
CDS
Intron
5'end
3'end

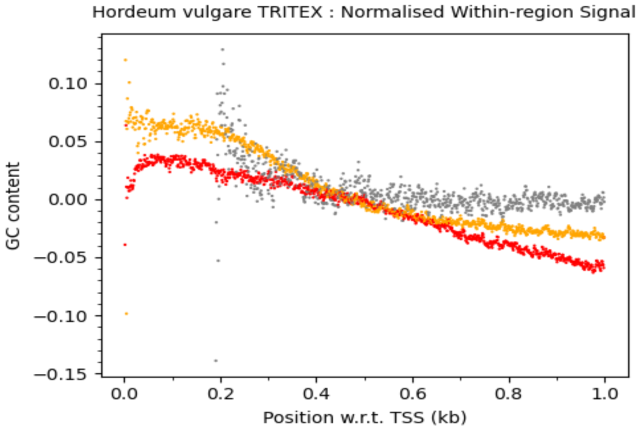
Hordeum vulgare TRITEX : Normalised Within-region Signal
GC content
Position w.r.t. TSS (kb)

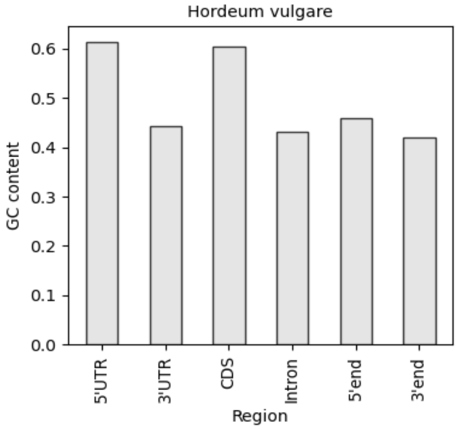
Hordeum vulgare
GC content
Region
5'UTR
3'UTR
CDS
Intron
5'end
3'end

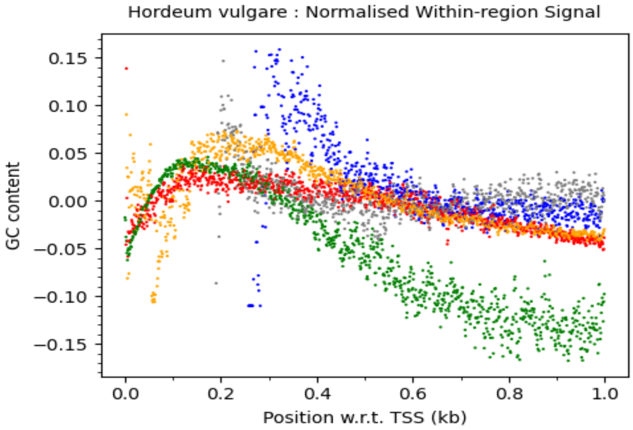
Hordeum vulgare : Normalised Within-region Signal
GC content
Position w.r.t. TSS (kb)

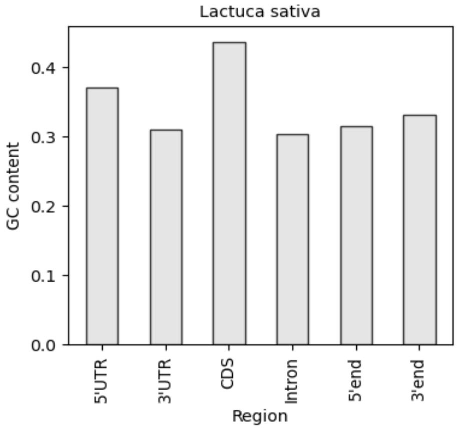
Lactuca sativa
GC content
Region
5'UTR
3'UTR
CDS
Intron
5'end
3'end

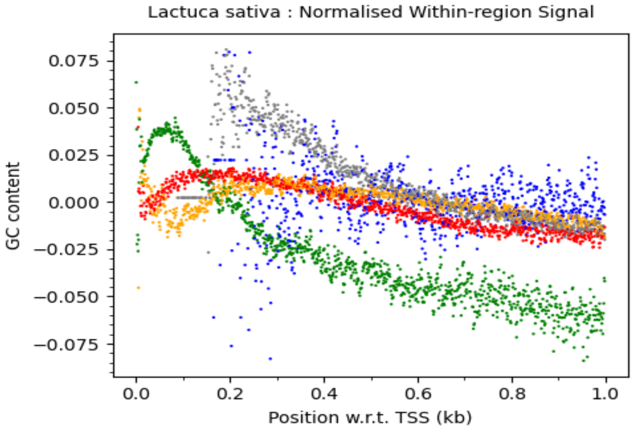
Lactuca sativa : Normalised Within-region Signal
GC content
Position w.r.t. TSS (kb)

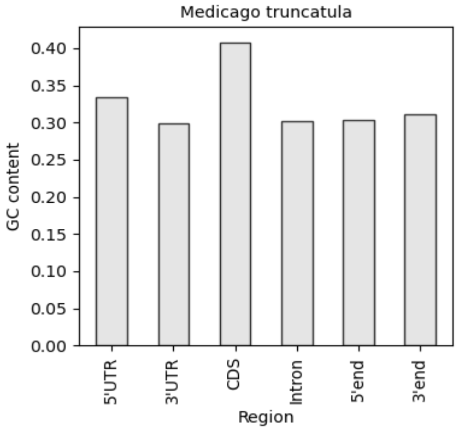
Medicago truncatula
GC content
Region
5'UTR
3'UTR
CDS
Intron
5'end
3'end

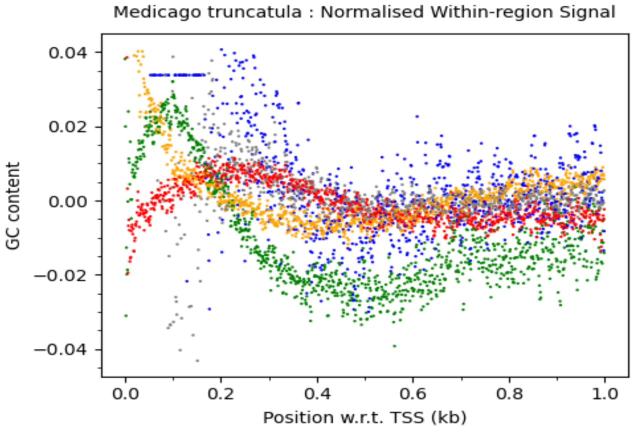
Medicago truncatula : Normalised Within-region Signal
GC content
Position w.r.t. TSS (kb)

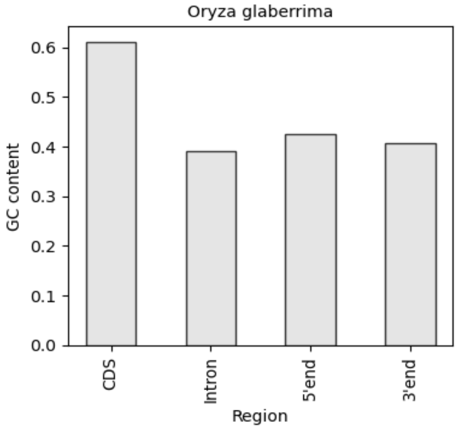
Oryza glaberrima
GC content
Region
CDS
Intron
5'end
3'end
0.0
0.1
0.2
0.3
0.4
0.5
0.6

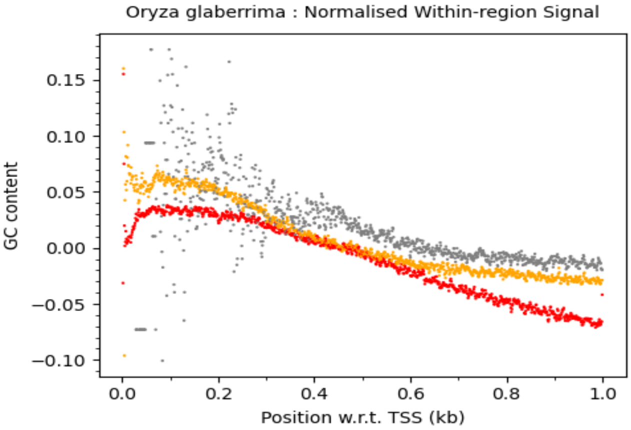
Oryza glaberrima : Normalised Within-region Signal
GC content
Position w.r.t. TSS (kb)
0.15
0.10
0.05
0.00
−0.05
−0.10
0.0
0.2
0.4
0.6
0.8
1.0

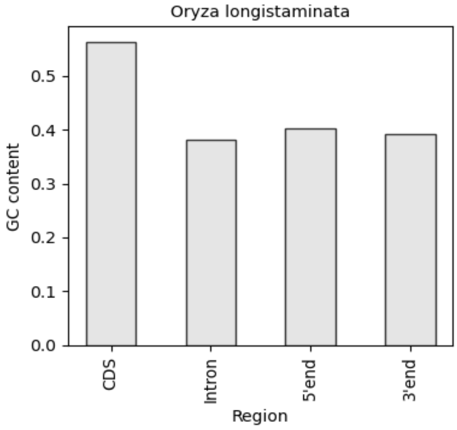
Oryza longistaminata
GC content
Region
CDS
Intron
5'end
3'end
0.0
0.1
0.2
0.3
0.4
0.5

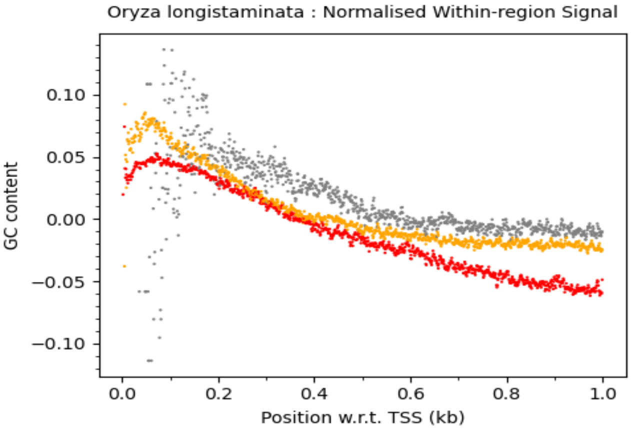
Oryza longistaminata : Normalised Within-region Signal
GC content
Position w.r.t. TSS (kb)
0.10
0.05
0.00
−0.05
−0.10
0.0
0.2
0.4
0.6
0.8
1.0

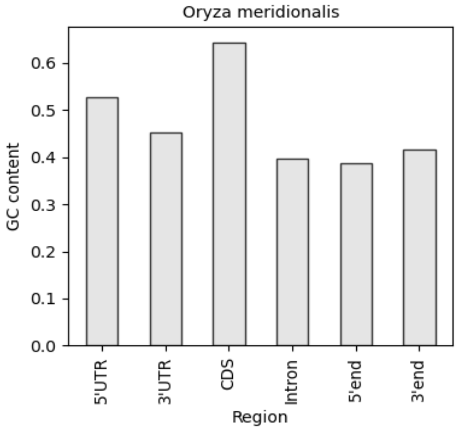
Oryza meridionalis
GC content
Region
5'UTR
3'UTR
CDS
Intron
5'end
3'end
0.0
0.1
0.2
0.3
0.4
0.5
0.6

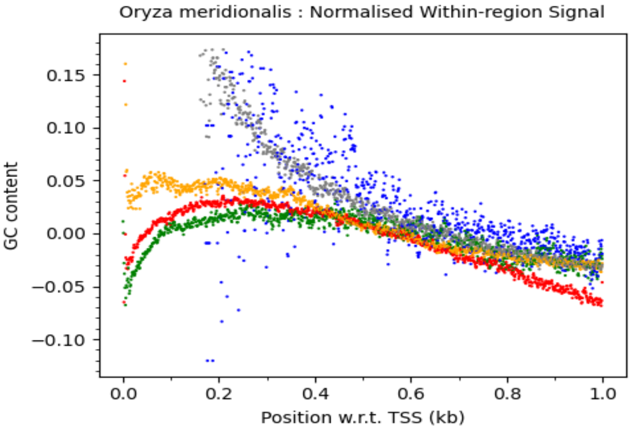
Oryza meridionalis : Normalised Within-region Signal
GC content
Position w.r.t. TSS (kb)
0.15
0.10
0.05
0.00
−0.05
−0.10
0.0
0.2
0.4
0.6
0.8
1.0

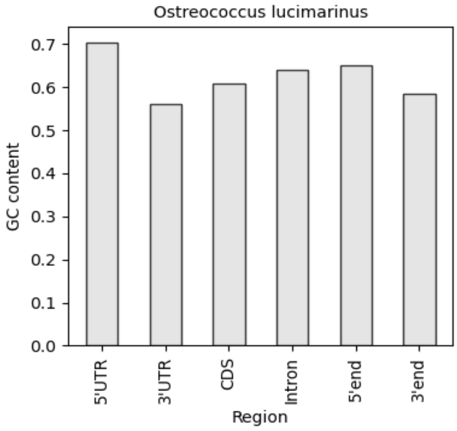
Ostreococcus lucimarinus
GC content
Region
5'UTR
3'UTR
CDS
Intron
5'end
3'end

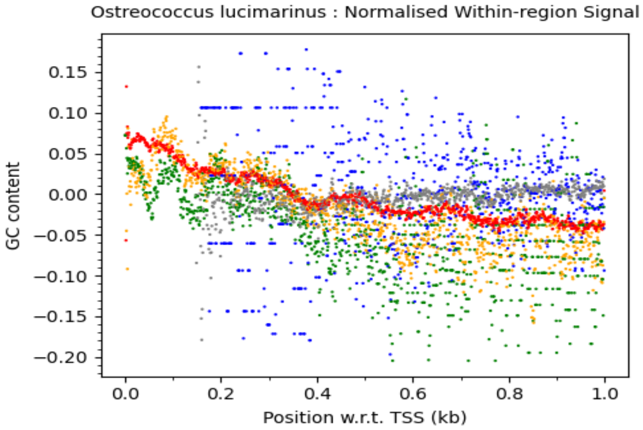
Ostreococcus lucimarinus : Normalised Within-region Signal
GC content
Position w.r.t. TSS (kb)

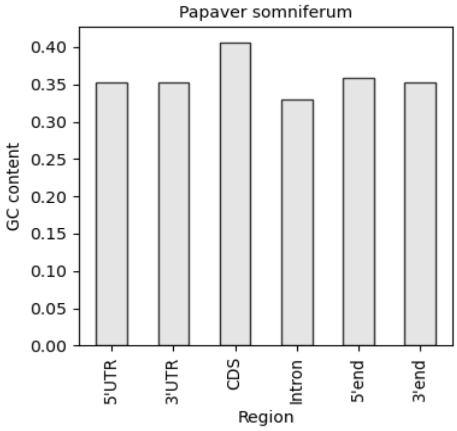
Papaver somniferum
GC content
Region
5'UTR
3'UTR
CDS
Intron
5'end
3'end

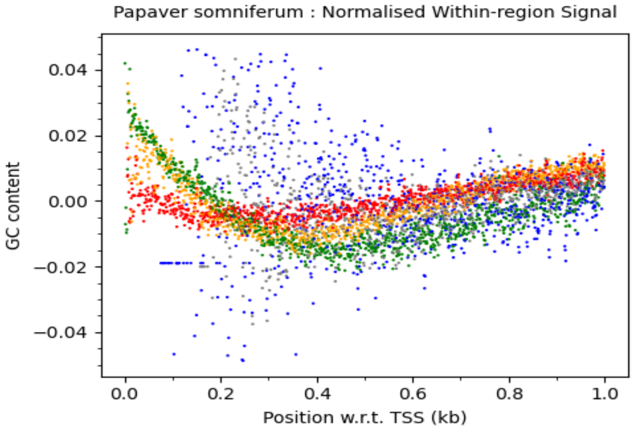
Papaver somniferum : Normalised Within-region Signal
GC content
Position w.r.t. TSS (kb)

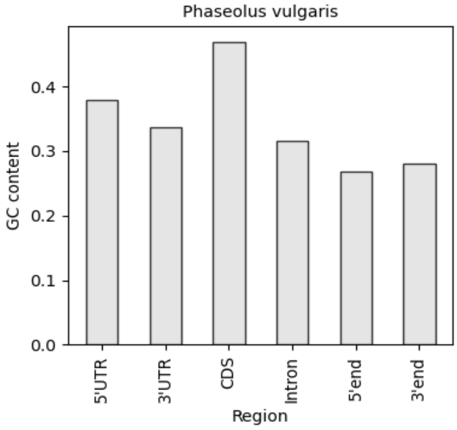
Phaseolus vulgaris
GC content
Region
5'UTR
3'UTR
CDS
Intron
5'end
3'end

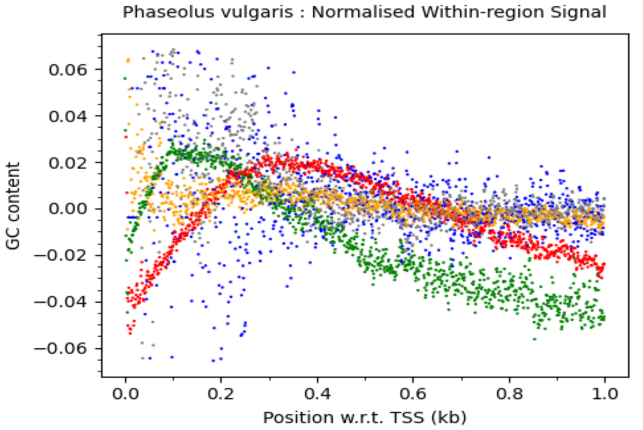
Phaseolus vulgaris : Normalised Within-region Signal
GC content
Position w.r.t. TSS (kb)

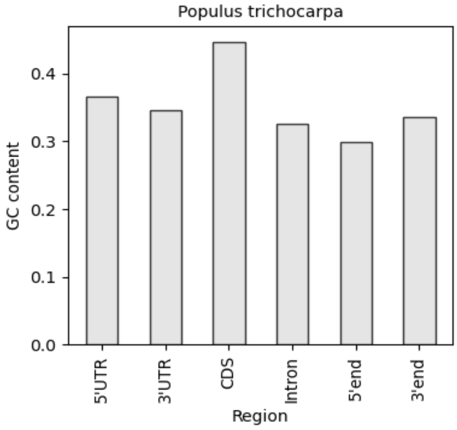
Populus trichocarpa
GC content
0.0
0.1
0.2
0.3
0.4
5'UTR
3'UTR
CDS
Intron
5'end
3'end
Region

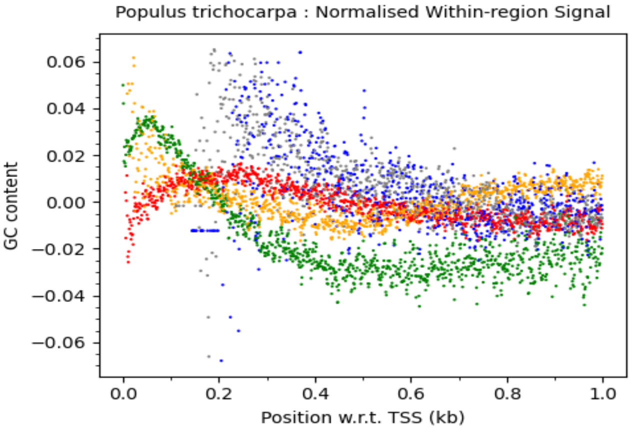
Populus trichocarpa : Normalised Within-region Signal
GC content
−0.06
−0.04
−0.02
0.00
0.02
0.04
0.06
0.0
0.2
0.4
0.6
0.8
1.0
Position w.r.t. TSS (kb)

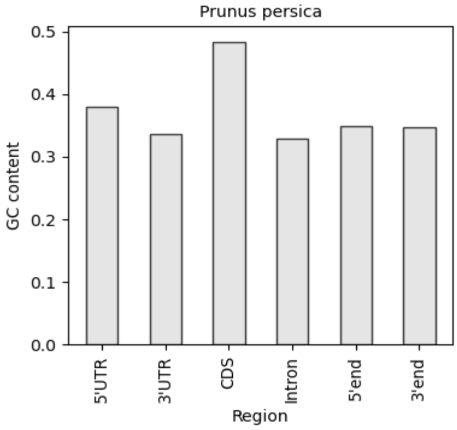
Prunus persica
GC content
0.0
0.1
0.2
0.3
0.4
0.5
5'UTR
3'UTR
CDS
Intron
5'end
3'end
Region

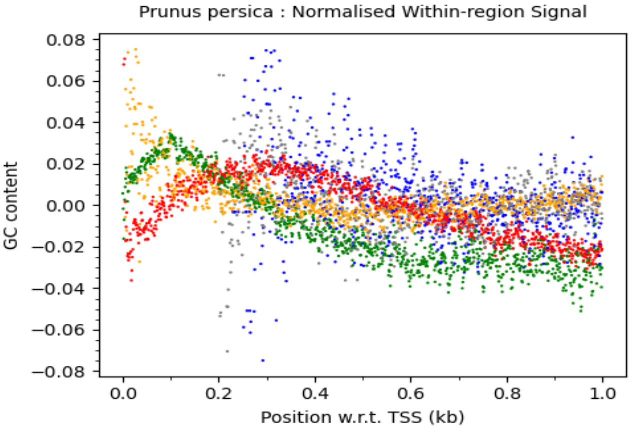
Prunus persica : Normalised Within-region Signal
GC content
−0.08
−0.06
−0.04
−0.02
0.00
0.02
0.04
0.06
0.08
0.0
0.2
0.4
0.6
0.8
1.0
Position w.r.t. TSS (kb)

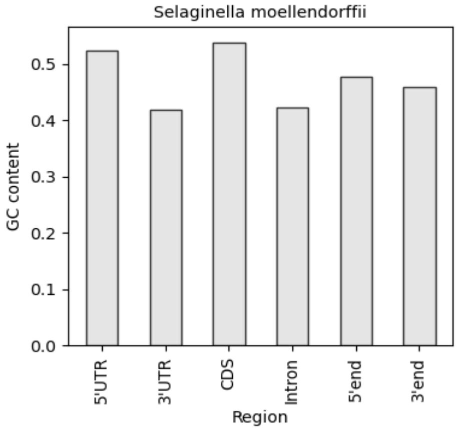
Selaginella moellendorffii
GC content
0.0
0.1
0.2
0.3
0.4
0.5
5'UTR
3'UTR
CDS
Intron
5'end
3'end
Region

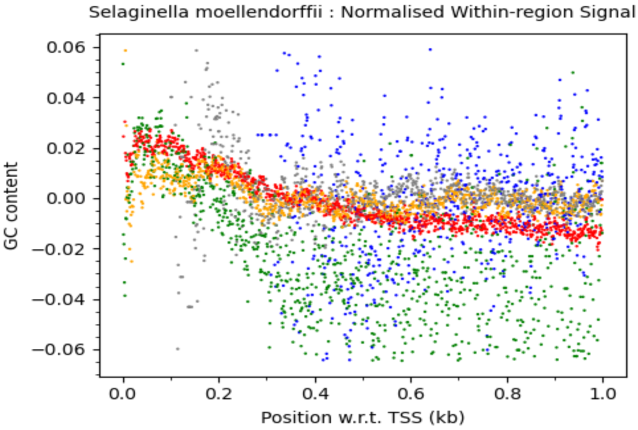
Selaginella moellendorffii : Normalised Within-region Signal
GC content
−0.06
−0.04
−0.02
0.00
0.02
0.04
0.06
0.0
0.2
0.4
0.6
0.8
1.0
Position w.r.t. TSS (kb)

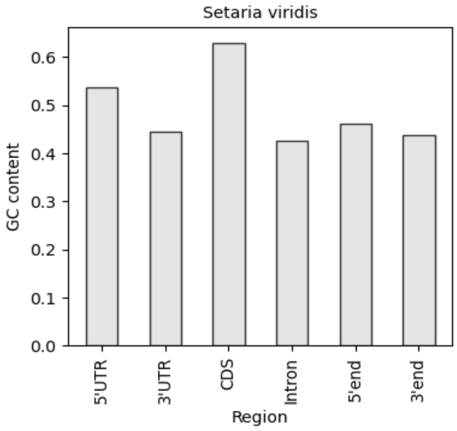
Setaria viridis
GC content
0.0
0.1
0.2
0.3
0.4
0.5
0.6
5'UTR
3'UTR
CDS
Intron
5'end
3'end
Region

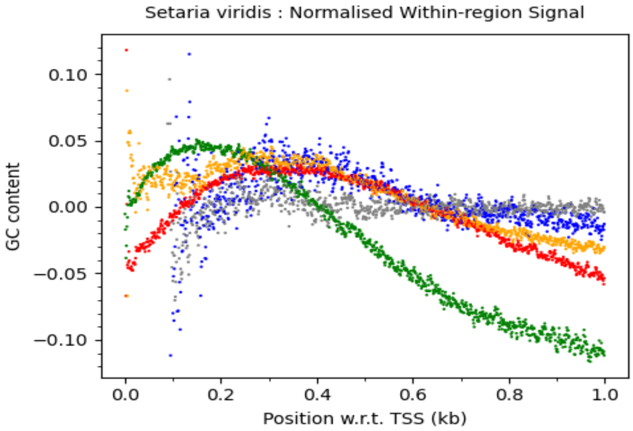
Setaria viridis : Normalised Within-region Signal
GC content
0.10
0.05
0.00
−0.05
−0.10
0.0
0.2
0.4
0.6
0.8
1.0
Position w.r.t. TSS (kb)

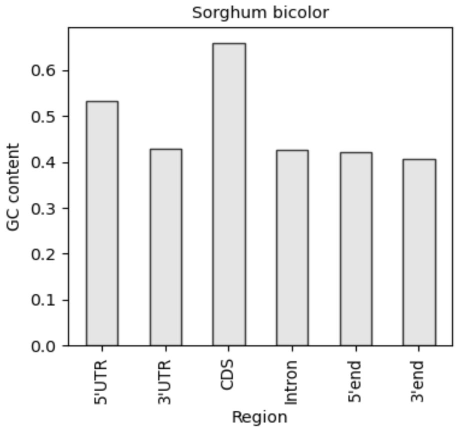
Sorghum bicolor
GC content
0.0
0.1
0.2
0.3
0.4
0.5
0.6
5'UTR
3'UTR
CDS
Intron
5'end
3'end
Region

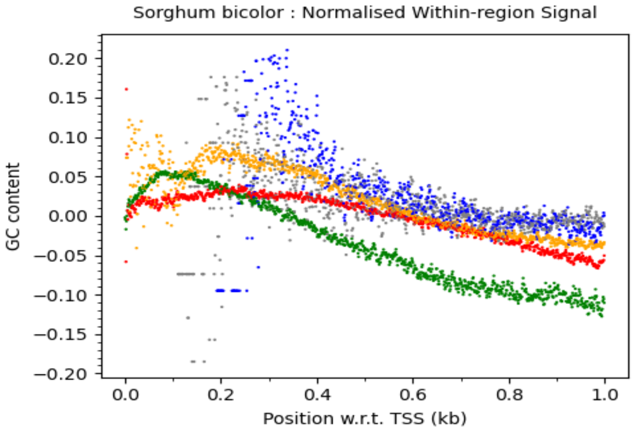
Sorghum bicolor : Normalised Within-region Signal
GC content
0.20
0.15
0.10
0.05
0.00
−0.05
−0.10
−0.15
−0.20
0.0
0.2
0.4
0.6
0.8
1.0
Position w.r.t. TSS (kb)

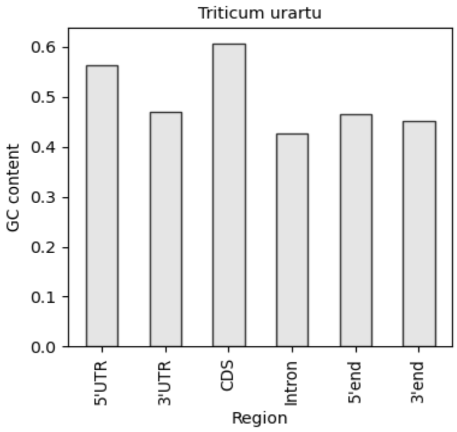
Triticum urartu
GC content
0.0
0.1
0.2
0.3
0.4
0.5
0.6
5'UTR
3'UTR
CDS
Intron
5'end
3'end
Region

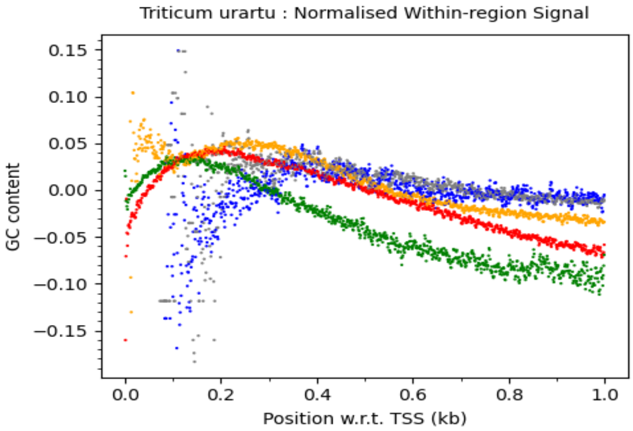
Triticum urartu : Normalised Within-region Signal
GC content
0.15
0.10
0.05
0.00
−0.05
−0.10
−0.15
0.0
0.2
0.4
0.6
0.8
1.0
Position w.r.t. TSS (kb)

Figure S4

Estimation of the optimal number of clusters for vertebrates and plants using the Calinski-Harabasz (CH) Index and the silhouette score. The CH Index is based on the ratio of between-cluster variance to within-cluster variance. It evaluates the degree of separation between clusters and the degree of similarity between objects within the same cluster. The CH Index takes the form of $(a * between - cluster\ variance)/(b * within - cluster\ variance)$, where a and b are weighting factors. Similarly, the silhouette score for a datapoint is a measure of how similar a datapoint is to its own cluster compared to other clusters. The score ranges from -1 to $+1$, where $+1$ indicates that the point is similar to its own cluster and far from other clusters. If most datapoints have a high value, then the clustering configuration is appropriate. The CH Index measures the overall quality of clustering based on the variance in the data, while the silhouette score evaluates the individual quality of each object's assignment to its cluster. In vertebrates and plants, both scores maximize at two clusters when the parameters of the between-region signal and the constituents of the within-region signal are taken into account.

Vertebrates

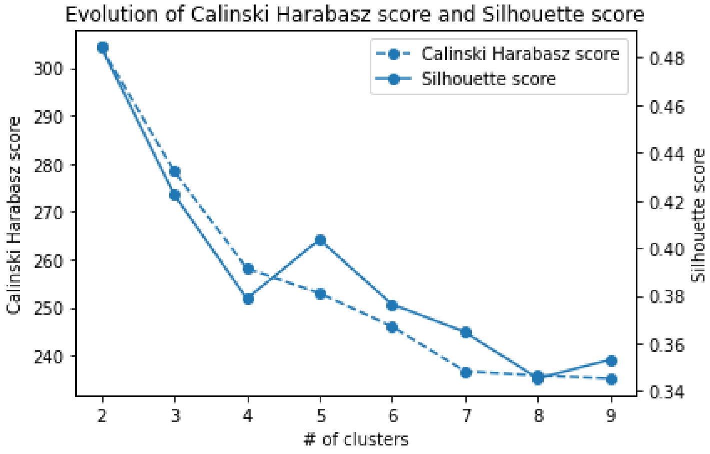

Plants

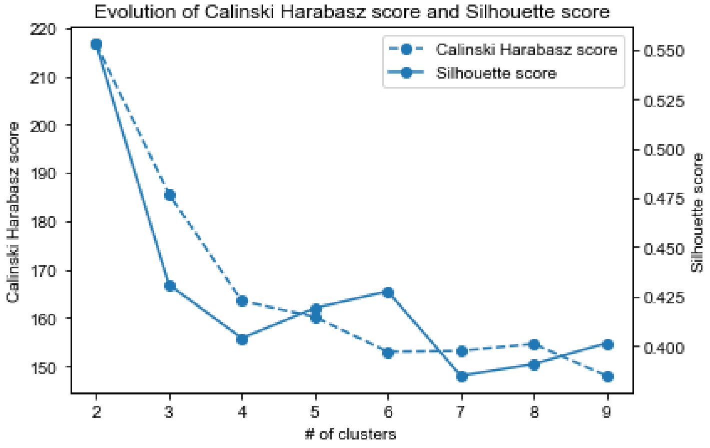

Figure S5

Vertebrates

The diagram shows the clusters formed by the hierarchical clustering algorithm based on Ward's criterion and using the signal size of the between-region signal and of the within-region signal. The values give the level of dissimilarity (distance) between the organisms/clusters. The color of the line indicates the cluster and the color

of the box the taxonomic class. Ascidiacea, Amphibia, Chondrichthyes, Actinopteri, Myxini, and Hyperoartia are grouped as 'Others' due to the low number of species in each of these classes. The height of the branches indicates the dissimilarity between clusters. The between-region signal and within-region signal contribute 8% and 92% respectively in the cluster formation.

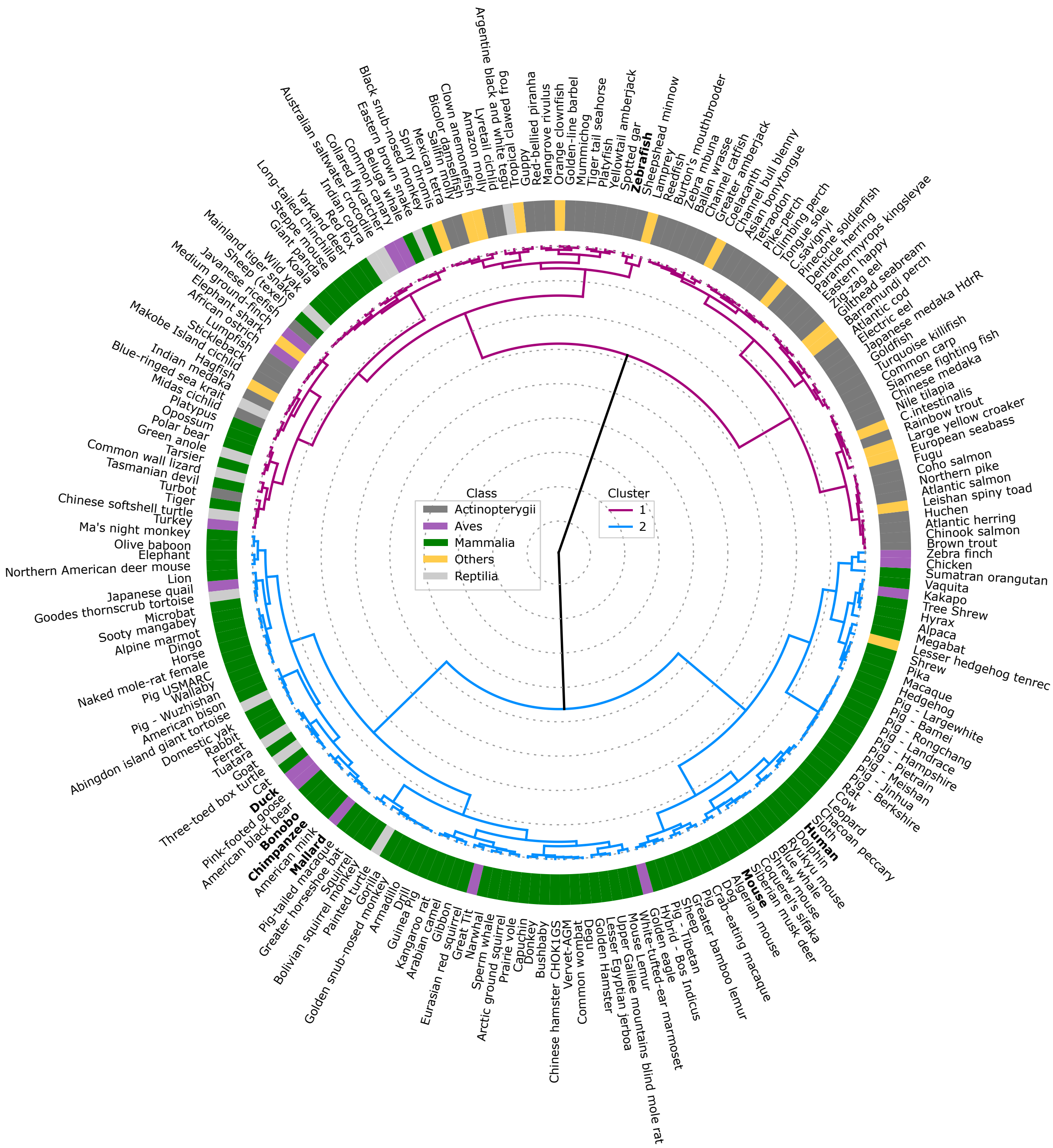

Plants

The figure depicts the clustering based on signal size, with (a) showing clusters formed using between-region signals and components of within-region signals, and (c) presenting the clusters created from between-region and within-region signals. In (b), the relative significance of features contributing to cluster formation in (a) is displayed. For the clusters in (c), the between-region signal (BR) accounts for 45% of the feature importance, while the within-region signal size contributes the remaining 55%.

(a)

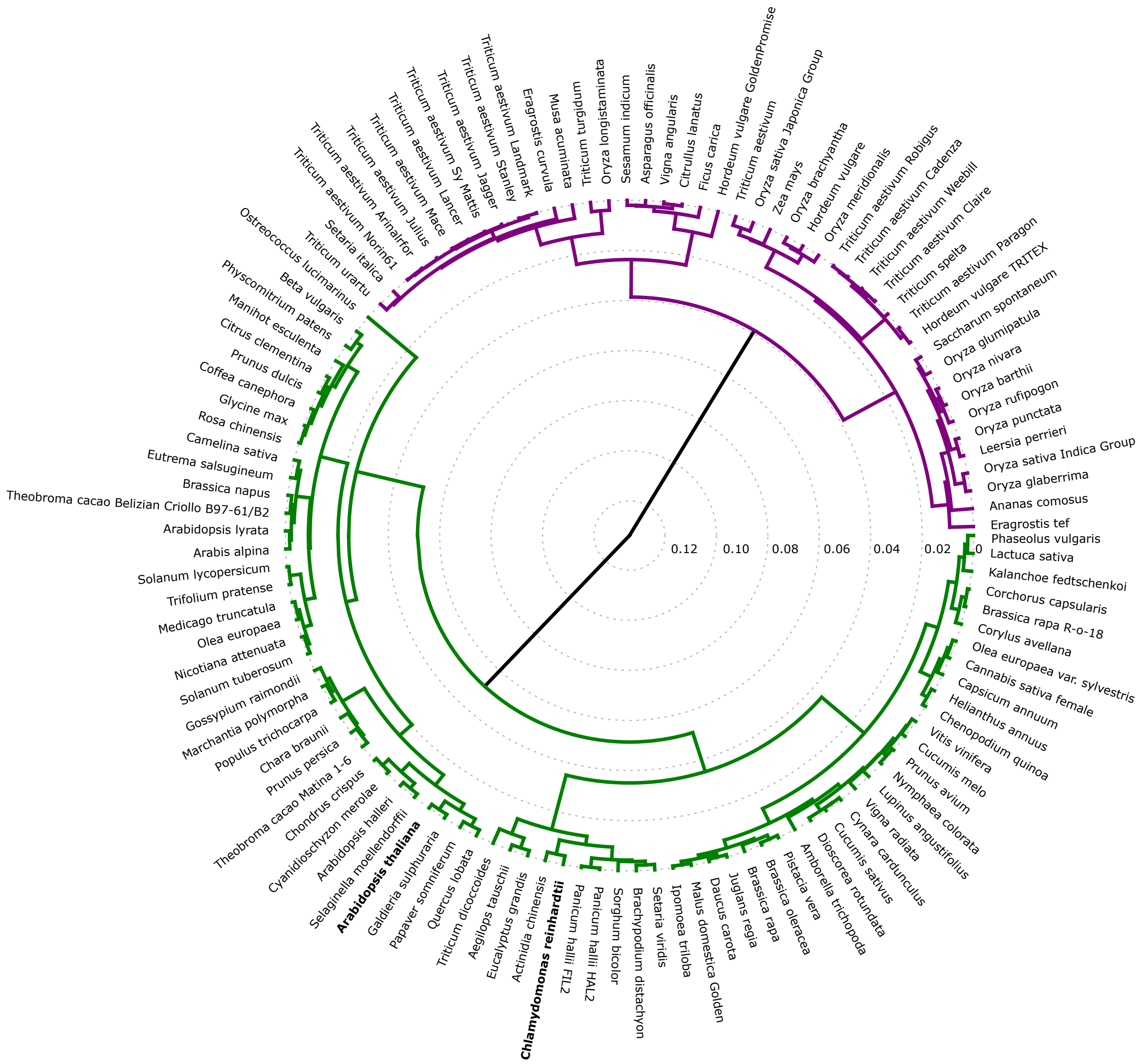
0.12
0.10
0.08
0.06
0.04
0.02
0
Chlamydomonas reinhardtii
Arabidopsis thaliana

(b)

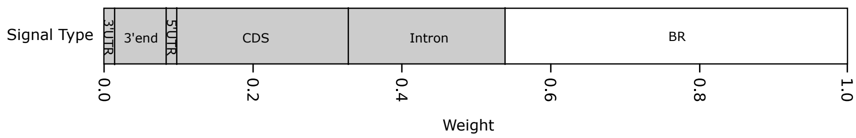
Signal Type
3'UTR
3'end
5'UTR
CDS
Intron
BR
0.0
0.2
0.4
0.6
0.8
1.0
Weight

(c)

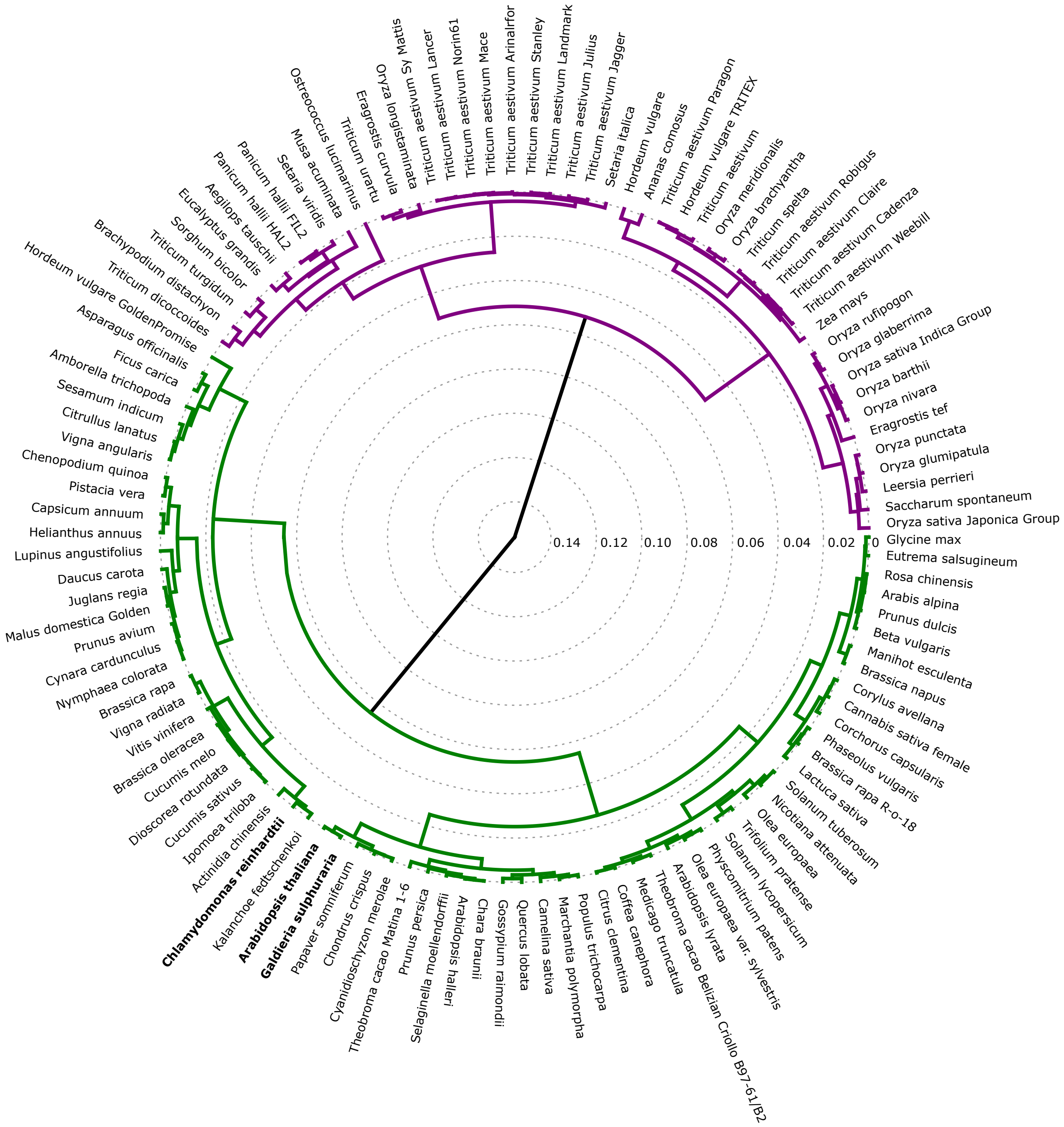
Ostreococcus lucimarinus
Triticum urartu
Eragrostis curvula
Oryza longistaminata
Triticum aestivum SY Mattis
Triticum aestivum Lancer
Triticum aestivum Norin61
Triticum aestivum Mace
Triticum aestivum ArinaLrFor
Triticum aestivum Stanley
Triticum aestivum Landmark
Triticum aestivum Julius
Triticum aestivum Jagger
Setaria italica
Hordeum vulgare
Ananas comosus
Triticum aestivum Paragon
Hordeum vulgare TRITEX
Triticum aestivum
Oryza meridionalis
Oryza brachyantha
Triticum spelta
Triticum aestivum Robigus
Triticum aestivum Claire
Triticum aestivum Cadenza
Triticum aestivum Weebill
Zea mays
Oryza rufipogon
Oryza glaberrima
Oryza sativa Indica Group
Oryza barthii
Oryza nivara
Eragrostis tef
Oryza punctata
Oryza glumipatula
Leersia perrieri
Saccharum spontaneum
Oryza sativa Japonica Group
Glycine max
Eutrema salsugineum
Rosa chinensis
Arabis alpina
Prunus dulcis
Beta vulgaris
Manihot esculenta
Brassica napus
Corylus avellana
Cannabis sativa female
Corchorus capsularis
Phaseolus vulgaris
Brassica rapa R-o-18
Lactuca sativa
Solanum tuberosum
Nicotiana attenuata
Olea europaea
Trifolium pratense
Solanum lycopersicum
Physcomitrium patens
Olea europaea var. sylvestris
Arabidopsis lyrata
Theobroma cacao Belizian Criollo B97-61/B2
Medicago truncatula
Coffea canephora
Citrus clementina
Populus trichocarpa
Marchantia polymorpha
Camelina sativa
Quercus lobata
Gossypium raimondii
Chara braunii
Arabidopsis halleri
Selaginella moellendorffii
Prunus persica
Theobroma cacao Matina 1-6
Cyanidioschyzon merolae
Chondrus crispus
Papaver somniferum
Galdieria sulphuraria
Arabidopsis thaliana
Kalanchoe fedtschenkoi
Chlamydomonas reinhardtii
Actinidia chinensis
Ipomoea triloba
Cucumis sativus
Dioscorea rotundata
Cucumis melo
Brassica oleracea
Vitis vinifera
Vigna radiata
Brassica rapa
Nymphaea colorata
Cynara cardunculus
Prunus avium
Malus domestica Golden
Juglans regia
Daucus carota
Lupinus angustifolius
Helianthus annuus
Capsicum annuum
Pistacia vera
Chenopodium quinoa
Vigna angularis
Citrullus lanatus
Sesamum indicum
Amborella trichopoda
Ficus carica
Asparagus officinalis
Hordeum vulgare GoldenPromise
Triticum dicoccoides
Brachypodium distachyon
Triticum turgidum
Sorghum bicolor
Eucalyptus grandis
Aegilops tauschii
Panicum hallii HAL2
Panicum hallii FIL2
Setaria viridis
Musa acuminata
0.14
0.12
0.10
0.08
0.06
0.04
0.02
0

Figure S6

Comparison of full GC signal, between-region signal, and within-region signal between DNA (black) and the mRNA sequence (red) for selected vertebrates and plants. The introns were removed from the pre-mRNA (DNA) sequence to get the mRNA sequence. The mRNA was centered again at the TSS and the GC signal was calculated 1000 bp downstream of the TSS.

Vertebrates

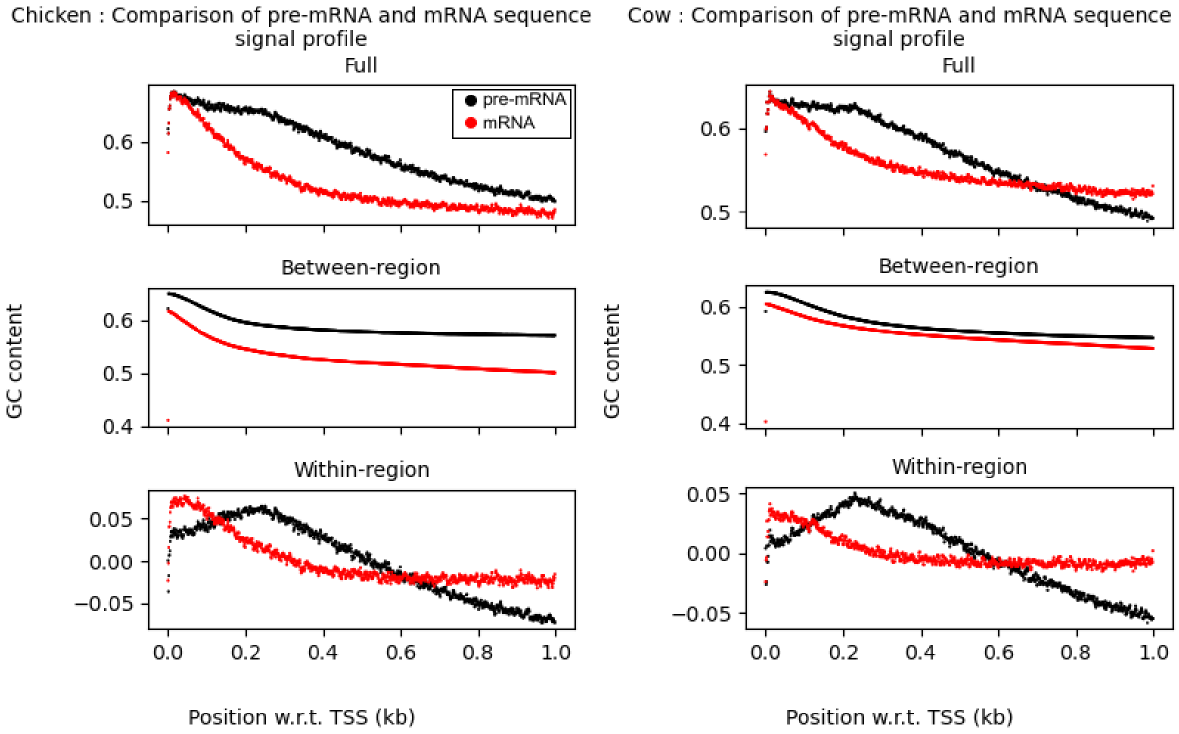

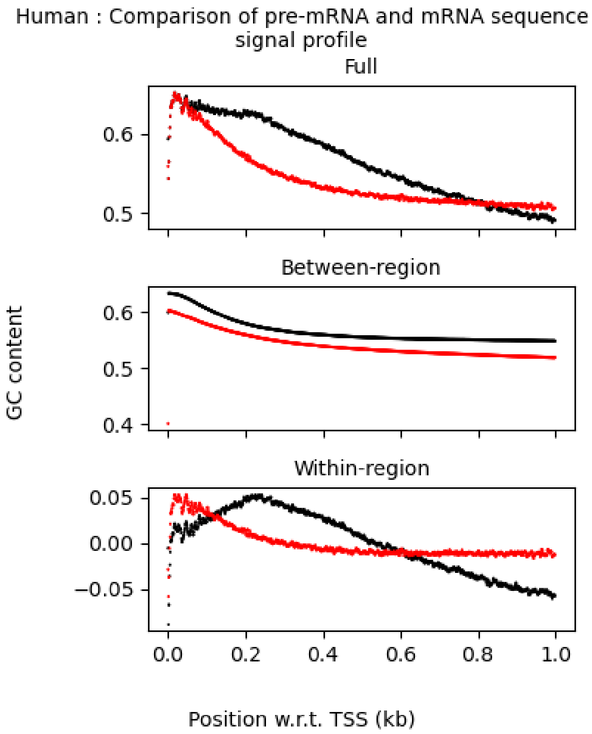
Human : Comparison of pre-mRNA and mRNA sequence signal profile
Full
Between-region
Within-region
GC content
Position w.r.t. TSS (kb)

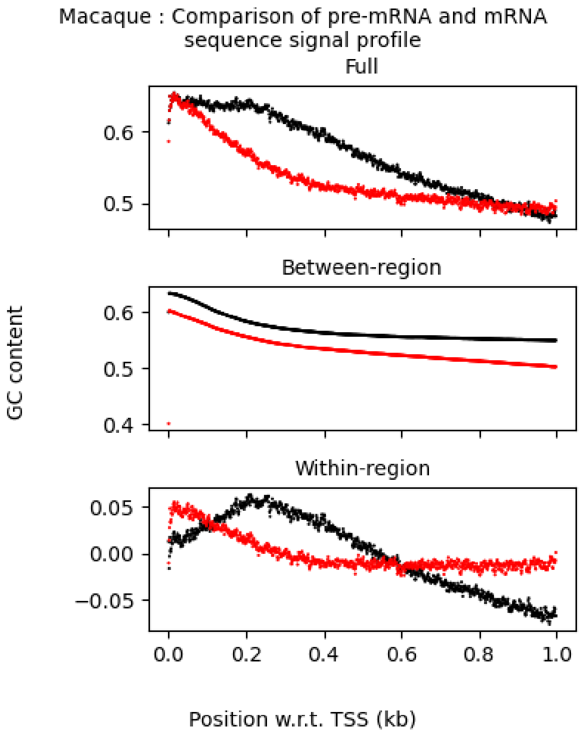
Macaque : Comparison of pre-mRNA and mRNA sequence signal profile
Full
Between-region
Within-region
GC content
Position w.r.t. TSS (kb)

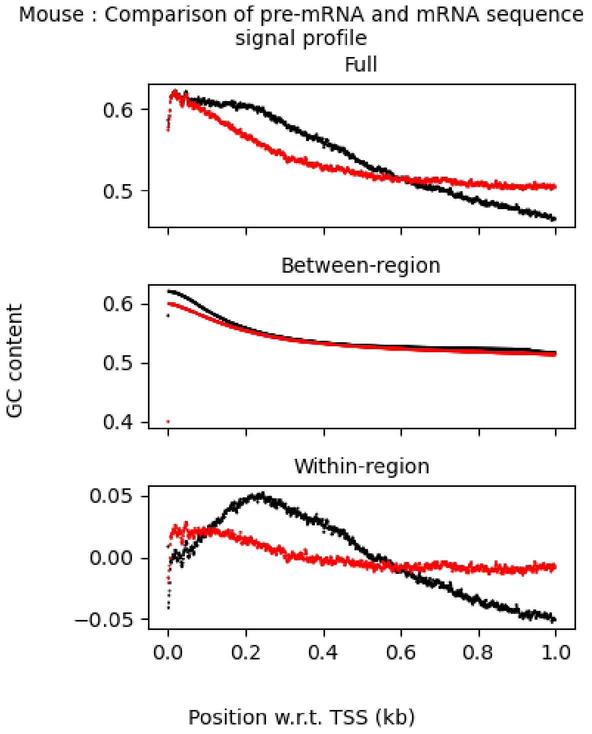
Mouse : Comparison of pre-mRNA and mRNA sequence signal profile
Full
Between-region
Within-region
GC content
Position w.r.t. TSS (kb)

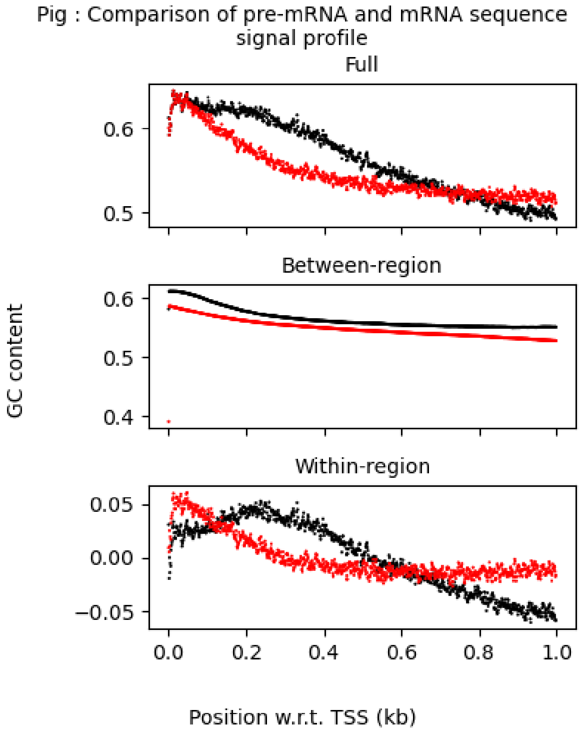
Pig : Comparison of pre-mRNA and mRNA sequence signal profile
Full
Between-region
Within-region
GC content
Position w.r.t. TSS (kb)

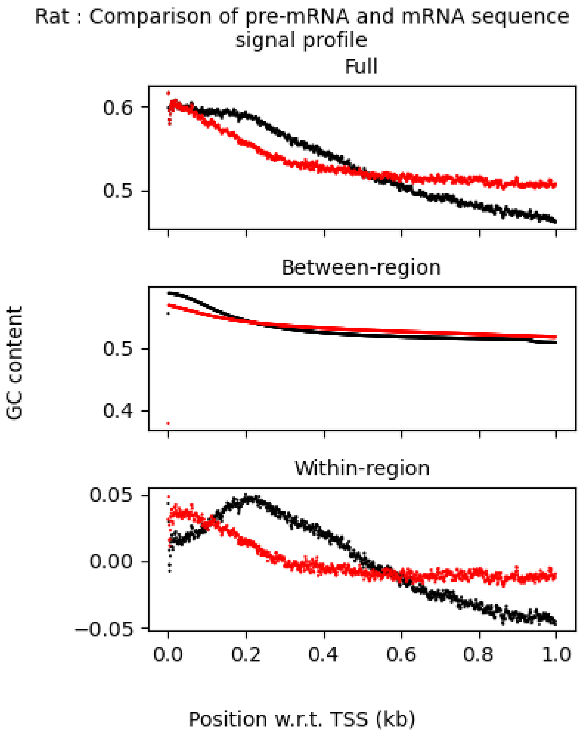
Rat : Comparison of pre-mRNA and mRNA sequence signal profile
Full
Between-region
Within-region
GC content
0.6
0.5
0.4
0.05
0.00
−0.05
0.0 0.2 0.4 0.6 0.8 1.0
Position w.r.t. TSS (kb)

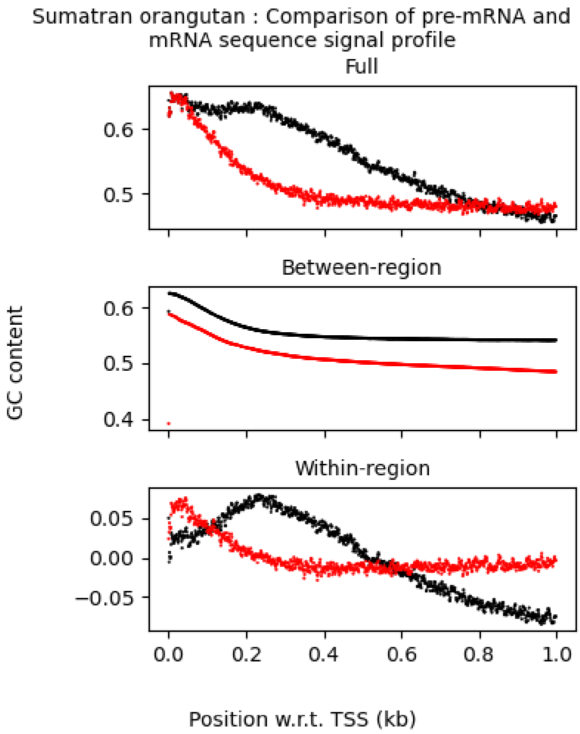
Sumatran orangutan : Comparison of pre-mRNA and mRNA sequence signal profile
Full
Between-region
Within-region
GC content
0.6
0.5
0.4
0.05
0.00
−0.05
0.0 0.2 0.4 0.6 0.8 1.0
Position w.r.t. TSS (kb)

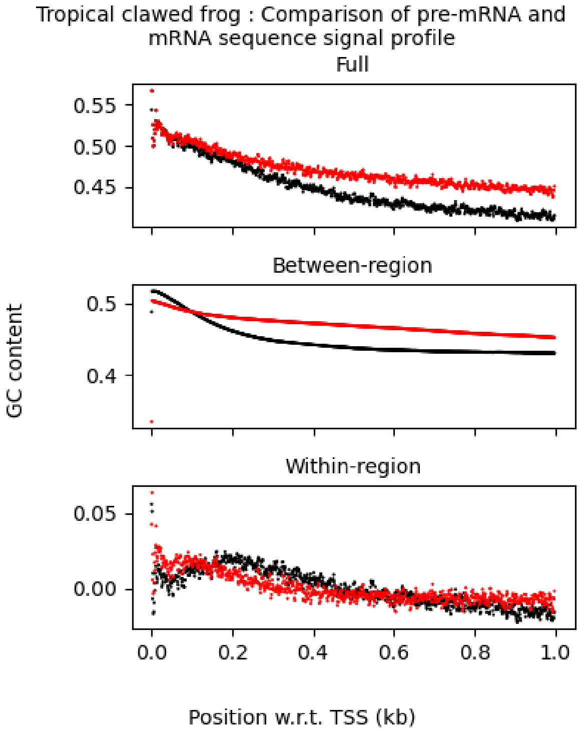
Tropical clawed frog : Comparison of pre-mRNA and mRNA sequence signal profile
Full
Between-region
Within-region
GC content
0.55
0.50
0.45
0.5
0.4
0.05
0.00
0.0 0.2 0.4 0.6 0.8 1.0
Position w.r.t. TSS (kb)

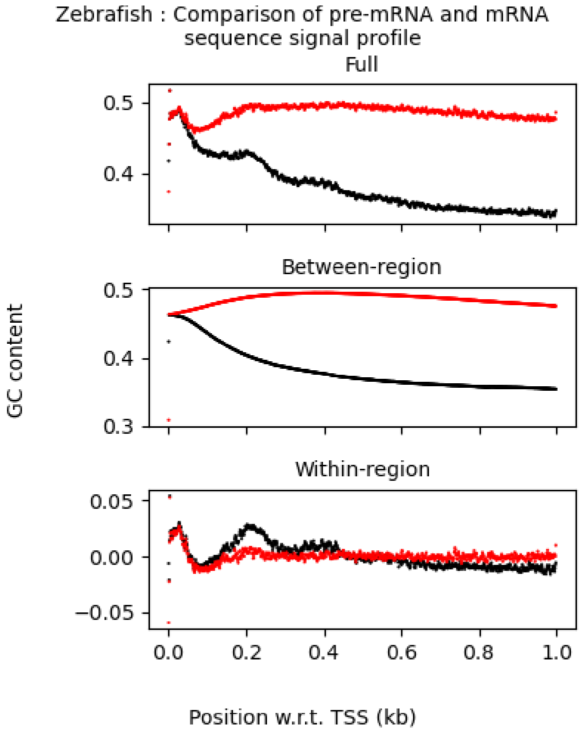
Zebrafish : Comparison of pre-mRNA and mRNA sequence signal profile
Full
Between-region
Within-region
GC content
0.5
0.4
0.3
0.05
0.00
−0.05
0.0 0.2 0.4 0.6 0.8 1.0
Position w.r.t. TSS (kb)

Plants

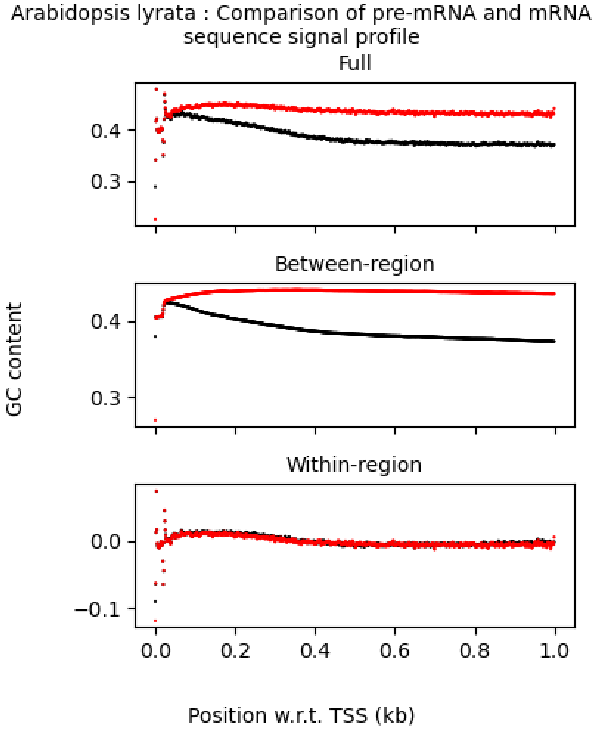
Arabidopsis lyrata : Comparison of pre-mRNA and mRNA sequence signal profile
Full
Between-region
Within-region
GC content
Position w.r.t. TSS (kb)

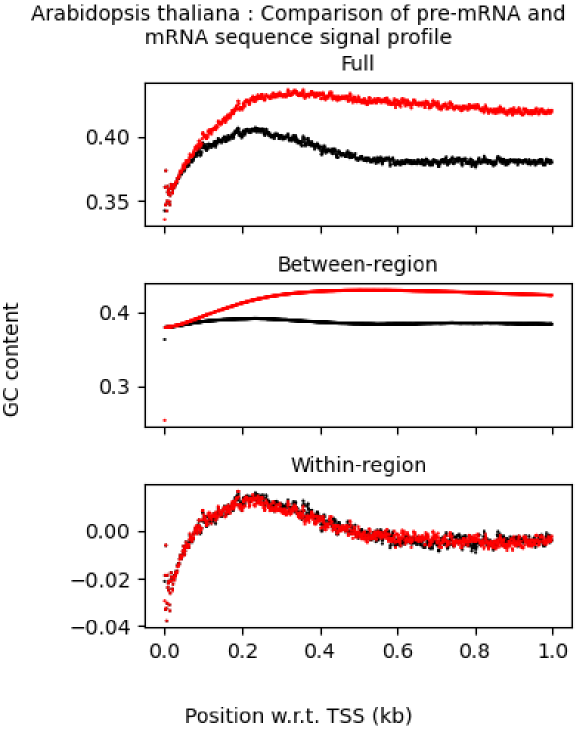
Arabidopsis thaliana : Comparison of pre-mRNA and mRNA sequence signal profile
Full
Between-region
Within-region
GC content
Position w.r.t. TSS (kb)

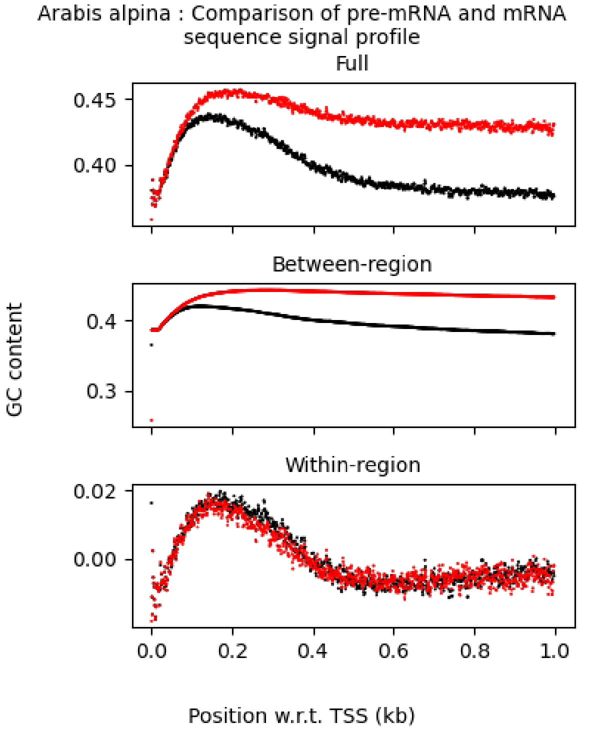
Arabis alpina : Comparison of pre-mRNA and mRNA sequence signal profile
Full
Between-region
Within-region
GC content
Position w.r.t. TSS (kb)

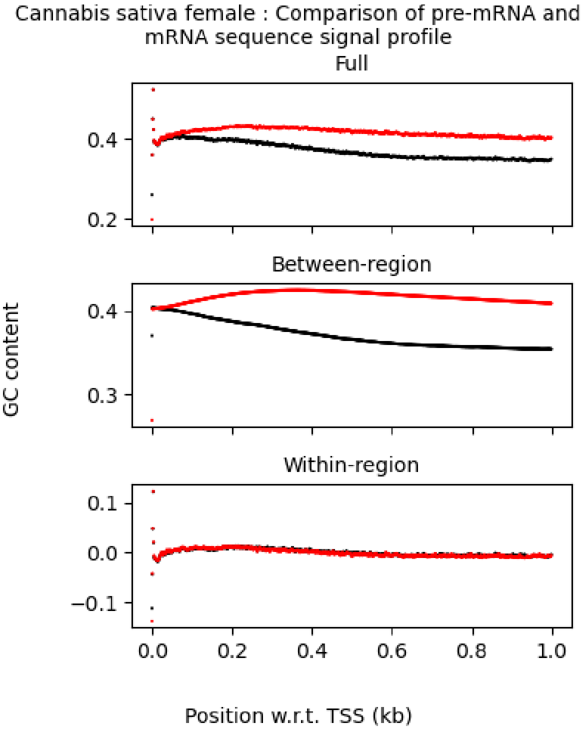
Cannabis sativa female : Comparison of pre-mRNA and mRNA sequence signal profile
Full
Between-region
Within-region
GC content
Position w.r.t. TSS (kb)

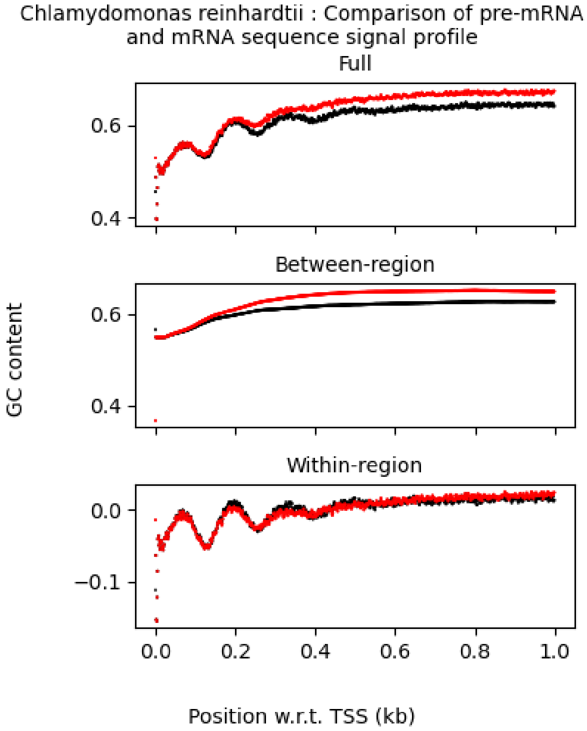
Chlamydomonas reinhardtii : Comparison of pre-mRNA and mRNA sequence signal profile
Full
Between-region
Within-region
GC content
Position w.r.t. TSS (kb)

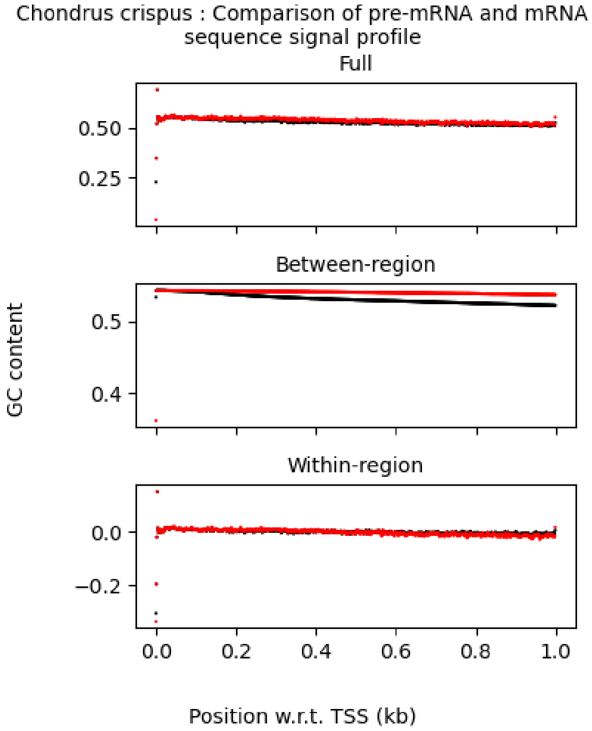
Chondrus crispus : Comparison of pre-mRNA and mRNA sequence signal profile
Full
Between-region
Within-region
GC content
Position w.r.t. TSS (kb)

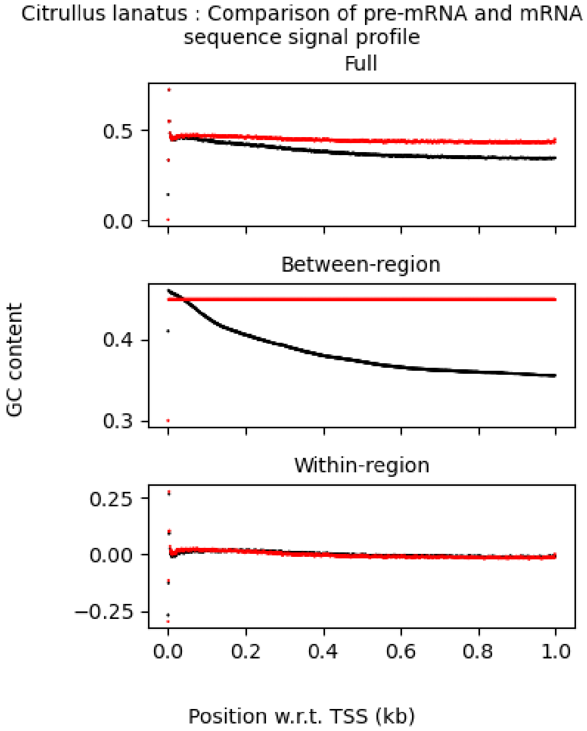
Citrullus lanatus : Comparison of pre-mRNA and mRNA sequence signal profile
Full
Between-region
Within-region
GC content
Position w.r.t. TSS (kb)

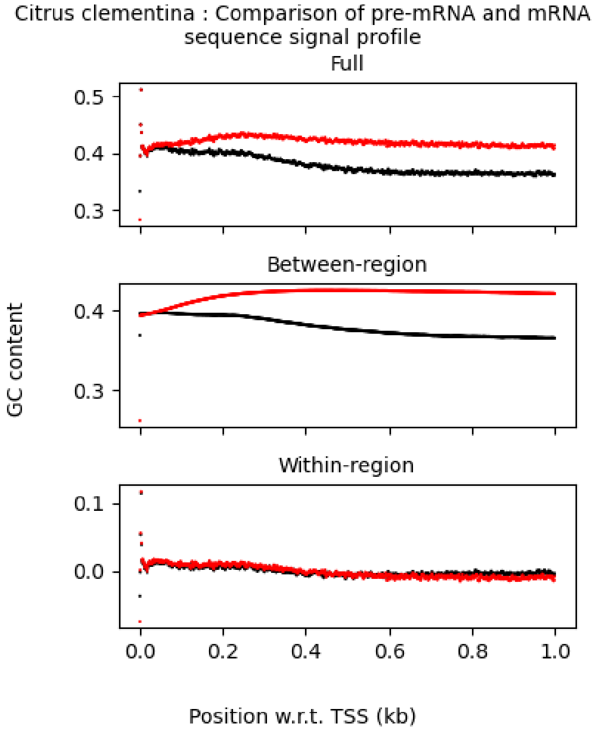
Citrus clementina : Comparison of pre-mRNA and mRNA sequence signal profile
Full
Between-region
Within-region
GC content
Position w.r.t. TSS (kb)

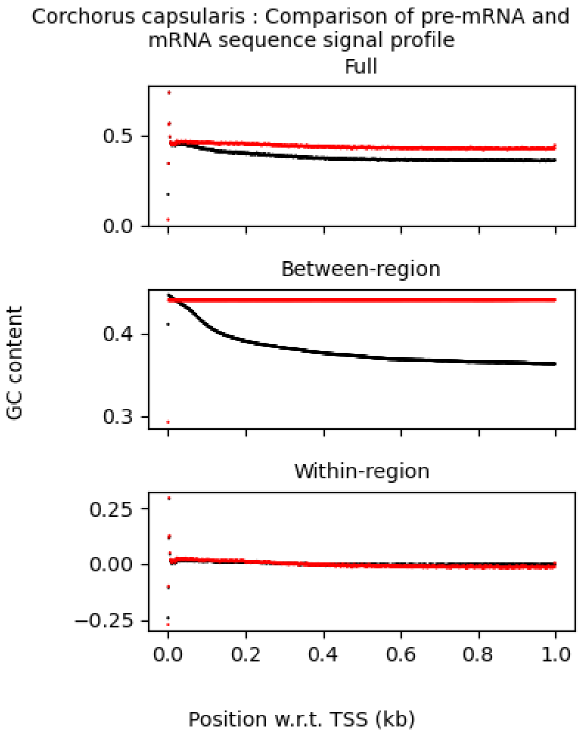
Corchorus capsularis : Comparison of pre-mRNA and mRNA sequence signal profile
Full
Between-region
Within-region
GC content
Position w.r.t. TSS (kb)

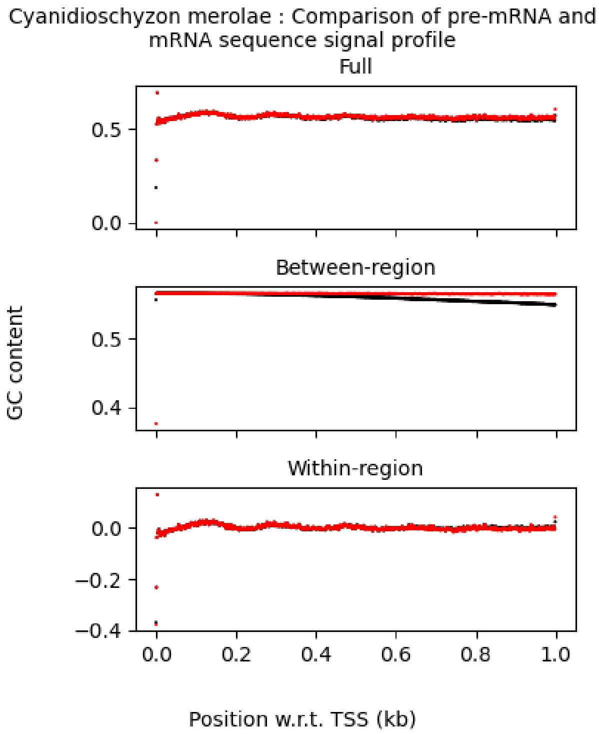
Cyanidioschyzon merolae : Comparison of pre-mRNA and mRNA sequence signal profile
Full
Between-region
Within-region
GC content
Position w.r.t. TSS (kb)

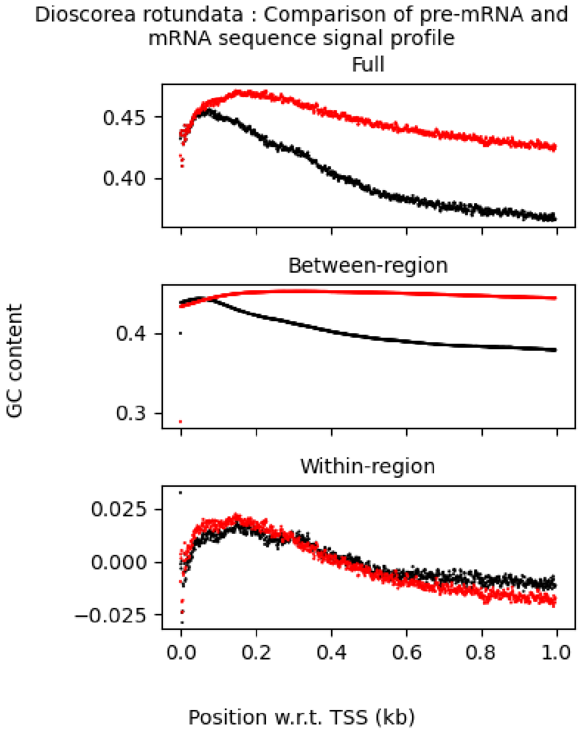
Dioscorea rotundata : Comparison of pre-mRNA and mRNA sequence signal profile
Full
Between-region
Within-region
GC content
Position w.r.t. TSS (kb)

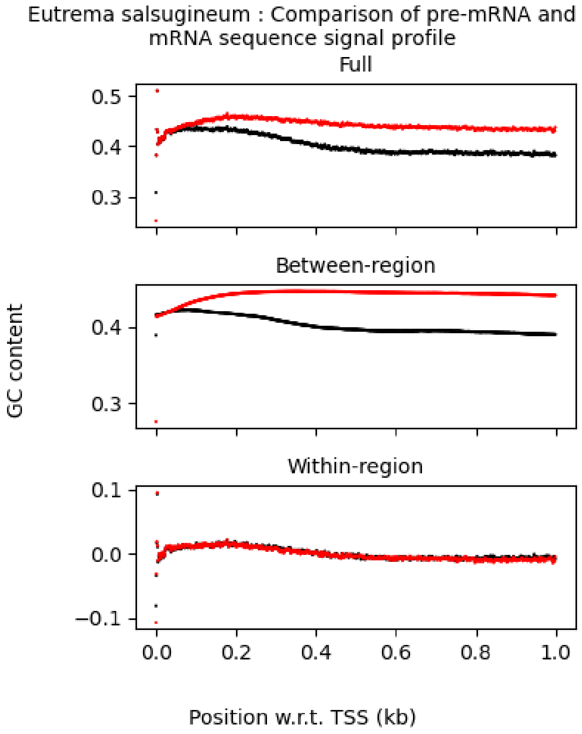
Eutrema salsugineum : Comparison of pre-mRNA and mRNA sequence signal profile
Full
Between-region
Within-region
GC content
Position w.r.t. TSS (kb)

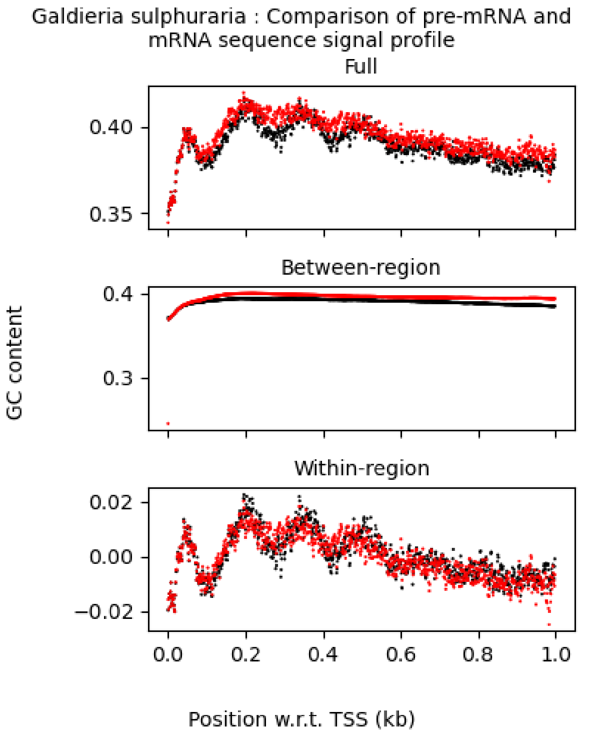
Galdieria sulphuraria : Comparison of pre-mRNA and mRNA sequence signal profile
Full
Between-region
Within-region
GC content
Position w.r.t. TSS (kb)

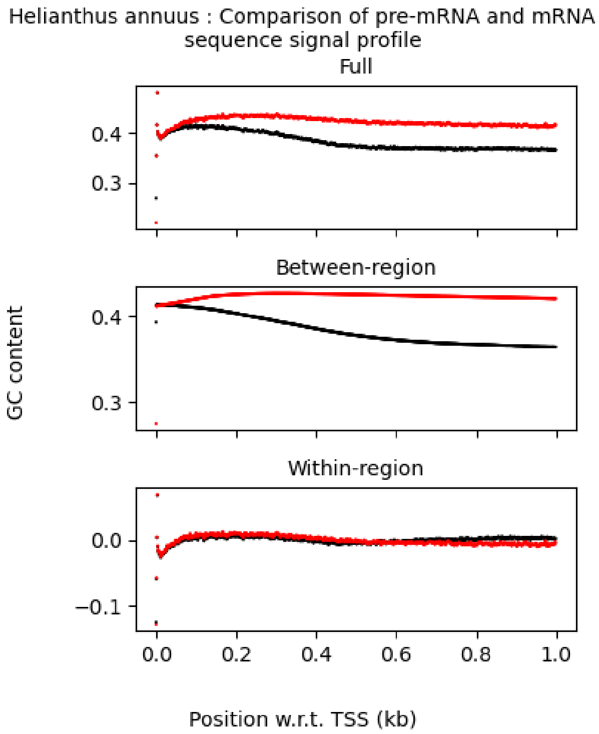
Helianthus annuus : Comparison of pre-mRNA and mRNA sequence signal profile
Full
Between-region
Within-region
GC content
Position w.r.t. TSS (kb)

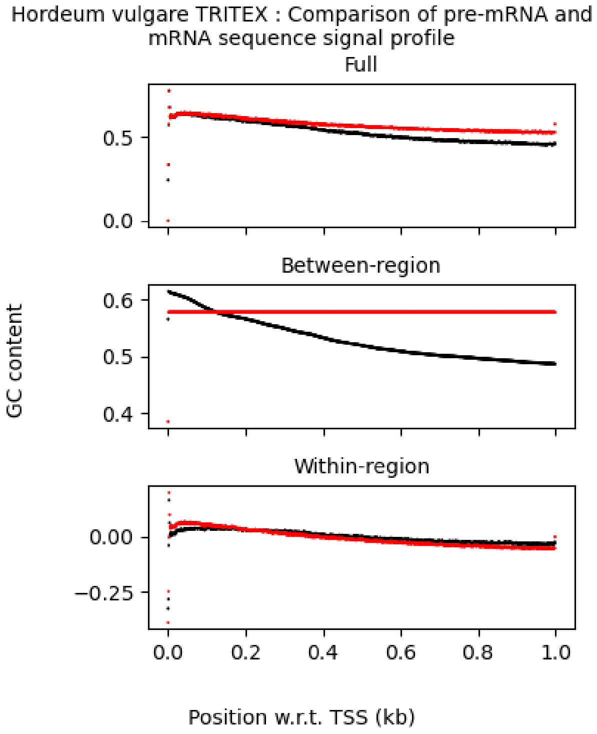
Hordeum vulgare TRITEX : Comparison of pre-mRNA and mRNA sequence signal profile
Full
Between-region
Within-region
GC content
Position w.r.t. TSS (kb)

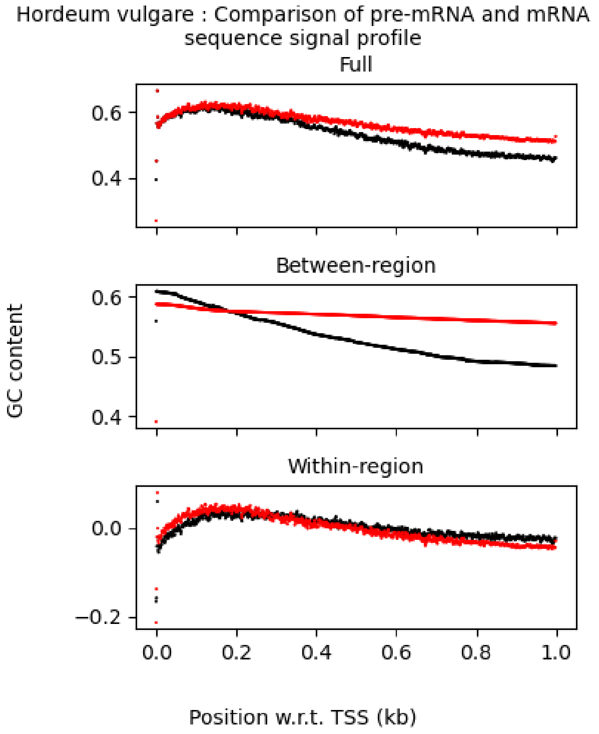
Hordeum vulgare : Comparison of pre-mRNA and mRNA sequence signal profile
Full
Between-region
Within-region
GC content
Position w.r.t. TSS (kb)

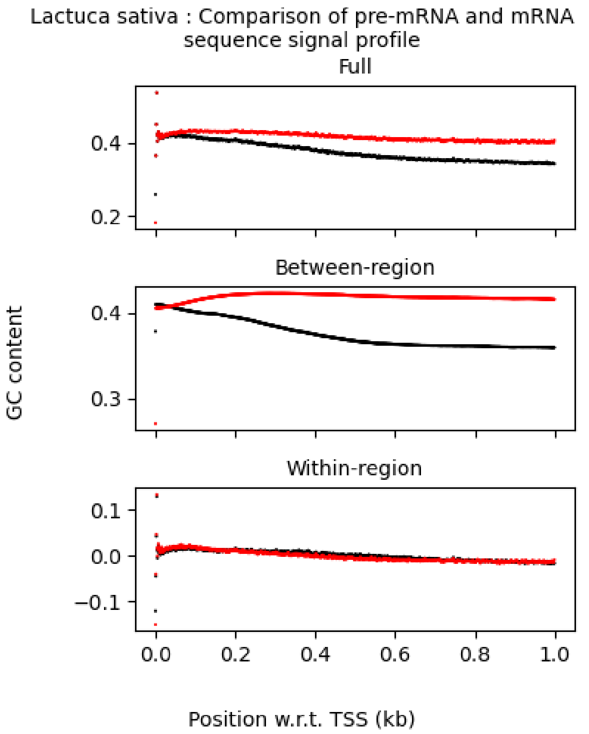
Lactuca sativa : Comparison of pre-mRNA and mRNA sequence signal profile
Full
Between-region
Within-region
GC content
0.4
0.2
0.3
0.1
0.0
−0.1
0.0
0.2
0.4
0.6
0.8
1.0
Position w.r.t. TSS (kb)

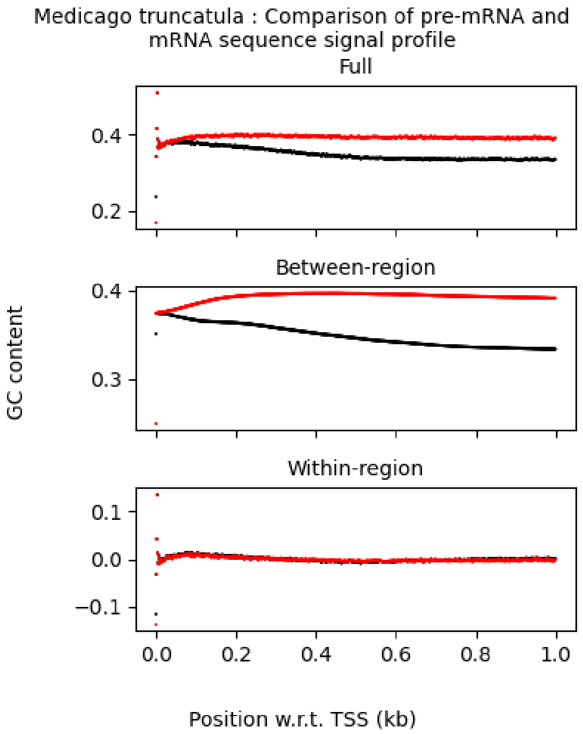
Medicago truncatula : Comparison of pre-mRNA and mRNA sequence signal profile
Full
Between-region
Within-region
GC content
0.4
0.2
0.3
0.1
0.0
−0.1
0.0
0.2
0.4
0.6
0.8
1.0
Position w.r.t. TSS (kb)

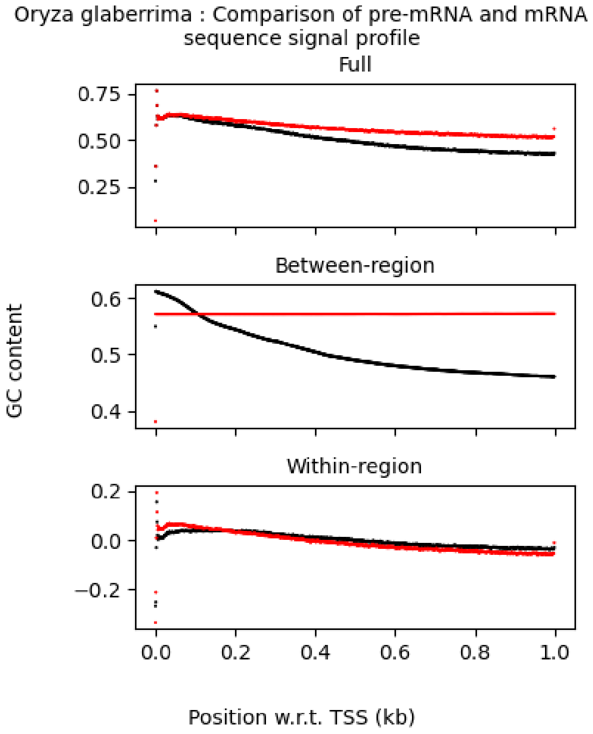
Oryza glaberrima : Comparison of pre-mRNA and mRNA sequence signal profile
Full
Between-region
Within-region
GC content
0.75
0.50
0.25
0.6
0.5
0.4
0.2
0.0
−0.2
0.0
0.2
0.4
0.6
0.8
1.0
Position w.r.t. TSS (kb)

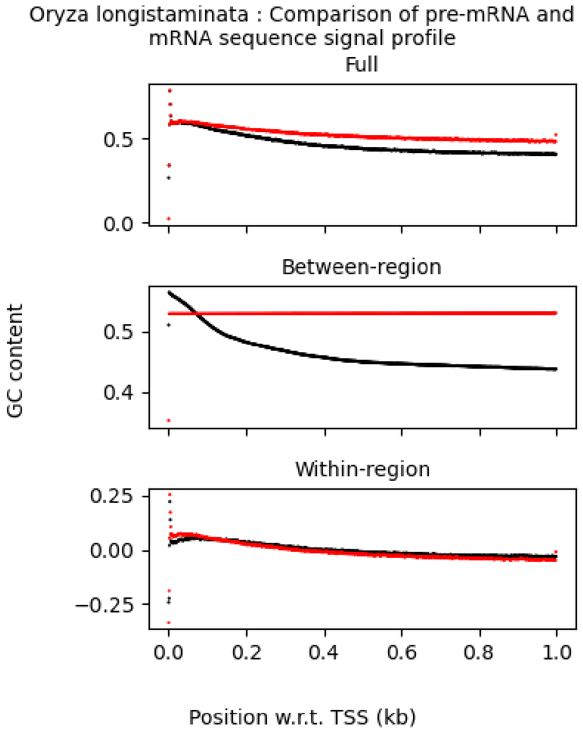
Oryza longistaminata : Comparison of pre-mRNA and mRNA sequence signal profile
Full
Between-region
Within-region
GC content
0.5
0.0
0.5
0.4
0.25
0.00
−0.25
0.0
0.2
0.4
0.6
0.8
1.0
Position w.r.t. TSS (kb)

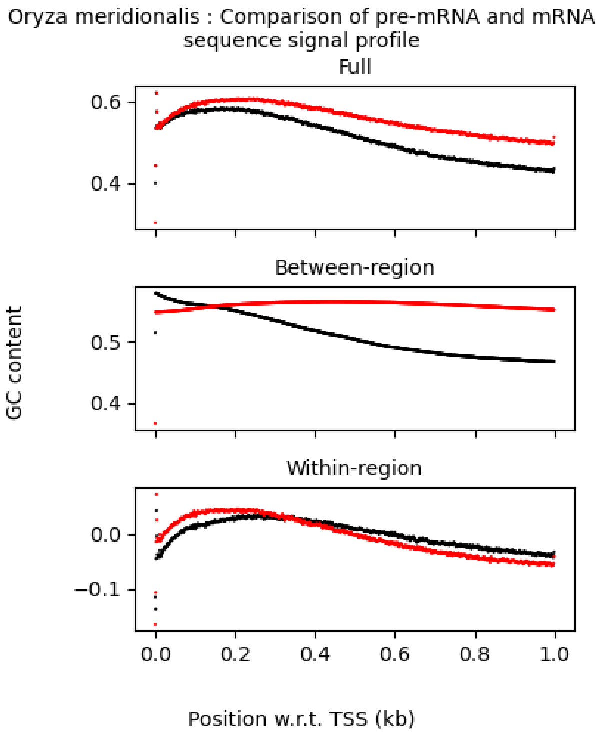
Oryza meridionalis : Comparison of pre-mRNA and mRNA sequence signal profile
Full
0.6
0.4
Between-region
0.5
0.4
GC content
Within-region
0.0
−0.1
0.0
0.2
0.4
0.6
0.8
1.0
Position w.r.t. TSS (kb)

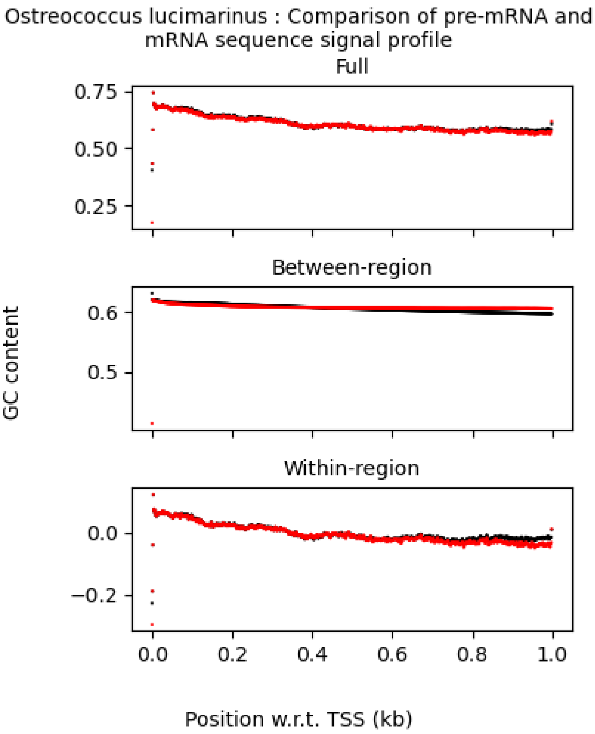
Ostreococcus lucimarinus : Comparison of pre-mRNA and mRNA sequence signal profile
Full
0.75
0.50
0.25
Between-region
0.6
0.5
GC content
Within-region
0.0
−0.2
0.0
0.2
0.4
0.6
0.8
1.0
Position w.r.t. TSS (kb)

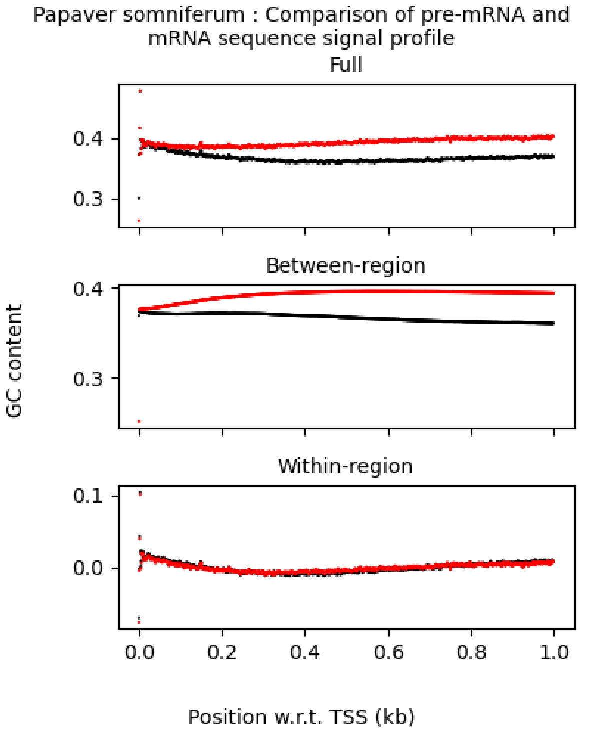
Papaver somniferum : Comparison of pre-mRNA and mRNA sequence signal profile
Full
0.4
0.3
Between-region
0.4
0.3
GC content
Within-region
0.1
0.0
0.0
0.2
0.4
0.6
0.8
1.0
Position w.r.t. TSS (kb)

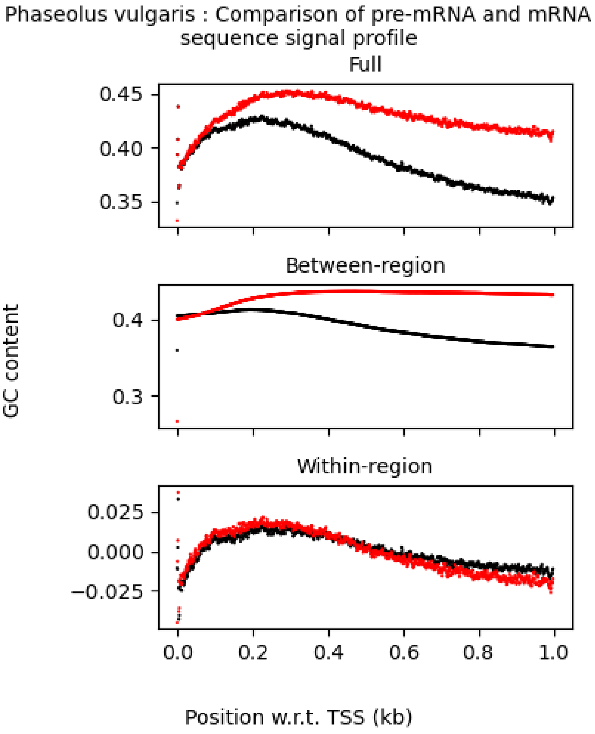
Phaseolus vulgaris : Comparison of pre-mRNA and mRNA sequence signal profile
Full
0.45
0.40
0.35
Between-region
0.4
0.3
GC content
Within-region
0.025
0.000
−0.025
0.0
0.2
0.4
0.6
0.8
1.0
Position w.r.t. TSS (kb)

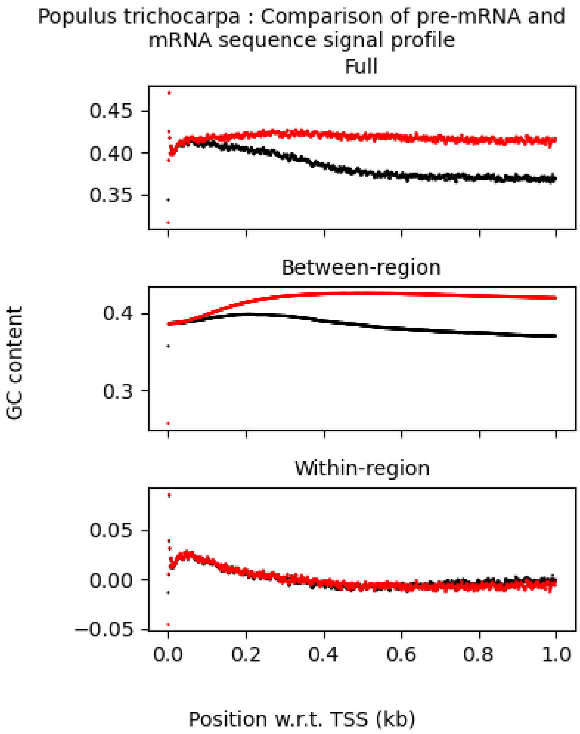
Populus trichocarpa : Comparison of pre-mRNA and mRNA sequence signal profile
Full
0.45
0.40
0.35
Between-region
0.4
0.3
Within-region
0.05
0.00
−0.05
GC content
0.0
0.2
0.4
0.6
0.8
1.0
Position w.r.t. TSS (kb)

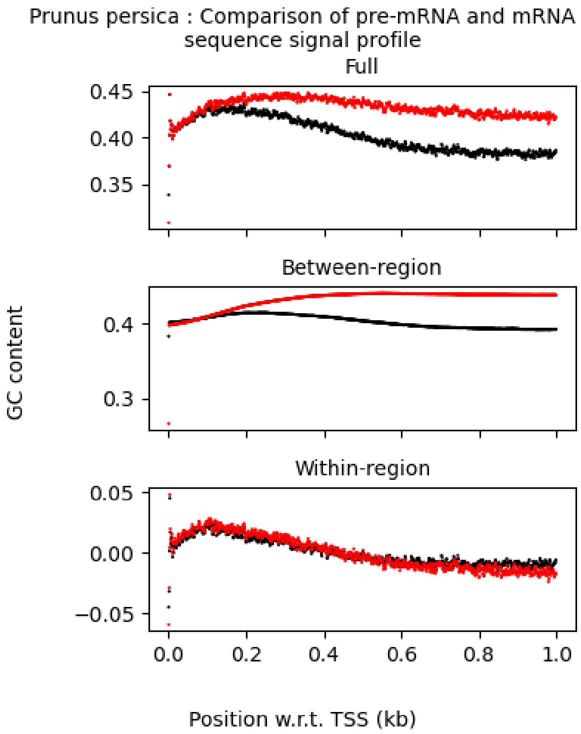
Prunus persica : Comparison of pre-mRNA and mRNA sequence signal profile
Full
0.45
0.40
0.35
Between-region
0.4
0.3
Within-region
0.05
0.00
−0.05
GC content
0.0
0.2
0.4
0.6
0.8
1.0
Position w.r.t. TSS (kb)

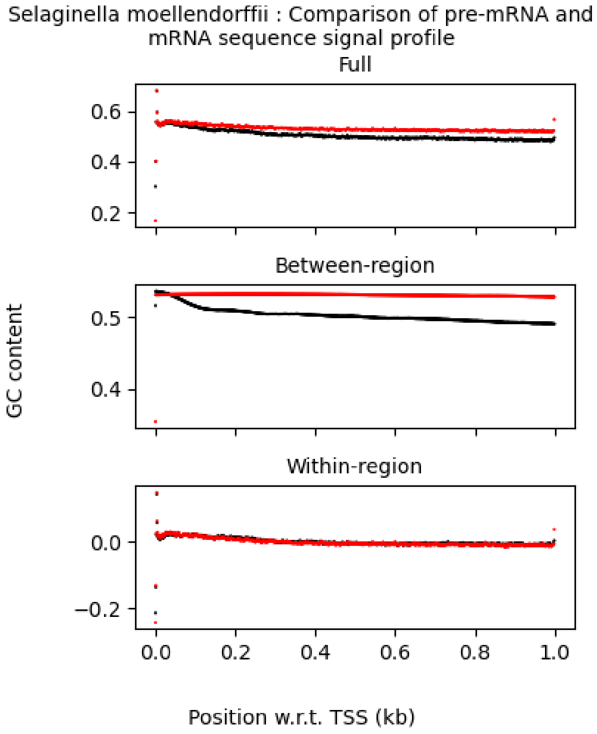
Selaginella moellendorffii : Comparison of pre-mRNA and mRNA sequence signal profile
Full
0.6
0.4
0.2
Between-region
0.5
0.4
Within-region
0.0
−0.2
GC content
0.0
0.2
0.4
0.6
0.8
1.0
Position w.r.t. TSS (kb)

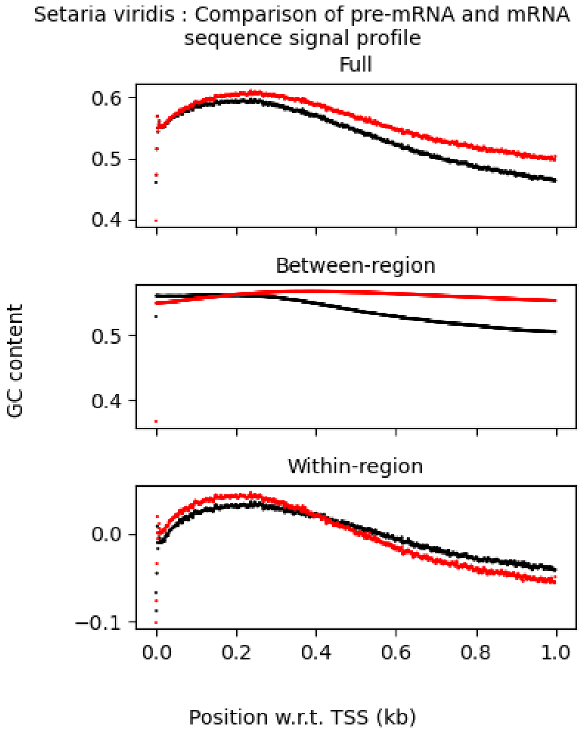
Setaria viridis : Comparison of pre-mRNA and mRNA sequence signal profile
Full
0.6
0.5
0.4
Between-region
0.5
0.4
Within-region
0.0
−0.1
GC content
0.0
0.2
0.4
0.6
0.8
1.0
Position w.r.t. TSS (kb)

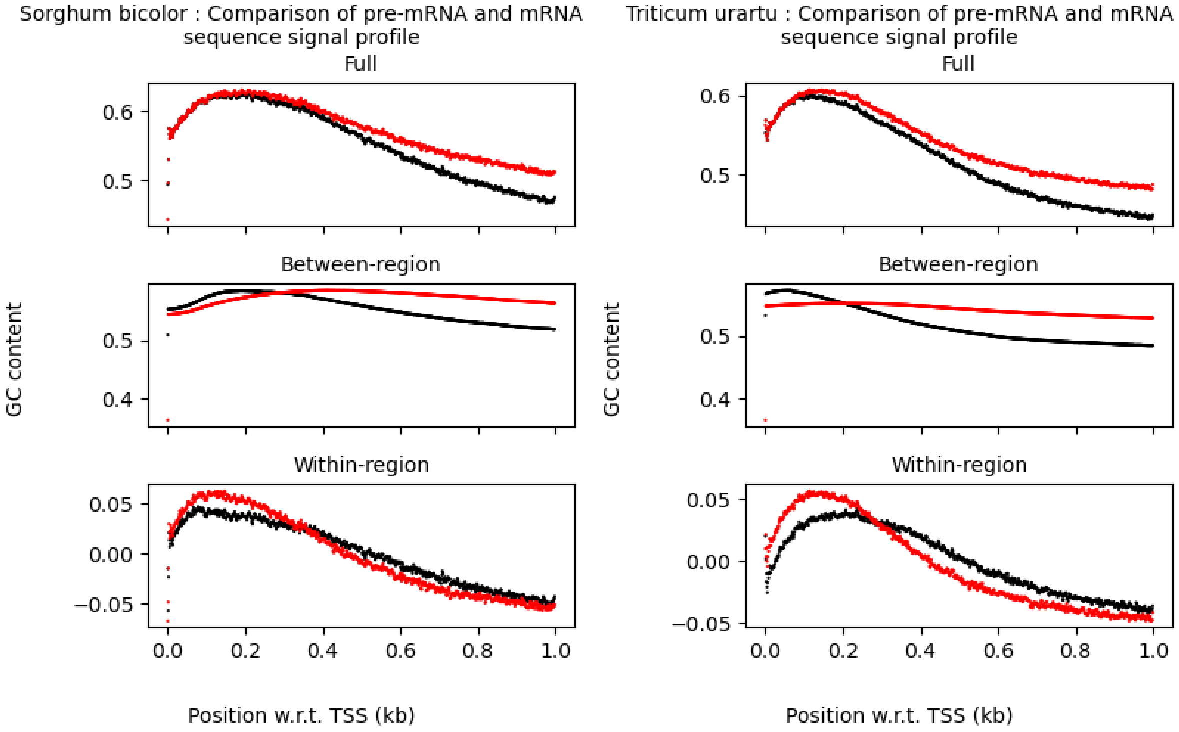
Sorghum bicolor : Comparison of pre-mRNA and mRNA sequence signal profile
Full
Between-region
Within-region
GC content
Position w.r.t. TSS (kb)
0.0
0.2
0.4
0.6
0.8
1.0
0.6
0.5
0.4
0.05
0.00
−0.05
Triticum urartu : Comparison of pre-mRNA and mRNA sequence signal profile
Full
Between-region
Within-region
GC content
Position w.r.t. TSS (kb)

Figure S7

The heatmaps show the signal size comparison between DNA and mRNA sequences downstream of TSS for 211 vertebrate species and 118 plant species. The bars on the left side of the heatmap denote the taxonomic class of the organism. The taxonomic class distribution for vertebrates and plants is available in Supplementary Table 2. Due to the constraint on the size of the heatmap, not all species names could be shown. The calculation of the signal size is defined in the Methods section.

Vertebrates: Full GC signal

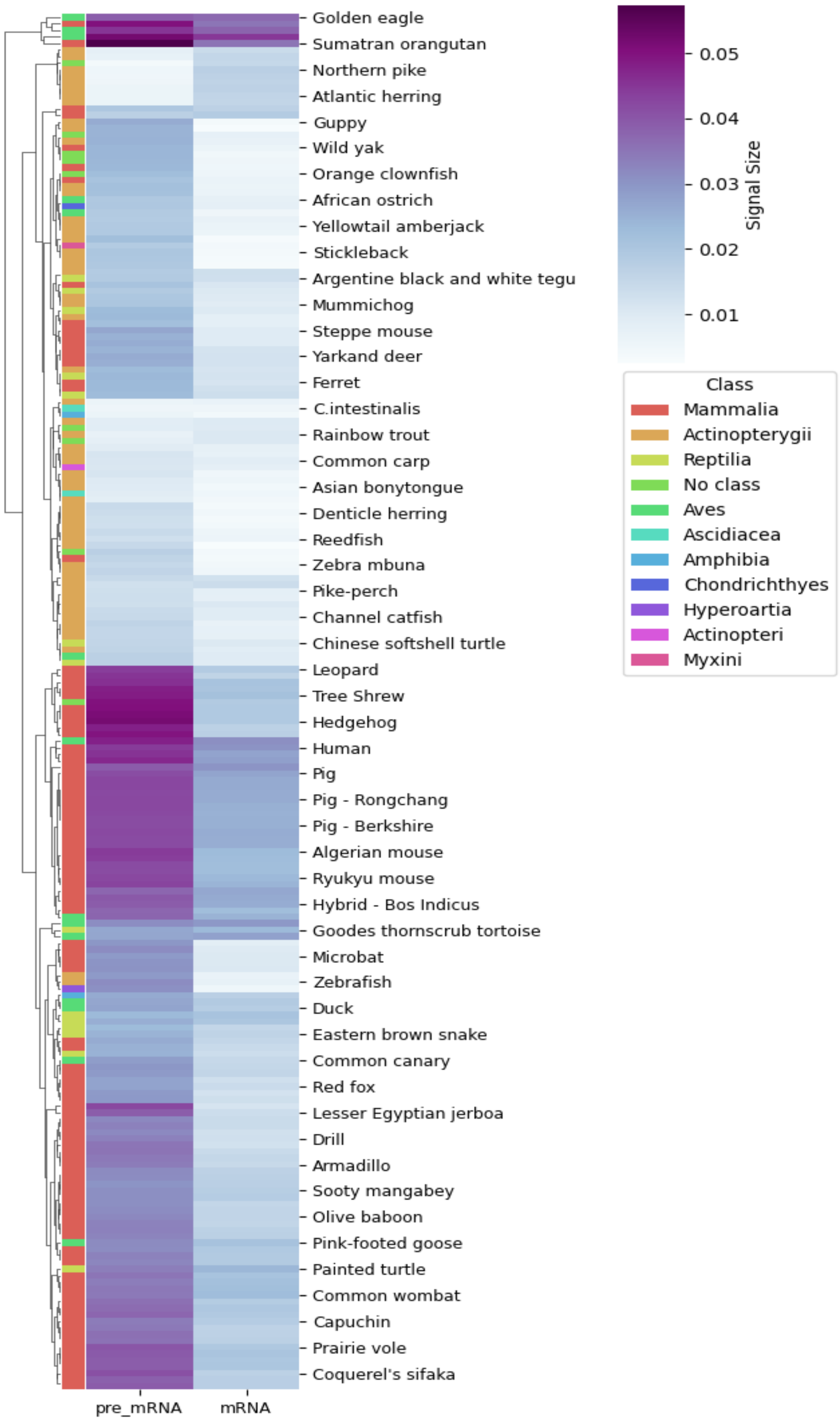

Vertebrates: Between-region signal

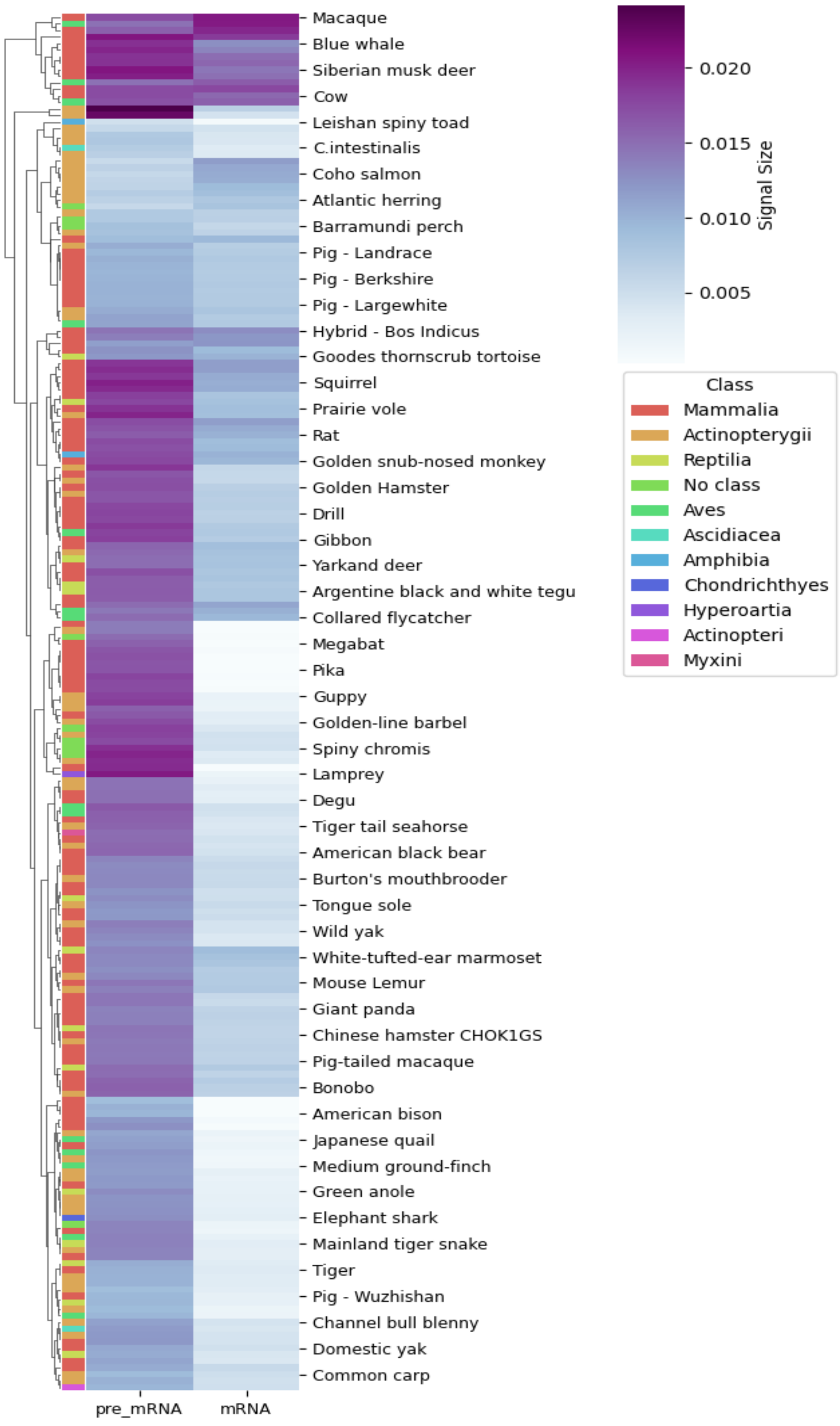

Vertebrates: Within-region signal

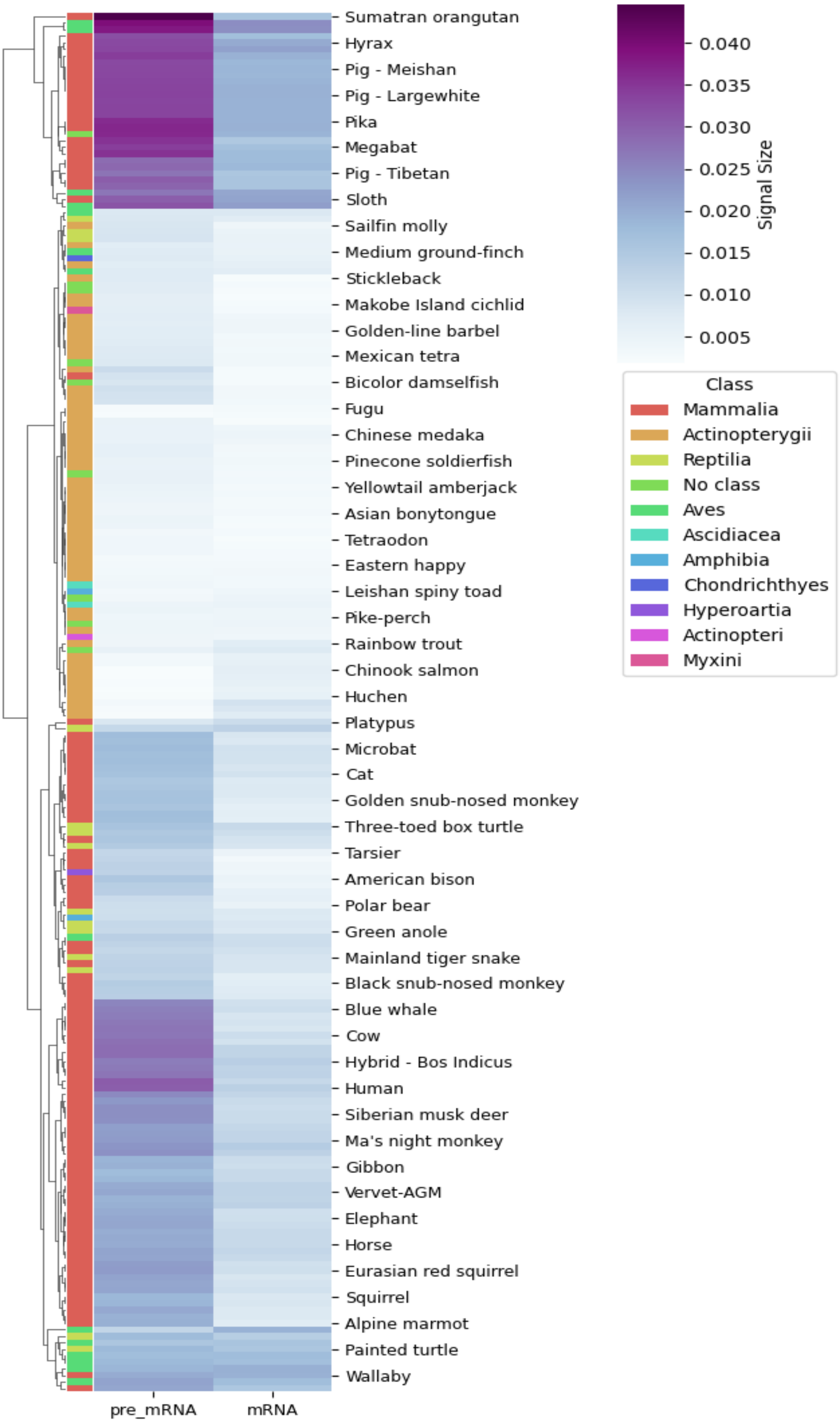

Plants: Full GC signal

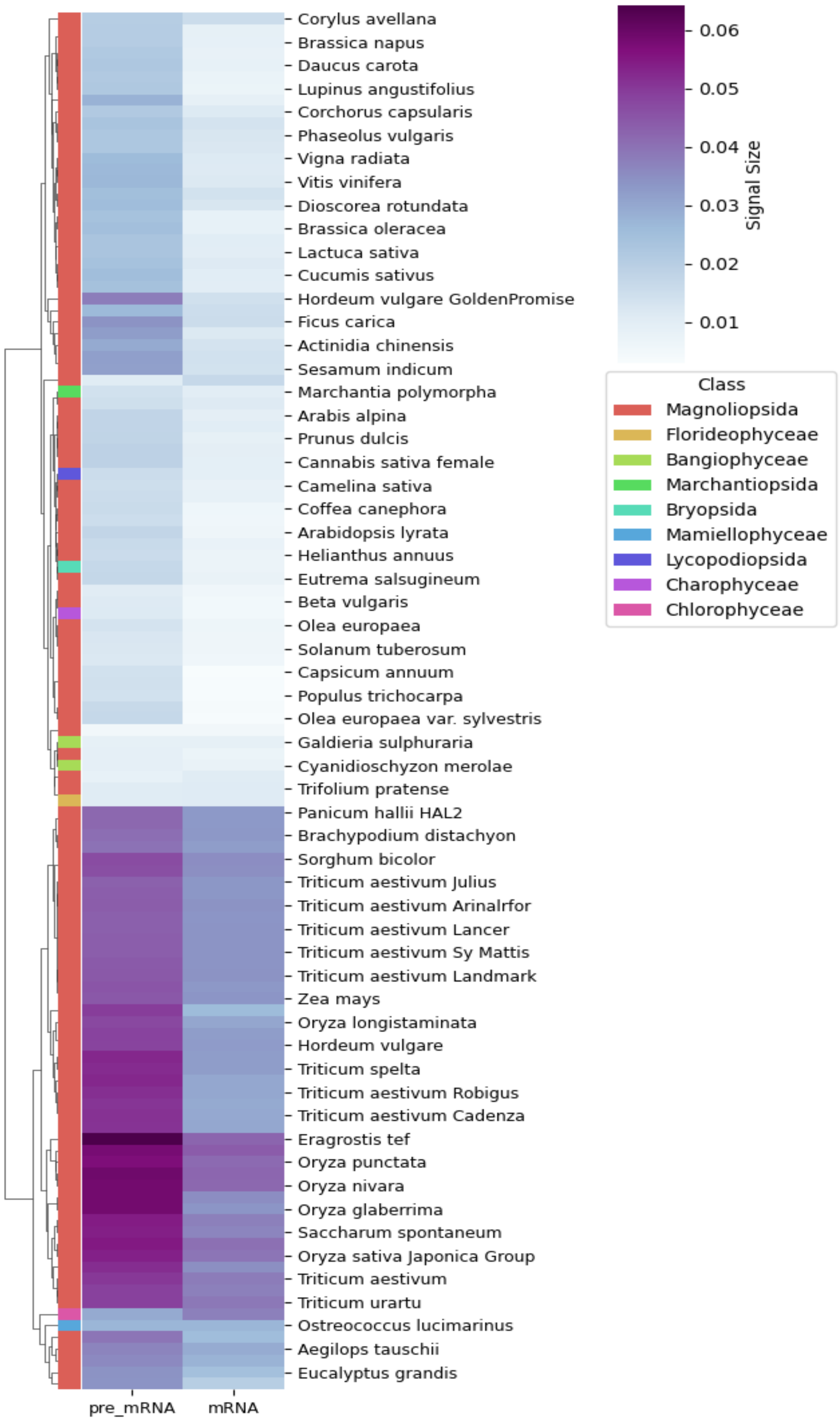

Plants: Between-region signal

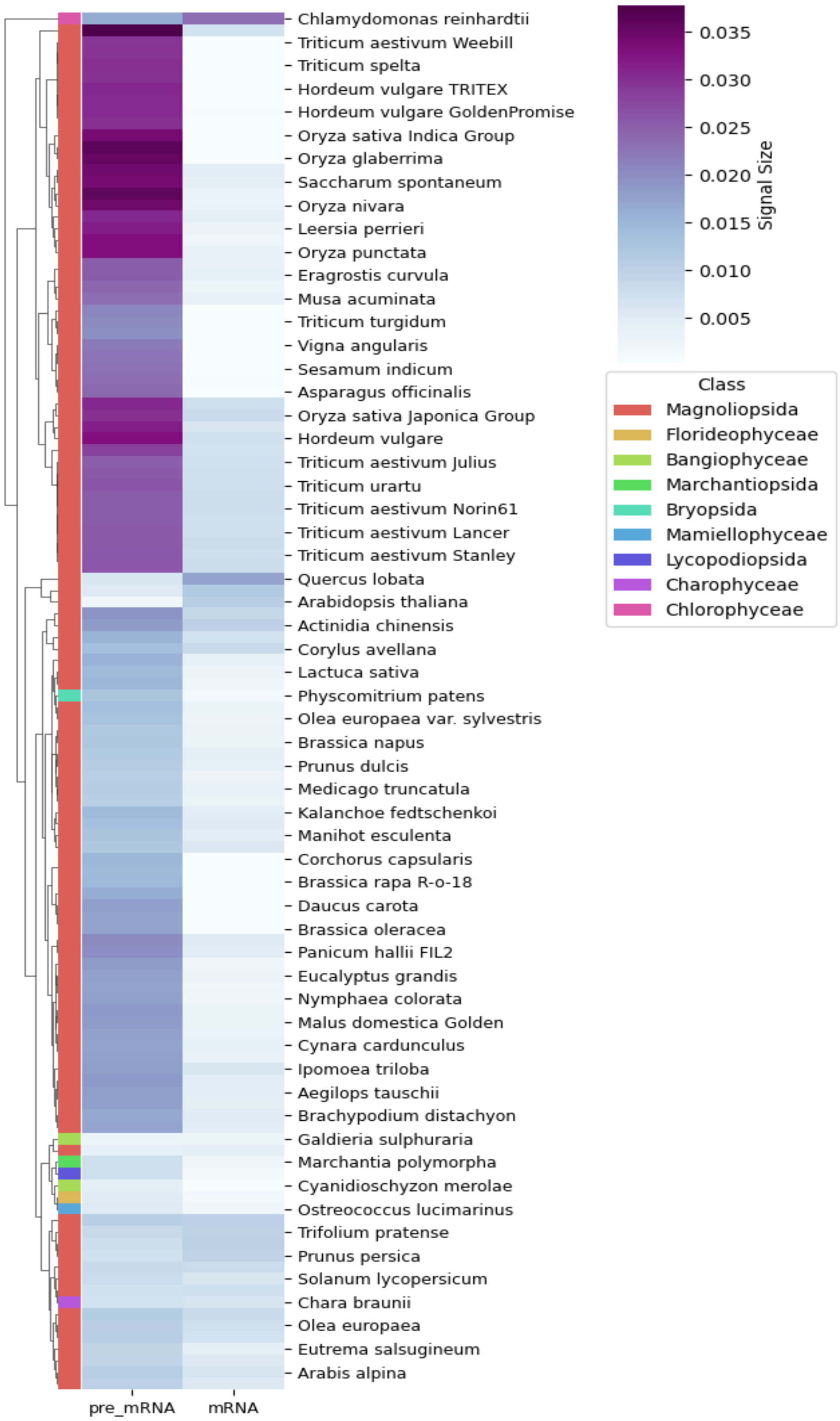

Plants: Within-region signal

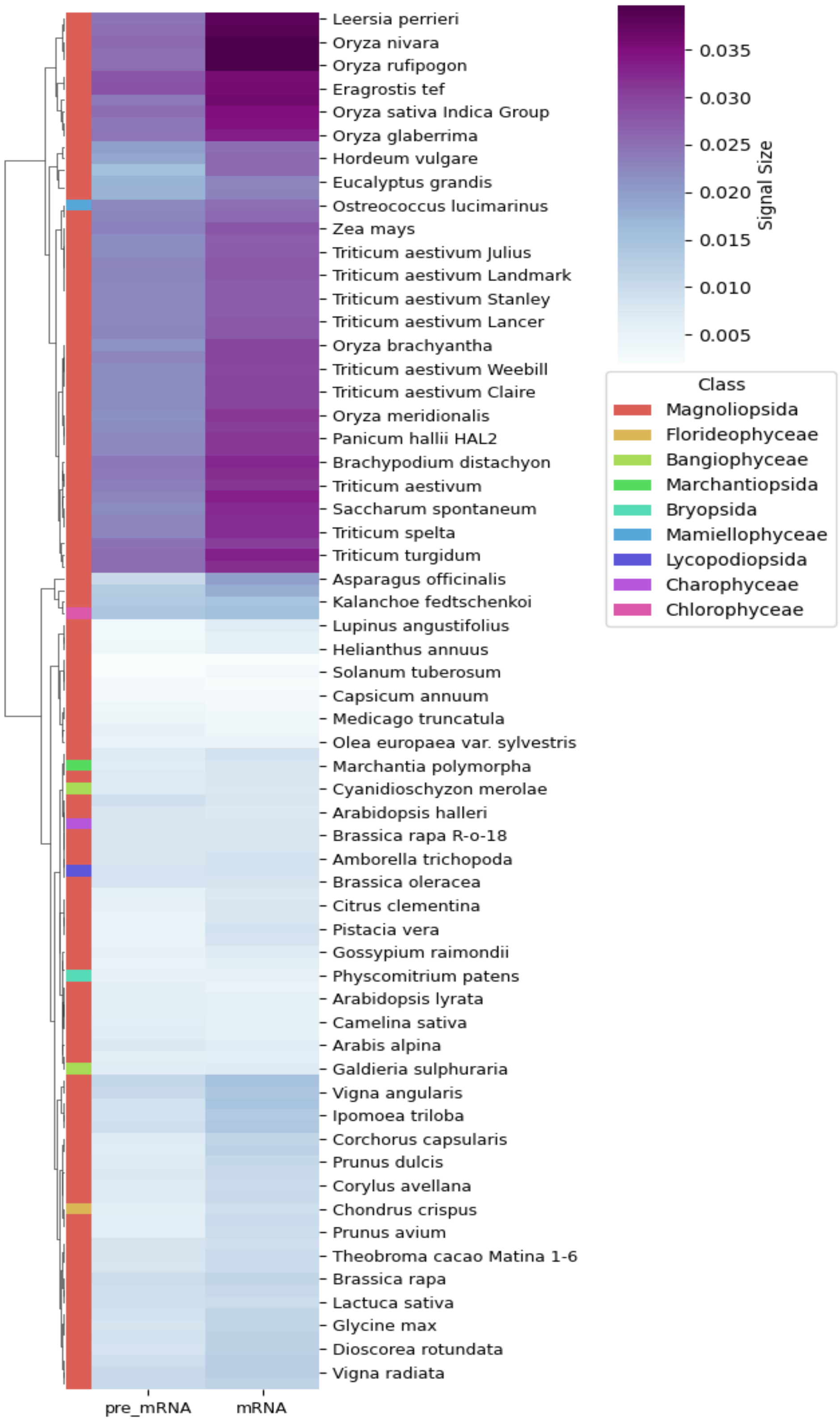

Figure S8

Coding exon within-region signal (black) and its two components, amino acid choice (blue) and synonymous codon bias (red) for selected vertebrates and plants. Data points with z-scores greater than 5 were excluded from the plot.

Vertebrates

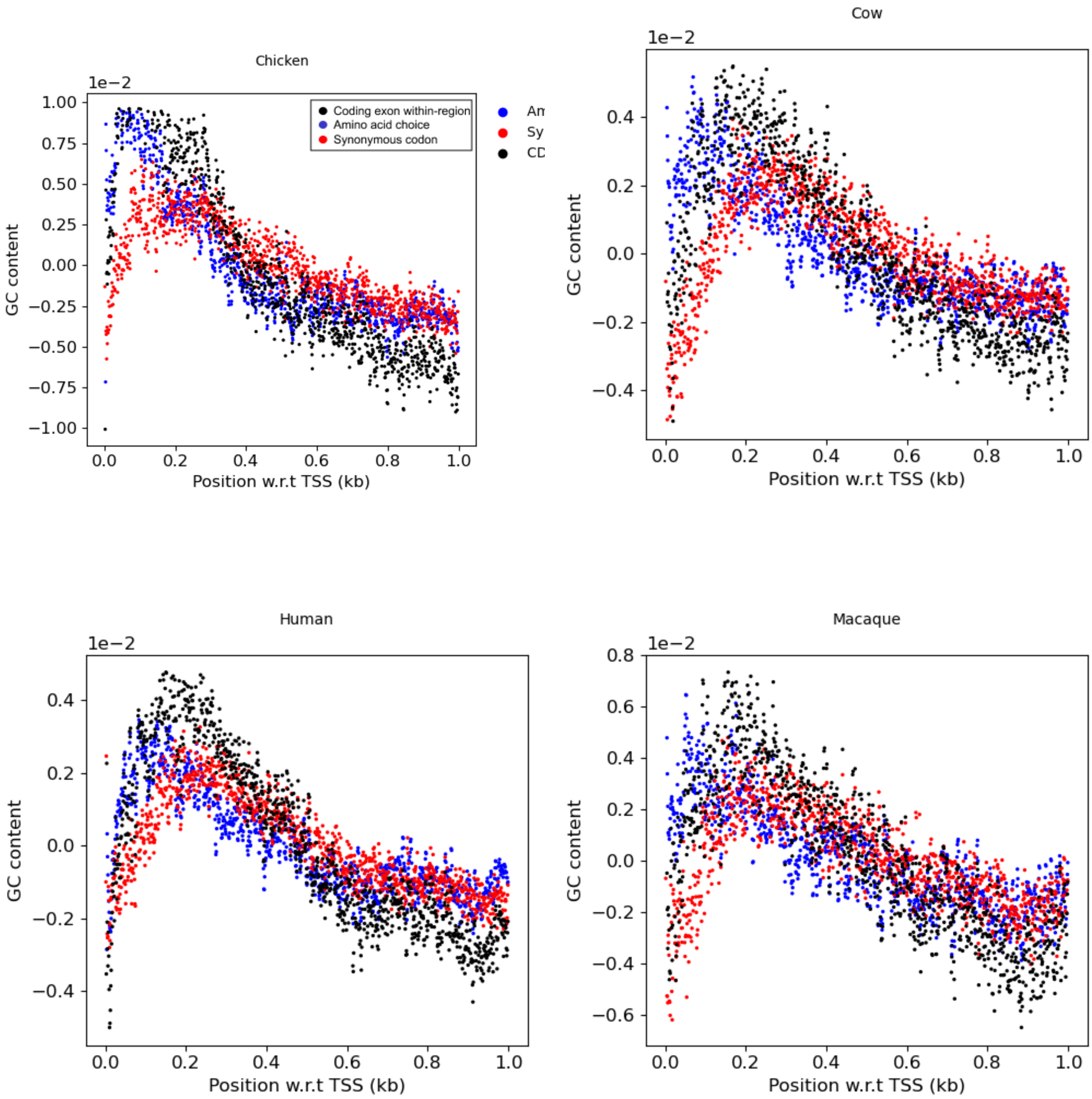

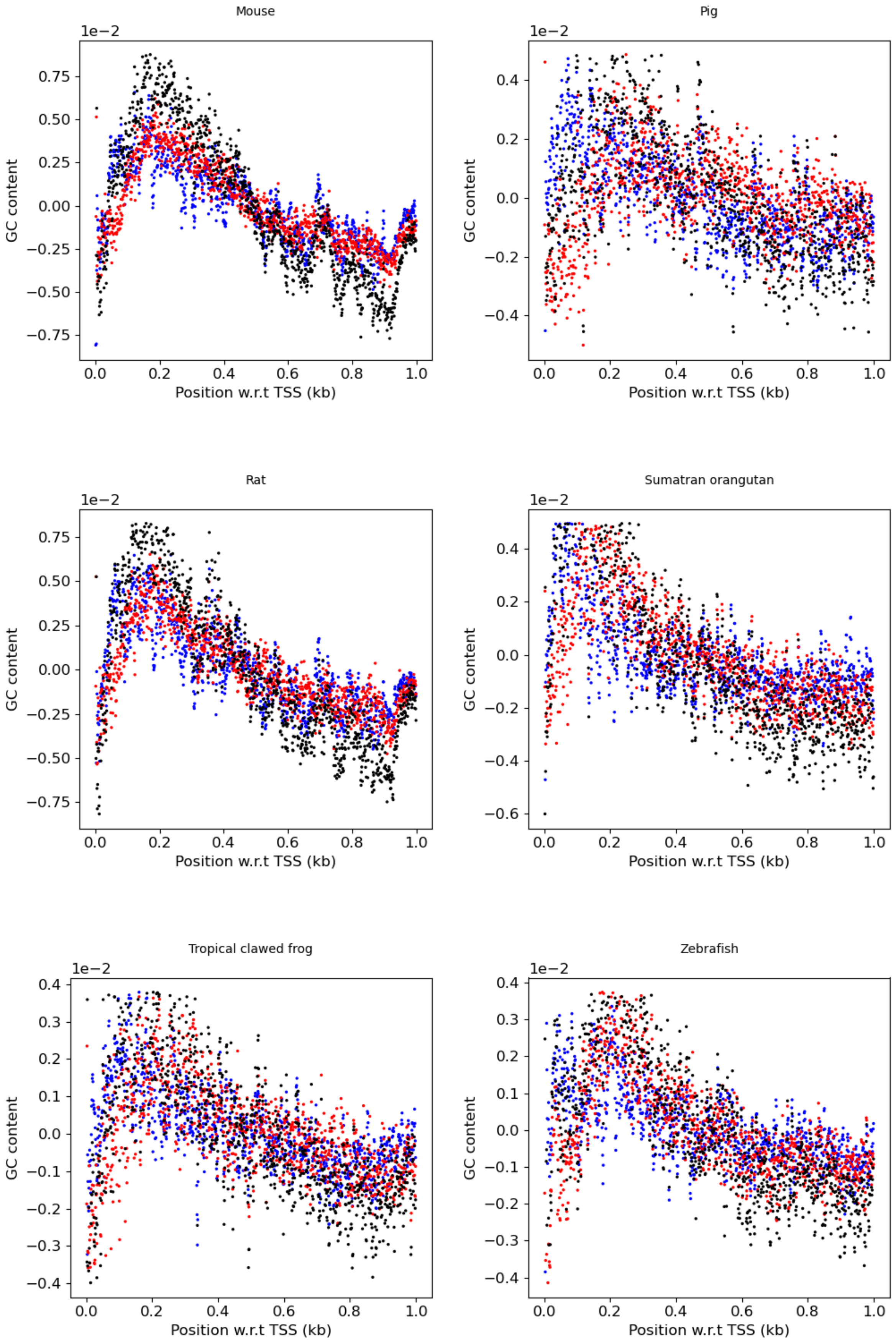
Mouse
1e−2
GC content
Position w.r.t TSS (kb)
Pig
1e−2
GC content
Position w.r.t TSS (kb)
Rat
1e−2
GC content
Position w.r.t TSS (kb)
Sumatran orangutan
1e−2
GC content
Position w.r.t TSS (kb)
Tropical clawed frog
1e−2
GC content
Position w.r.t TSS (kb)
Zebrafish
1e−2
GC content
Position w.r.t TSS (kb)

Plants

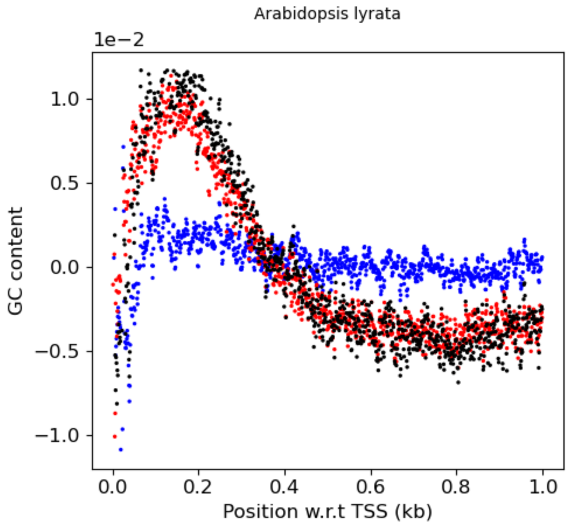

Arabidopsis lyrata
1e−2
1.0
0.5
0.0
−0.5
−1.0
GC content
0.0
0.2
0.4
0.6
0.8
1.0
Position w.r.t TSS (kb)

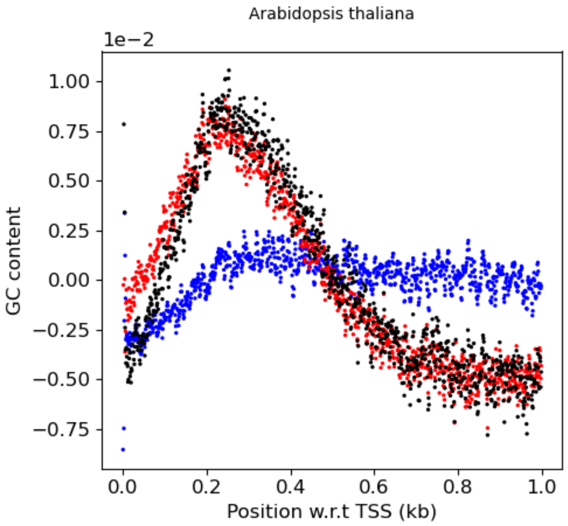

Arabidopsis thaliana
1e−2
1.00
0.75
0.50
0.25
0.00
−0.25
−0.50
−0.75
GC content
0.0
0.2
0.4
0.6
0.8
1.0
Position w.r.t TSS (kb)

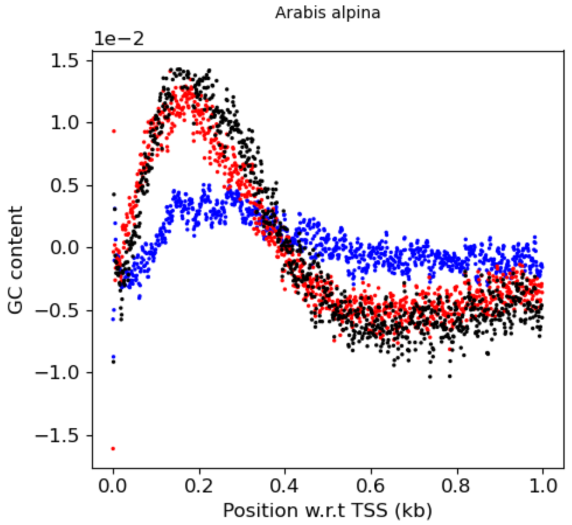

Arabis alpina
1e−2
1.5
1.0
0.5
0.0
−0.5
−1.0
−1.5
GC content
0.0
0.2
0.4
0.6
0.8
1.0
Position w.r.t TSS (kb)

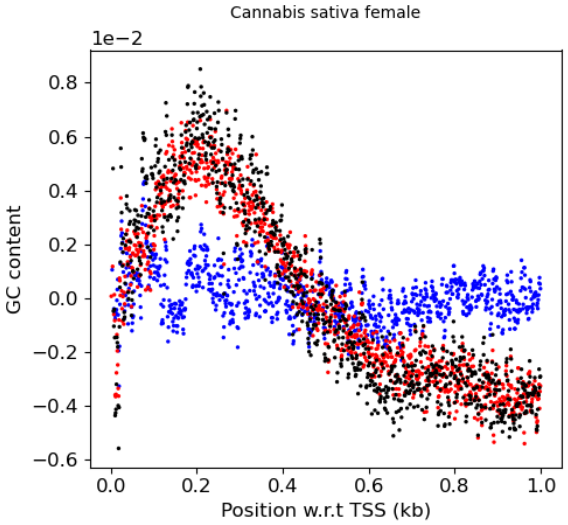

Cannabis sativa female
1e−2
0.8
0.6
0.4
0.2
0.0
−0.2
−0.4
−0.6
GC content
0.0
0.2
0.4
0.6
0.8
1.0
Position w.r.t TSS (kb)

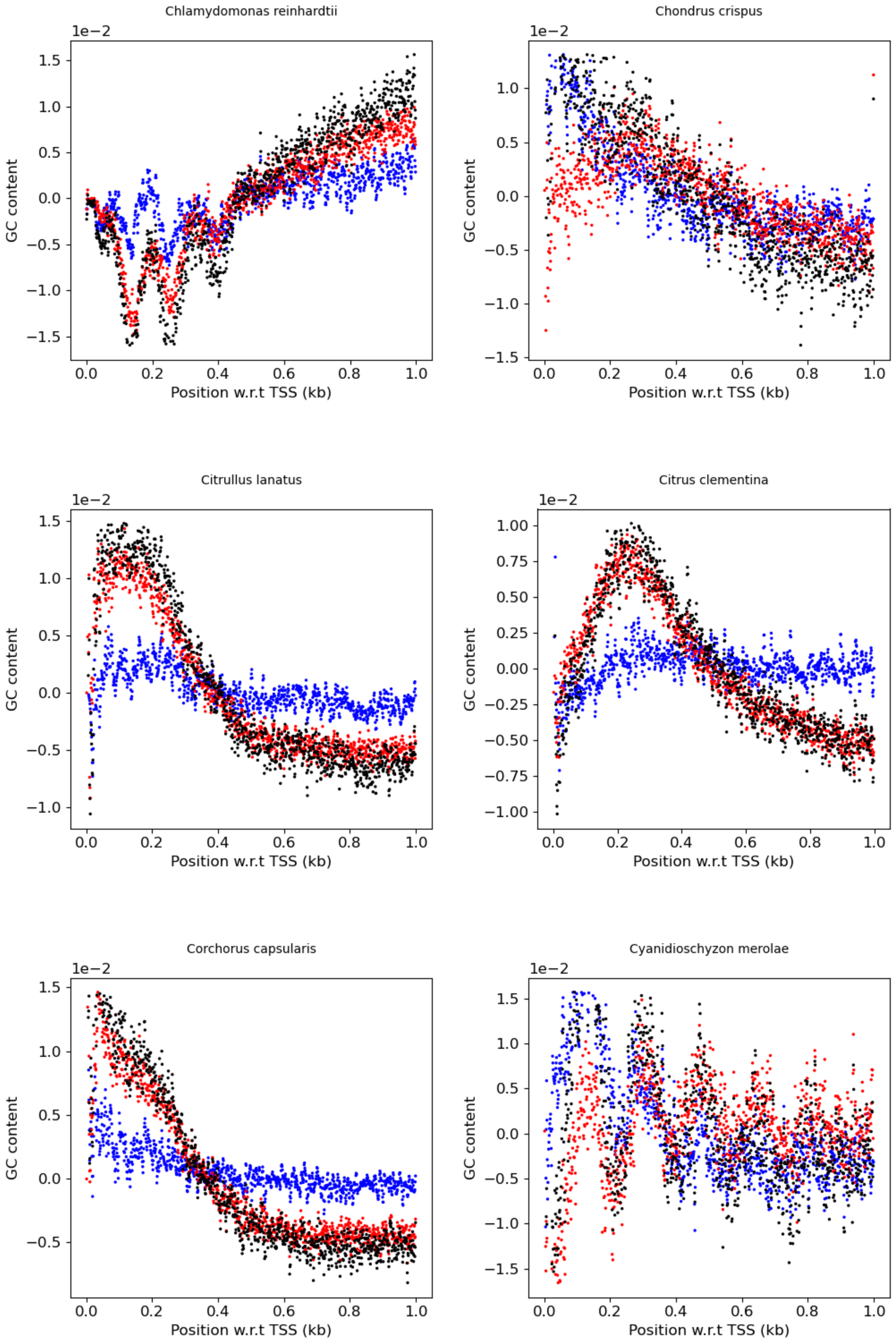
Chlamydomonas reinhardtii
1e−2
1.5
1.0
0.5
0.0
−0.5
−1.0
−1.5
GC content
0.0
0.2
0.4
0.6
0.8
1.0
Position w.r.t TSS (kb)
Chondrus crispus
1e−2
1.0
0.5
0.0
−0.5
−1.0
−1.5
GC content
Position w.r.t TSS (kb)
Citrullus lanatus
1e−2
GC content
Position w.r.t TSS (kb)
Citrus clementina
1e−2
1.00
0.75
0.50
0.25
0.00
−0.25
−0.50
−0.75
−1.00
GC content
Position w.r.t TSS (kb)
Corchorus capsularis
1e−2
GC content
Position w.r.t TSS (kb)
Cyanidioschyzon merolae
1e−2
GC content
Position w.r.t TSS (kb)

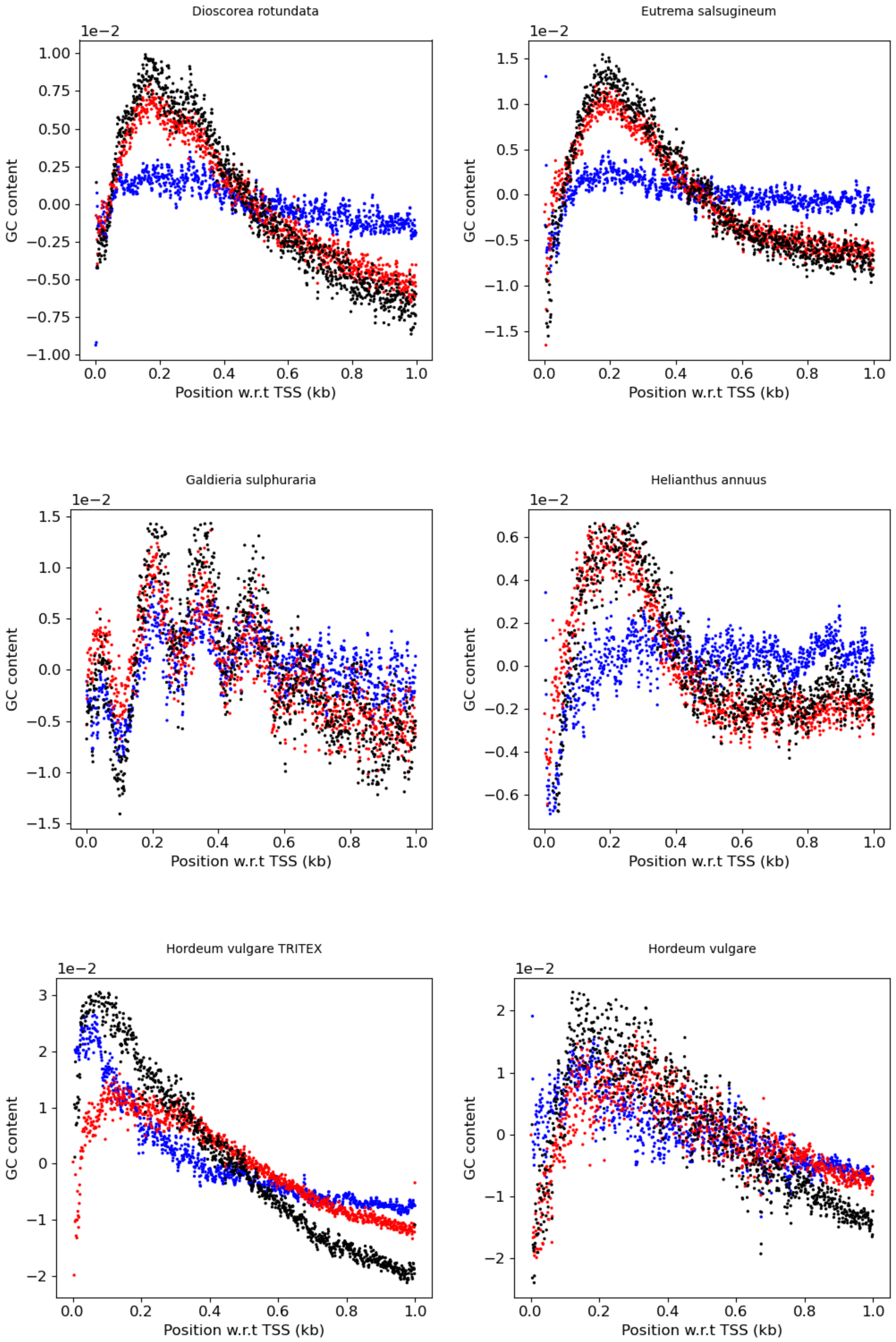
Dioscorea rotundata
Eutrema salsugineum
Galdieria sulphuraria
Helianthus annuus
Hordeum vulgare TRITEX
Hordeum vulgare
1e−2
GC content
Position w.r.t TSS (kb)

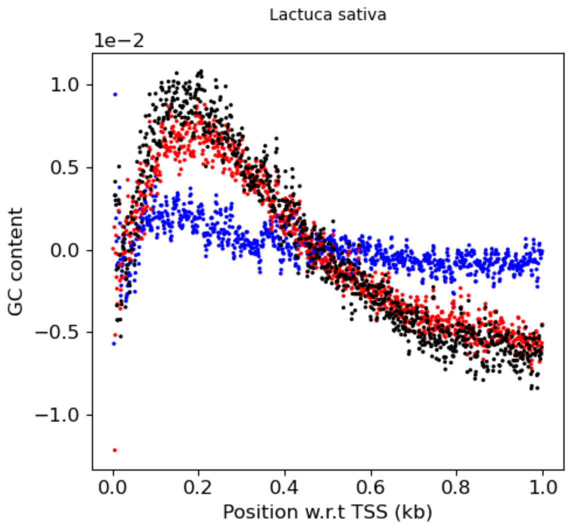
Lactuca sativa
1e−2
1.0
0.5
0.0
−0.5
−1.0
GC content
0.0 0.2 0.4 0.6 0.8 1.0
Position w.r.t TSS (kb)

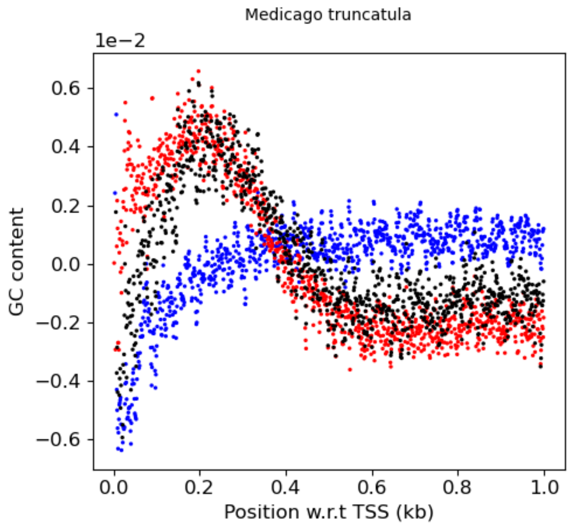
Medicago truncatula
1e−2
0.6
0.4
0.2
0.0
−0.2
−0.4
−0.6
GC content
0.0 0.2 0.4 0.6 0.8 1.0
Position w.r.t TSS (kb)

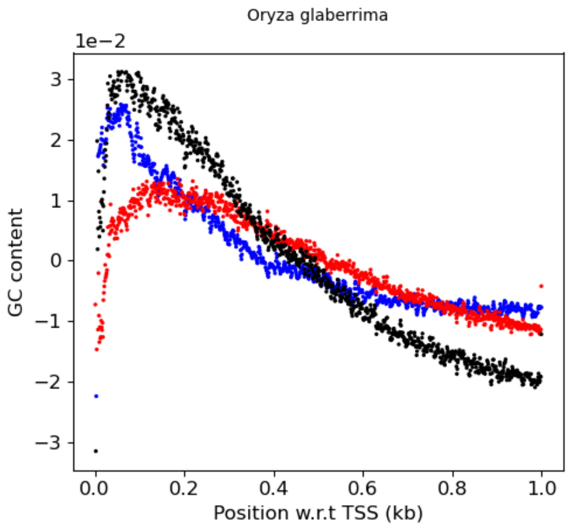
Oryza glaberrima
1e−2
3
2
1
0
−1
−2
−3
GC content
0.0 0.2 0.4 0.6 0.8 1.0
Position w.r.t TSS (kb)

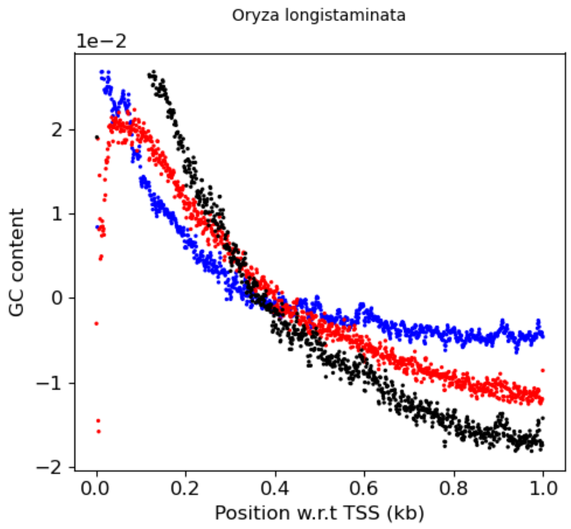
Oryza longistaminata
1e−2
2
1
0
−1
−2
GC content
0.0 0.2 0.4 0.6 0.8 1.0
Position w.r.t TSS (kb)

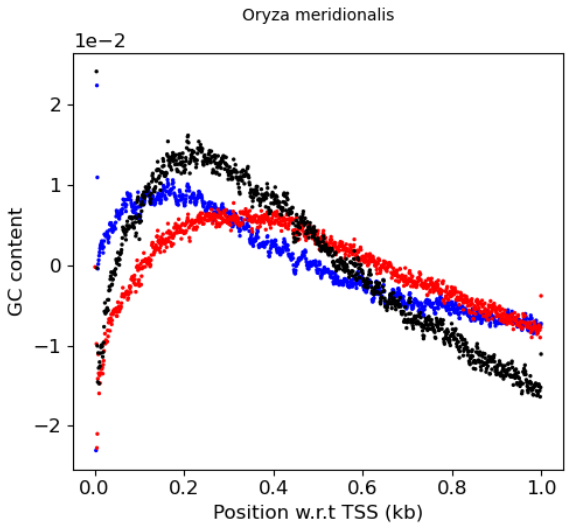
Oryza meridionalis
1e−2
2
1
0
−1
−2
GC content
0.0 0.2 0.4 0.6 0.8 1.0
Position w.r.t TSS (kb)

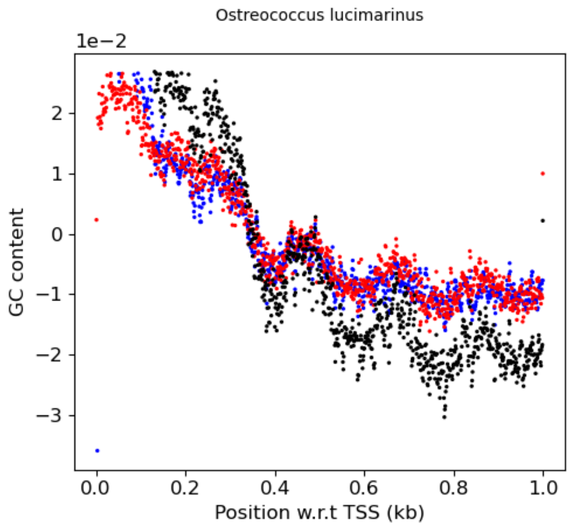
Ostreococcus lucimarinus
1e−2
2
1
0
−1
−2
−3
GC content
0.0 0.2 0.4 0.6 0.8 1.0
Position w.r.t TSS (kb)

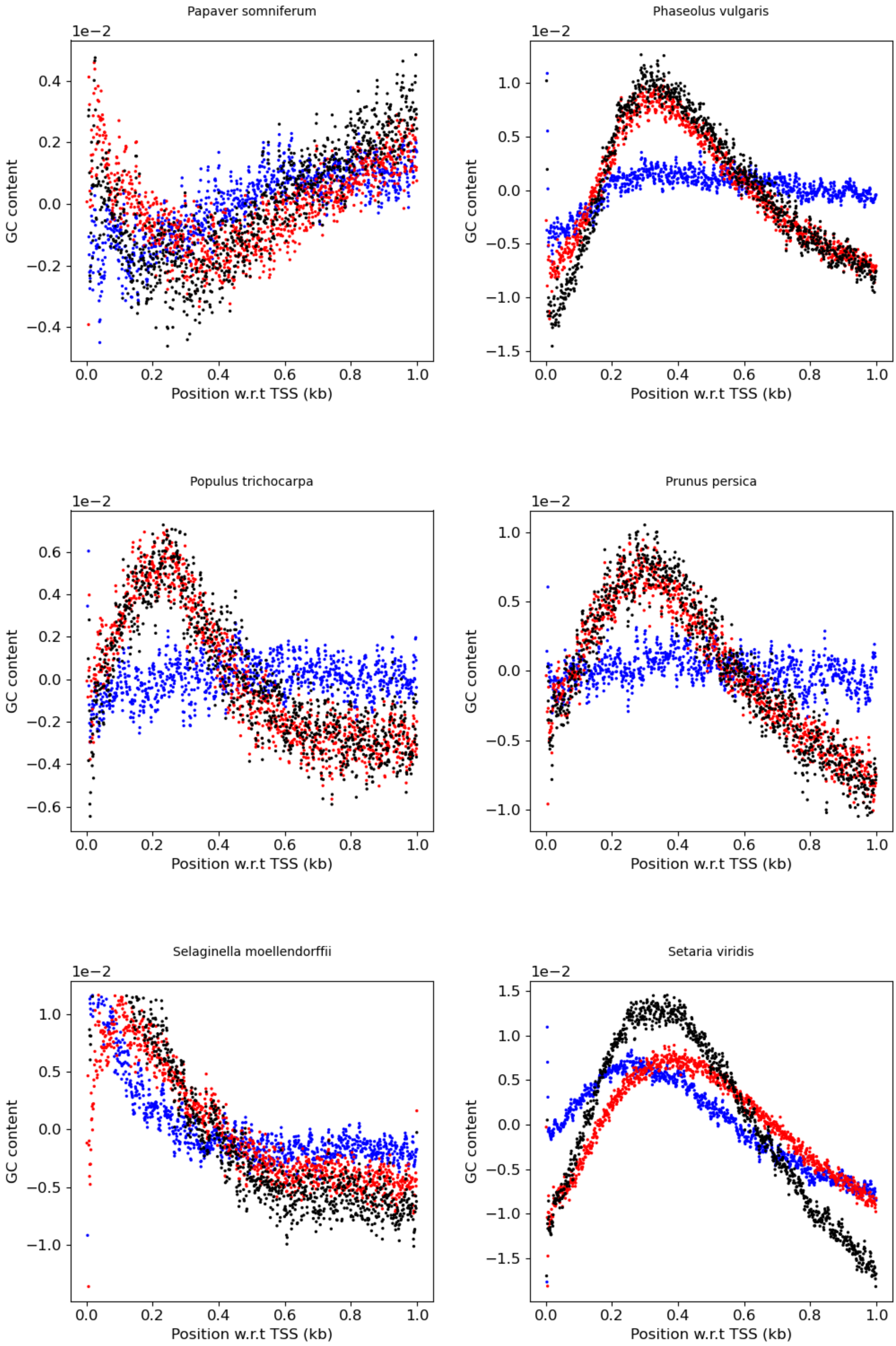
Papaver somniferum
1e−2
GC content
Position w.r.t TSS (kb)
Phaseolus vulgaris
1e−2
GC content
Position w.r.t TSS (kb)
Populus trichocarpa
1e−2
GC content
Position w.r.t TSS (kb)
Prunus persica
1e−2
GC content
Position w.r.t TSS (kb)
Selaginella moellendorffii
1e−2
GC content
Position w.r.t TSS (kb)
Setaria viridis
1e−2
GC content
Position w.r.t TSS (kb)

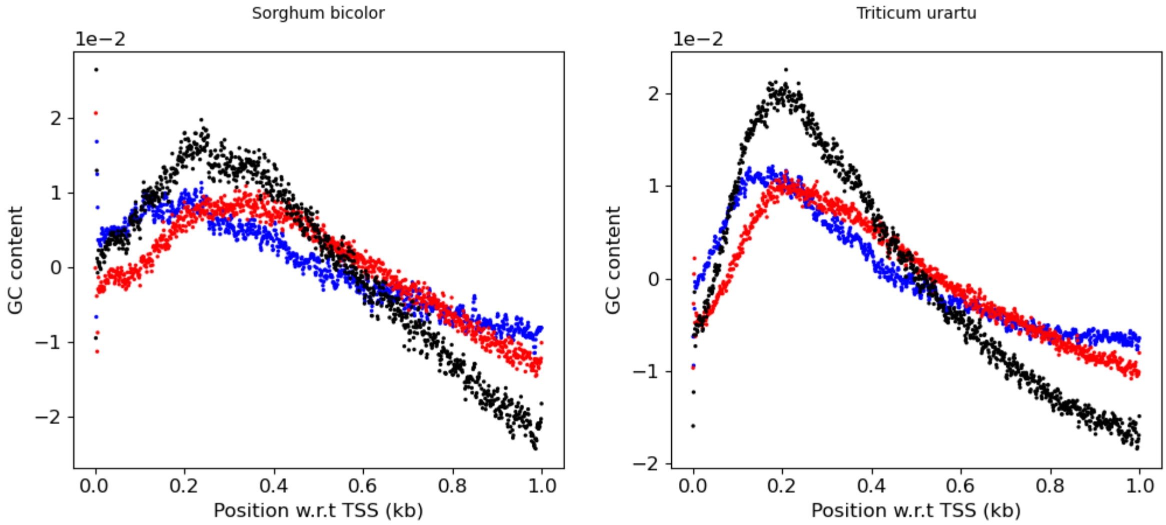
Sorghum bicolor
1e−2
2
1
0
−1
−2
GC content
0.0
0.2
0.4
0.6
0.8
1.0
Position w.r.t TSS (kb)
Triticum urartu
1e−2
2
1
0
−1
−2
GC content
0.0
0.2
0.4
0.6
0.8
1.0
Position w.r.t TSS (kb)
